# Regulated HSPG Signaling Directs Epicardial Behavior to Support Cardiac Formation

**DOI:** 10.1161/CIRCRESAHA.125.327330

**Published:** 2026-08-25

**Authors:** Andia N. Redpath, Irina-Elena Lupu, Louis Haffreingue, Quang M. Dang, Ian R. McCracken, Tamara Carsana, Toin H. van Kuppevelt, Joaquim Miguel Vieira, Nicola Smart

**Affiliations:** 1 Department of Physiology, Anatomy & Genetics, Institute of Developmental and Regenerative Medicine, University of Oxford, United Kingdom (A.N.R., I.-E.L., L.H., Q.M.D., I.R.M., T.C., N.S.).; 2 Division of Medical Genetics & Rare Diseases, High-Tech Center, Vinmec International Healthcare System, Hanoi, Vietnam (Q.M.D.).; 3 Department of Biochemistry, Radboud University Medical Center, Nijmegen, the Netherlands (T.H.v.K.).; 4 School of Cardiovascular & Metabolic Medicine & Sciences, King’s College London, United Kingdom (J.M.V.).

**Keywords:** epithelial-mesenchymal transition, heart, heparan sulfate proteoglycans, morphogenesis, sulfatases, transforming growth factors

## Abstract

**BACKGROUND:**

Pathways controlling cardiac cell behavior share a common dependency on heparan sulfate proteoglycans (HSPGs), which tightly regulate signaling at extracellular locations. This signaling is essential for cardiac development, yet how HSPGs are regulated in the forming heart is unknown. The epicardium is a rich source of HSPG-dependent signaling and cellular progenitors. We hypothesized that extracellular heparan sulfate modifiers, 6-*O*-endosulfatases, orchestrate progenitor cell behavior to support cardiogenesis.

**METHODS:**

We used single-cell RNA sequencing, microscopy, and flow cytometry–based single-molecule RNA in situ hybridization (ISH) to systematically profile 6-*O*-endosulfatases and target HSPGs in the embryonic mouse heart. Subsequently, we utilized knockout and knockdown models that identified gene associations and a role for the main epicardial 6-*O*-endosulfatase isoform, *Sulfatase (Sulf)1*. Transcriptional regulation of *Sulf1* was assessed using assay for transposase-accessible chromatin (ATAC) and Cleavage Under Targets and Release Using Nuclease (CUT&RUN) with sequencing, luciferase assays, and siRNA, and the impact on epicardial cell behavior was confirmed in vivo and using in vitro functional assays.

**RESULTS:**

Despite identical function, we find that *Sulf1* is expressed in the embryonic epicardium, while *Sulfatase (Sulf)2* is expressed broadly throughout the myocardium. We show that epicardial SULF1 dynamically regulates HSPG sulfation to fine-tune the magnitude and duration of signaling to impact cell fate and cardiac morphogenesis. Single-cell genomics and lineage tracing studies reveal *Sulf1* to be strongly coexpressed with key transcriptional regulator *Wt1* (Wilms tumor 1) in the epicardium, with reduction of both coinciding with epithelial-to-mesenchymal transition and quiescence. CUT&RUN-seq revealed transcriptional control of *Sulf1* by WT1, which directly impacts essential HSPG-dependent downstream signaling. Ligand-receptor interaction predictions and functional assays indicated that FGF (fibroblast growth factor)-2 and TGF-β (transforming growth factor β)–driven processes were governed by this regulatory interaction.

**CONCLUSIONS:**

Our study highlights, for the first time, essential fine-tuning of HSPG-dependent signaling to modulate key processes in heart formation, offering potential insights for therapeutically targeting congenital heart disease and enhancing epicardial proregenerative behaviors.

**Novelty and Significance**

**What Is Known?**

- The epicardium supports heart development by producing signals and progenitor cells that regulate cardiac growth, vascular formation, and tissue organization.
- Multiple signals controlling epicardial behavior depend on heparan sulfate proteoglycans, but how their activity is spatially and temporally regulated in the developing heart is poorly understood.
- WT1 (Wilms tumor 1) is essential for epicardial function, yet the mechanisms through which it controls responses to extracellular signals remain incompletely defined.

**What New Information Does This Article Contribute?**

- SULF1 (Sulfatase 1) is identified as the predominant heparan sulfate–modifying enzyme in the embryonic epicardium and is required for normal epicardial formation.
- WT1 directly promotes *Sulf1* transcription, linking an epicardial gene-regulatory program to extracellular control of growth factor signaling.
- The WT1-SULF1 pathway adjusts epicardial sensitivity to FGF (fibroblast growth factor) and TGF-β (transforming growth factor β) signals to regulate proliferation and epithelial-to-mesenchymal transition during heart formation.

The epicardium must respond appropriately to changing developmental signals, but the mechanisms that fine-tune these responses were unknown. This study shows that the key transcriptional regulator WT1 controls expression of SULF1, an extracellular enzyme that modifies heparan sulfate proteoglycans, and this axis regulates the magnitude and duration of growth factor responses in epicardial cells. SULF1 is enriched in the embryonic epicardium; its loss disrupts epicardial formation and alters signaling-dependent cell behaviors, recapitulating aspects of the WT1 knockout phenotype. The study further demonstrates that reduced WT1 or SULF1 activity increases heparan sulfate proteoglycan sulfation and sensitizes epicardial cells to FGF- and TGF-β-dependent cues that control proliferation and epithelial-to-mesenchymal transition. These findings provide the first direct link between an epicardial transcription factor, extracellular heparan sulfate proteoglycan remodeling, and the coordination of cell behavior during heart development. More broadly, the study establishes extracellular sugar modification as a dynamic regulatory layer in organ formation and provides a framework for investigating how similar mechanisms operate in other developing tissues, congenital heart disease, and epicardial reactivation after cardiac injury.

**Meet the First Author, see p e000770**

The outermost layer of the heart consists of specialized mesothelium, referred to as the epicardium, which originates from a transient structure called the proepicardium.^1,2^ The epicardium fundamentally controls cardiogenesis by producing a plethora of angiogenic and cardiogenic paracrine signals and ECM (extracellular matrix) components, and by contributing essential cell types.^3^ Key to myocardial invasion and contribution is the extensive proliferation of epicardial cells, followed by a highly regulated transformation, epithelial-to-mesenchymal transition (EMT), to give rise to epicardium-derived cells (EPDCs). Although the epicardium is quiescent in the adult, cardiac injury induces a degree of epicardial reactivation, which is necessary for full regeneration in lower vertebrates such as zebrafish.^4^ While partial epicardial reactivation in the mammalian heart augments cardiac repair, healing is limited by inadequate epicardial cell invasion.^5^ Delineating the mechanisms that control proliferation and EMT during development may reveal targets for enhancing epicardium-based repair.

During development, epicardial processes are efficiently stimulated by coordinated growth factor signaling. First, FGFs (fibroblast growth factors) and Wnt family members (WNT) control initial formation,^6–9^ and IGFs (insulin-like growth factors) promote proliferation,^10^ while numerous factors induce EMT, including TGF-β (transforming growth factor β),^11,12^ FGF,^12,13^ and PDGF (platelet-derived growth factor).^12,14^ A common feature of these pathways is their regulation by heparan sulfate proteoglycans (HSPGs), which tightly control growth factor/morphogen signaling pathways.^15^ In brief, HSPGs may modulate signaling by binding growth factors to provide an extracellular depot, recruit them to the cell surface in proximity to their receptors, or facilitate the formation of gradients essential for specification and migration. Membrane-bound HSPGs can act as coreceptors for numerous growth factors, including FGF^16–18^ and TGF-β,^19,20^ by lowering receptor activation thresholds or affecting the duration of signaling.^21^ In heart development, loss-of-function studies revealed roles for HSPGs in cardiac lineage specification,^22,23^ outflow tract and atrioventricular valve formation,^24,25^ and ventricular septation.^26^ These studies evidenced a critical requirement for HSPG proteins in cardiac specification and morphogenesis, albeit only a minor fraction of vertebrate HSPGs were explored.

HSPGs comprise a core protein with IdoA (L-iduronic acid)/GlcA (glucuronic acid)-GlcN (glucosamine) or N-AcetylglucosamineGlcNAc saccharide repeats that are sulfated in various positions. Diversity in the extent and position of heparan sulfate (HS) chain sulfation determines capacity for interaction with growth factors and receptors,^15^ meaning that sulfation status confers intricacy in signaling pathway regulation. The 6-*O*-sulfate moiety, key to promoting ternary complex formation of, for example, FGF-FGFR (FGF receptor)-HS,^27^ is added by heparan sulfate 6-*O*-sulfotransferases. Deficiency of HS-generating enzymes causes a range of cardiac abnormalities, in some cases explained by a disruption in HS-dependent FGF or Bone Morphogenetic Protein (BMP) signaling.^28,29^ Countering the actions of 6-*O*-sulfotransferases, 6-*O*-endosulfatases, Sulfatase (SULF)1 and Sulfatase (SULF)2, remove 6-*O* sulfate moieties from HS chains and are central to the development of the dynamic sugar code postsynthetically and to temporal and regional sulfation patterning in organs.^30,31^ Knockout of individual 6-*O*-endosulfatases has not consistently manifested developmental abnormalities^32–37^ due to nonpenetrance and functional redundancy. However, genetic disruption of either isoform impacted neovascularization via HS-binding VEGF (vascular endothelial growth factor)-A_164_ and heart repair following myocardial infarction,^38^ leading to increased scarring and cardiac dysfunction. Detection of *Sulfatase (Sulf)1* in the injury-reactivated epicardium^39^ establishes the potential for HSPG signaling to profoundly control epicardial cell behavior post-myocardial infarction. Given that extracellular endosulfatases may determine epicardial responses to incoming signals and consequently influence epicardial behavior over the course of heart development, and possibly in disease, the regulation of 6-*O*-endosulfatases and their actions warrants investigation.

In the present study, we systematically profiled 6-*O*-endosulfatases and target HSPGs in the embryonic mouse heart, and identified cell type–specific expression patterns for *Sulf1* and *Sulf2*, which changed over the course of development. We reveal *Sulf1* as the major 6-*O*-endosulfatase expressed in the epicardium, and accordingly, defects in epicardial formation in conditional *Sulf1* knockout mice were detected. We show that *Sulf1* expression closely correlates with transcriptional regulator *Wt1* (Wilms tumor 1), with both downregulating upon EMT and quiescence, and elucidated that WT1 regulates *Sulf1* levels in the epicardium via binding to a *Sulf1* intronic enhancer. Finally, we quantitatively inferred HSPG-dependent signaling during EMT and found that key factors promoting epicardial proliferation and EMT, FGF-2, and TGF-β1 were significantly impacted by the WT1-*Sulf1* regulatory interaction. Collectively, this work presents a mechanism linking transcriptional control of HS sulfation to modulation of growth factor signaling and instruction of cellular responses to correctly orchestrate heart development. Such insights may be important for therapeutically enhancing epicardial cell proliferation and EMT in the adult heart for regeneration.

## Methods

### Data Availability

Detailed description of all experimental procedures and materials can be found in the Supplemental Material. scRNA-seq (single-cell RNA sequencing) (GSE299996), assay for transposase-accessible chromatin (ATAC)-seq (GSE299995), and Cleavage Under Targets and Release Using Nuclease (CUT&RUN)-seq (GSE299998) raw and processed data were deposited in the Gene Expression Omnibus. The data that support the findings of this study are available from the corresponding authors upon reasonable request.

### Animal Models

Mouse strains used include C57BL/6J (Charles River), *Gt(ROSA)26Sor^tm14(CAG-tdTomato)Hze^*/J (MGI ID: 3809524) or *Gt(ROSA)26Sor^tm9(CAG-tdTomato)Hze^*/J (MGI ID: 3809523; Rosa26^tdTomato^), *Wt1^tm2(cre/ERT2)Wtp^*/J (MGI ID: 3801682; Wt1^CreERT2^), and *Sulf1^tm1c(KOMP)Mbp^*. *Rosa26^tdTomato^*; *Wt1*^*CreERT2/*+^ males were bred with *Rosa26^tdTomato^*; *Wt1*^*CreERT2*/+^ females to generate *Wt1* knockout embryos (homozygous *Wt1^CreERT2^*). *Sulf1^tm1a(KOMP)Mbp^* embryonic stem cell clones were obtained from the KOMP Repository at the University of California, Davis. The generated *Sulf1^tm1c(KOMP)Mbp^* floxed allele (Sulf1^fl/fl^) mice were bred to carry *Rosa26^tdTomato^*; *Wt1^CreERT2^* for inducible, tissue-specific lineage tracing and knockout following tamoxifen administration (*Sulf1* iKO [inducible knockout]; deletion allele *Sulf1^tm1d(KOMP)Mbp^*). For embryo collection, mice were paired overnight, and females were checked the next morning for the presence of a vaginal plug. Pregnant females were administered 100-mg/kg body tamoxifen by oral gavage at E9.5, unless stated otherwise.

## Results

### HSPGs and Their Modifiers Demonstrate Distinct Expression in the Developing Heart

Despite their decisive influence over the signaling that underlies cardiac morphogenesis, the expression of HSPGs and the enzymes that give rise to distinct spatiotemporal sulfation patterning have not been well documented in the developing heart. We, therefore, systematically profiled HSPG expression at key embryonic stages E10.5, E13.5, and E17.5. We analyzed the published E10.5 mouse heart scRNA-seq data set^40^ (Figure S1A) and generated E13.5^41^ (Figure S1B) and E17.5 (Figure S1C and S1D) data sets with epicardial lineage tracing (Figure S1E). HSPGs may be divided into full-time and part-time categories, with at least 95% of the heparan sulfate of mammalian extracellular surfaces accounted for by full-time.^42^ These include the membrane-bound syndecans (*Sdc1*-*4*) and glypicans (*Gpc1*-*Gpc6*), ECM/basement membrane localized perlecan (*Hspg2*), agrin (*Agrn*) and collagen XVII (*Col18a1*), and secretory vesicle localized serglycin (*Srgn*). Part-time HSPGs include membrane-bound betaglycan (*Tgfbr3*), epican (*Cd44*), neuropilin-1 (*Nrp1*), and ECM-localized testicans (*Spock1-3*).^21,43^ The 20 HSPGs we interrogated not only demonstrated heterogeneous expression profiles between cardiac cell types but also between embryonic stages, with notable exceptions being *Srgn*, largely expressed in the resident leukocyte population, and *Nrp1* ubiquitously expressed in the heart (except in lymphatic endothelial cells (LECs; Figure S1F). *Col18a1* was primarily expressed in nonmyocyte populations. *Gpc2*, *Gpc5,* and *Spock1-3* were not detected, in keeping with their reported absence in embryonic and adult mouse hearts.^44,45^ We noted *Sdc2*, *Sdc4*, *Nrp1*, *Gpc3*, and *Col18a1* consistently expressed in epicardial cells, while *Sdc4* was downregulated and *Gpc6* upregulated in EPDCs (Figure S1F). These results indicate that HSPGs are dynamically expressed, with cell type–specific expression, and changing tissue composition contributing to HSPG diversity in the developing embryonic heart, findings that were corroborated in an independent MULTI-seq (multiplexing using lipid-tagged indices for single-cell and single-nucleus RNA sequencing) study,^46^ which we analyzed at E10.5, E13.5, and E17.5 (Figure S2A and S2B), for direct comparison.

**Figure 1.**
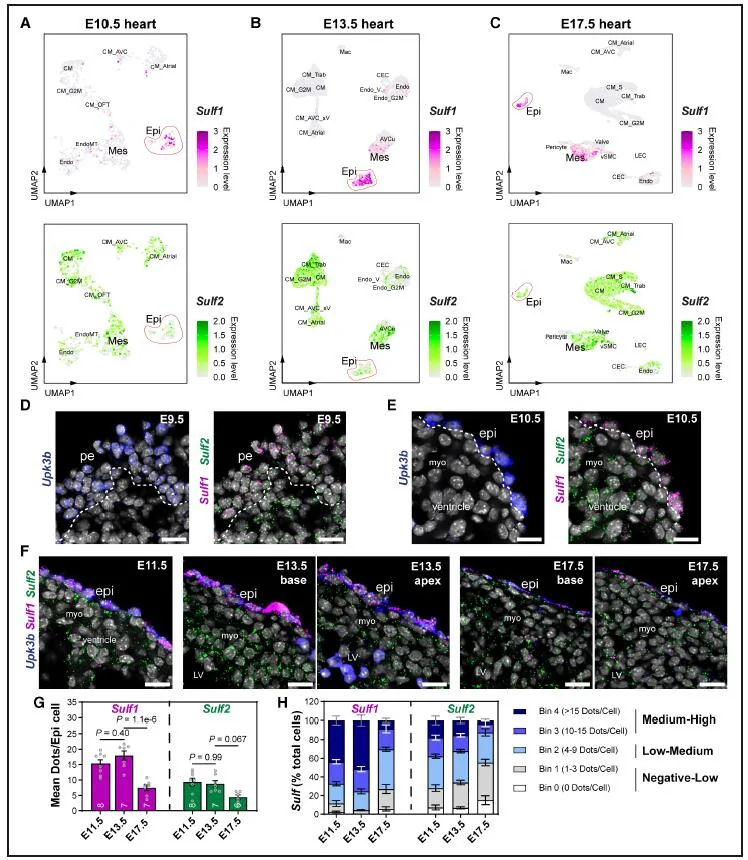
***Sulfatase (Sulf)1* and *Sulfatase (Sulf)2* demonstrate distinct expression profiles in the developing heart. A**, Uniform Manifold Approximation and Projection (UMAP) feature plot showing expression of *Sulf1* and *Sulf2* in E10.5 hearts (928 cells, n=2 batches). **B**, UMAP feature plot showing expression of *Sulf1* and *Sulf2* in E13.5 hearts (6939 cells, n=3 hearts). **C**, UMAP feature plot showing expression of *Sulf1* and *Sulf2* in E17.5 hearts (6341 cells, n=1 heart). **D**, Fluorescence in situ hybridization (ISH) of E9.5 embryos for *Upk3b*, *Sulf1*, and *Sulf2*, showing *Sulf1* in the proepicardium (PE). Scale bars, 20 µm. n=4. **E**, Fluorescence ISH of E10.5 embryos for *Upk3b*, *Sulf1*, and *Sulf2*, showing *Sulf1* in the epicardium. Scale bars, 20 µm. n=7. **F**, Fluorescence ISH of E11.5, E13.5, and E17.5 hearts for *Upk3b*, *Sulf1*, and *Sulf2*, showing *Sulf1* in the epicardium and widespread *Sulf2* in the myocardium. Scale bars, 20 µm. n=9 (E11.5) and n=8 hearts (E13.5 and E17.5). DAPI (4′,6-diamidino-2-phenylindole) nuclear stain was used to visualize individual nuclei. Representative images were chosen according to the following criteria: correct anatomic region, accurate representation of the mean, high quality, and preserved tissue or cellular integrity (**D**–**F**). **G**, *Sulf1* and *Sulf2* expression in ventricular epicardial cells. Data are represented as mean±SEM with individual data points and sample size indicated. One-way ANOVA test with Šídák correction for multiple comparisons (4 comparisons), with adjusted *P* values indicated. **H**, Percentage of epicardial cells expressing *Sulf1* and *Sulf2*, grouped into 5 bins based on the number of dots (individual RNA molecules) per cell. Data are represented as mean±SEM and sample size consistent with **G**. Quantified from fluorescence ISH images (**G**–**H**). AVCu indicates atrioventricular cushion; CEC, coronary endothelial cell; CM, ventricular cardiomyocyte; CM_Atrial, atrial cardiomyocyte; CM_AVC, atrioventricular canal cardiomyocyte; CM_AVC_sV, atrioventricular canal and sinus venosus cardiomyocyte; CM_OFT, outflow tract cardiomyocyte; CM_S, S-G2M phase progressing cardiomyocyte; CM_Trab, trabecular cardiomyocyte; Endo, endocardial cell; Endo_V, valvular endothelial and endocardial cell; EndoMT, endocardial-to-mesenchymal transition; Epi, epicardium; G2M, proliferating; LEC, lymphatic endothelial cell; Mac, macrophage; Mes, mesenchyme; myo, myocardium; pe, proepicardium; Valve, valve mesenchyme; and vSMC, vascular smooth muscle cell.

We next explored expression of 6-*O*-sulfation enzymes. Despite some variation in expression over the 3 embryonic stages, the sulfotransferase isoforms *Hs6st1* and *Hs6st2* were mainly expressed by epicardial cells and cardiomyocytes (Figures S2C, S2D, and S3A). *Hs6st3* expression was not detected in these data sets, supported by the general absence of this isoform in embryonic and adult hearts.^45,47^ Finally, we determined cardiac expression of 6-*O*-endosulfatases, which refine organ-specific sulfation patterning through selective 6-*O* desulfation.^30,31^ We detected enriched *Sulf1* expression in the epicardial clusters at all stages examined, with sporadic and low expression also in endothelial populations and expression within mesenchyme which increased over the course of development (Figure 1A through 1C). In contrast, *Sulf2* was broadly expressed throughout development, with variable levels in most cardiac cell types (Figure 1A through 1C). The dynamic profile of *Sulf1*, increasing from E11.5 to E15.5 before declining, was quantitatively confirmed in whole heart lysates and is in contrast to *Sulf2* expression, which was unchanged (Figure S3B).

Consistent with the epicardial enrichment, we detected *Sulf1* expression as early as E9.5 in the proepicardium (Figure 1D), the origin of epicardial progenitors, and next in the newly formed epicardial layer (Figure 1E; Figure S3E; E10.5). To enable comparison once the epicardium is established, we utilized an E11.5 scRNA-seq data set,^48^ and following data integration (Figure S3C), we inferred a temporal pattern in *Sulf1* expression levels (Figure S3D) in the mixed atrial and ventricular epicardial cell cluster. Epicardial expression of *Sulf1* peaked at E13.5 and diminished by E17.5 (Figure 1F through 1H), although, notably, at E17.5, expression persists at a higher level in the atrial epicardium, in comparison to that of the ventricles (Figure S3F through S3H). Meanwhile, a smaller subset of epicardial cells coexpressed *Sulf2*, with evidently fewer transcripts than *Sulf1* (Figure 1F through 1H), and in accordance with the scRNA-seq, we observed cardiomyocytes as the major source of *Sulf2* in the developing heart (Figure S3E and S3E’). Altogether, these findings demonstrate that 6-*O*-endosulfatase isoforms have distinct expression in the embryonic heart, revealing restricted overlap in some cells. We identified an abundant and localized expression of *Sulf1* in the epicardium, suggesting that it may perform extracellular HS modifications of expressed targets, such as syndecan-4, neuropilin-1, and glypican-3, for epicardial signaling regulation.

### *Sulf1* and *Sulf2* Are Expressed in a Subset of Nonmyocyte Cells Irrespective of Origin

**Figure 2.**
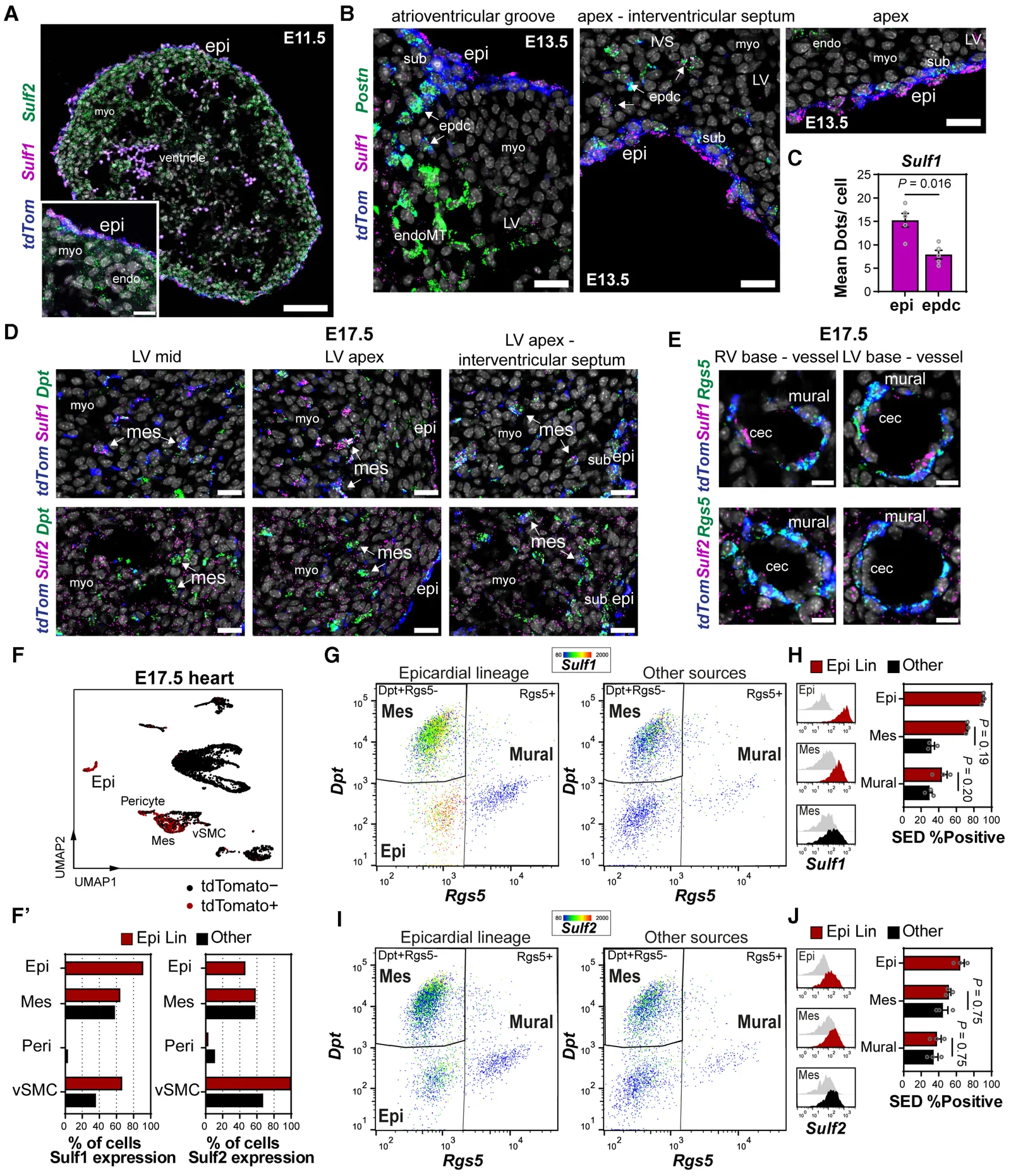
***Sulfatase (Sulf)1* and *Sulfatase (Sulf)2* are expressed in a subset of cardiac mesenchyme and perivascular cells irrespective of origin. A**, Fluorescence in situ hybridization (ISH) of E11.5 hearts for *tdTomato (Tandem Dimer Tomato*), *Sulf1*, and *Sulf2*, showing *Sulf1* enriched in the tdTomato-labeled epicardium of *Wt1^CreERT2^*;*Rosa26*^*tdTomato*^ embryos induced at E9.5. Scale bars, 100 µm; inset, 20 µm. n=6 embryos/hearts. **B**, Fluorescence ISH of E13.5 hearts for *tdTomato*, *Sulf1*, and *Postn*, showing *Sulf1* in epicardial lineage subepicardial mesenchymal cells. Scale bars, 25 µm. n=3 hearts. **C**, *Sulf1* expression in ventricular epicardial and epicardial-derived cells. n=5 hearts. Data are represented as mean±SEM with individual data points indicated. The Mann-Whitney *U* test with *P* values indicated. Quantified from fluorescence ISH images. **D**, Fluorescence ISH of E17.5 hearts for *tdTomato*, *Sulf1*, *Sulf2*, and *Dpt*, showing *Sulf1* and *Sulf2* in epicardium-derived mesenchymal cells invading the myocardium. Scale bars, 20 µm. n=5 hearts. **E**, Fluorescence ISH of E17.5 hearts for *tdTomato*, *Sulf1*, *Sulf2*, and *Rgs5*, showing *Sulf1* and *Sulf2* in epicardium-derived mural cells lining coronary vessels. Scale bars, 10 µm. n=3 hearts. Representative images were chosen according to the following criteria: correct anatomic region, accurate representation of the mean, high quality, and preserved tissue or cellular integrity (**A**–**E**). **F**, Uniform Manifold Approximation and Projection (UMAP) plot showing *tdTomato*-positive cells in the E17.5 heart (739 *tdTomato*+ cells). **F’**, Populations expressing *Sulf1* and *Sulf2*, as a proportion of *tdTomato+* epicardial lineage (Epi Lin) or *tdTomato*− nonepicardial lineage (other). **G** and **H**, Flow cytometric (FC) analysis of E17.5 hearts and quantification of percentage positive cells for *Sulf1*. Color mapping displays levels of *Sulf1*. n=3; pooled 2 hearts per replicate. **I** and **J**, FC analysis of E17.5 hearts and quantification of percentage positive cells for *Sulf2*. Color mapping displays expression levels of *Sulf2*. n=3; pooled 2 hearts per replicate. Gated epicardial lineage as cluster of differentiation (CD)31-cTnT (cardiac troponin T)-tdTomato+ and nonepicardial as CD31-cTnT-tdTomato−, with subsequent gating of mesenchymal cells (Dermatopontin [Dpt]+Rgs [Regulator of G Protein Signaling]5−) and mural cells (Rgs5+). Color map shows the highest expression in red. Fluorescence Minus One (FMO) control histogram in gray. Data are represented as mean±SEM with individual data points indicated. The Mann-Whitney *U* test with Holm-Šídák correction for multiple comparisons (2 comparisons), with adjusted *P* values indicated (**H**–**J**). Cec indicates coronary endothelial cell; endo, endocardium; endoMT, endocardial-to-mesenchymal transition; epdc, epicardium-derived cell; epi, epicardium; IVS, interventricular septum; LV, left ventricle; mes, mesenchymal cell; myo, myocardium; RV, right ventricle; sub, subepicardium; and vsmc, vascular smooth muscle cell.

Given the accumulation of *Sulf1/2*-expressing mesenchyme over the course of development (Figure 1), we sought to determine whether endosulfatase expression in cardiac mesenchyme, fibroblasts, and mural cells was specific to their epicardial origin. We utilized the epicardial lineage tracing capabilities of *Wt1*^*CreERT2/+*^*;Rosa26*^*tdTomato*^ mice (Figure S1E), with tamoxifen administered before embryonic stage E11.5, to label the newly formed epicardium (Figure 2A) and minimize labeling of *Wt1*-expressing endothelium.^2^ The tdTomato (Tandem dimer Tomato)-labeled epicardial layer expressed higher *Sulf1* compared with its early derivatives marked by *tdTomato* and *Postn* coexpression (Figure 2B and 2C; Figure S4A). *Sulf*-expressing epicardium-derived mesenchymal cells were localized in high EMT activity regions, such as the atrioventricular groove and apical subepicardium (Figure 2B; Figure S4A and S4B). By E17.5, we observed an upregulation of *Sulf1* in the tdTomato-labeled mesenchymal cells (*Dpt*+; Figure 2D) that had invaded furthest into the myocardium (Figure S4B’’ and S4C). We also profiled *Sulf* expression alongside *Tcf21*, expressed both in the epicardium and strongly in mesenchymal (fibroblast-forming) cells (Figure S4D), corroborating upregulation of *Sulf1* in the tdTomato-labeled mesenchymal cells (*Tcf21*+; Figure S4E). *Sulf1* and *Sulf2* were detectable, albeit at strikingly low levels, in epicardium-derived vascular smooth muscle cells (*Rgs5* and *Myh11* positive; Figure 2E; Figure S4F and S4G). To assess the origin of mesenchyme and mural cells that express 6-*O*-endosulfatases, we analyzed scRNA-seq (Figure 2F and 2F’; Figure S4G) and performed flow cytometry–based RNA in situ hybridization (Figure 2G through 2J) of E17.5 hearts. Mesenchymal and mural cells were assigned as epicardium-derived (tdTomato+) or originating from an alternative source (tdTomato−), likely the endocardium^49,50^; 40% to 60% of mesenchymal cells and vascular smooth muscle cells expressed 6-*O*-endosulfatases irrespective of their origin (Figure 2F through 2J). However, notably, *Sulf1* was more abundantly expressed (transcripts per cell) in epicardium-derived mesenchymal cells compared with mesenchyme originating from other sources (Figure 2G and 2H). *Tcf21*, although historically considered an epicardial lineage marker, is also highly expressed in nonepicardial-derived mesenchyme^2^ (Figure S4H). We paired *Tcf21,* as previously,^2^ with *Wt1* and lineage tracing to clearly distinguish the epicardium from mesenchymal cells (Figure S4I and S4J), and corroborated our findings above.

*Sulf1* was previously implicated in the regulation of ECs during vascular development and ischemic heart repair.^38,51^ However, when profiling expression in the developing heart, we observed only modest expression in minor subsets within the coronary endothelial and endocardial clusters (Figure S5A through S5C). Specifically, we detected *Sulf1*, coexpressed with *Sulf2,* in coronary ECs (CECs) with arterial macrovascular identity (vascular smooth muscle cell coverage) located at the base of the heart (Figure S4F and S5D) and a subset of atrioventricular valve ECs directly facing blood flow (Figure S5E through S5F’). These atrioventricular valve ECs originate from the endocardium, demonstrated by their expression of *tdTomato* (Figure S5G), in temporally induced *BmxCreERT2*;*Rosa26^tdTomato^*-lineage labeled ECs.^52^ Potentially reflecting a distinct transcriptional driver, only *Sulf2* was detected in lymphatic ECs (Figure S5C). These results demonstrate a restricted expression pattern of 6-*O*-endosulfatase isoforms, in stark contrast to the pan-cardiac EC expression previously reported.^38,51^

### Dysregulated 6-*O*-Endosulfatase Expression Impairs Heart Development

**Figure 3.**
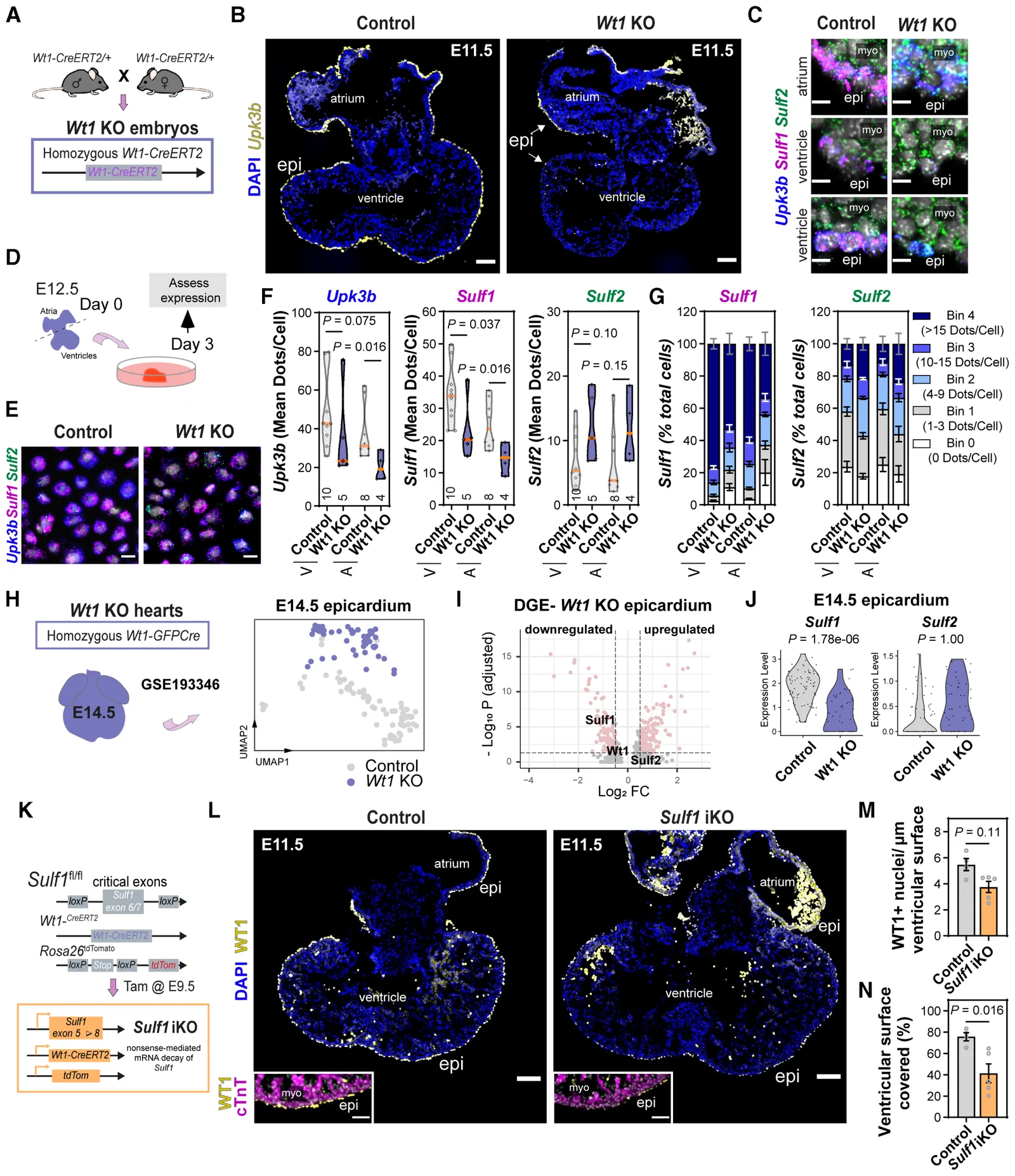
***Wt1* (Wilms tumor 1) knockout (KO) embryos demonstrate dysregulated 6-*O*-endosulfatase expression in the epicardium. A**, Generation of *Wt1* KO embryos. **B**, Fluorescence ISH of E11.5 hearts for *Upk3b* showing a severely disrupted ventricular epicardium in *Wt1* KO hearts. White arrows highlight epicardial cells. Scale bars, 100 µm. **C**, Fluorescence in situ hybridization (ISH) of E11.5 hearts for *Upk3b*, *Sulfatase (Sulf)1*, and *Sulfatase (Sulf)2*, showing *Sulf1* and *Sulf2* in the atrial and ventricular epicardium. Scale bars, 10 µm. Representative images were chosen according to the following criteria: correct anatomic region, accurate representation of the phenotype and mean, high quality, and preserved tissue or cellular integrity. n=6 control and n=5 *Wt1* KO hearts (**B** and **C**). **D**, Generation of atrial and ventricular epicardial explants to assess *Sulf1* and *Sulf2* levels in hearts at E12.5. **E**, Fluorescence ISH of control and *Wt1* KO epicardial explants for *Upk3b,* *Sulf1*, and *Sulf2* and quantification of (**F**) *Upk3b*, *Sulf1*, and *Sulf2*. Scale bars, 20 µm. Violin plot symbol diamond (⋄) represents ventricular (V) explant; cross (+) represents atrial (**A**) explant. Orange line indicates the median. The Mann-Whitney *U* test with *P* values indicated. **G**, Percentage of epicardial cells expressing *Sulf1* and *Sulf2*, grouped into 5 bins based on the number of dots (individual RNA molecules) per cell. Data are represented as mean±SEM; the sample size (explants) per group is indicated in **D** and is identical for **G** (**D**–**G**). **H**, Uniform Manifold Approximation and Projection (UMAP) plot showing the epicardial cluster from control and *Wt1* KO hearts at stage E14.5 (total of 124 epicardial cells, n=1 control and n=2 *Wt1* KO hearts). **I**, Volcano plot showing differential gene expression (DGE) in the *Wt1* KO epicardial cluster compared with control. Genes significantly upregulated or downregulated are colored in pink (filtering criteria adj_p<0.05; log2FC>0.5). *Sulf1*, *Wt1*, and *Sulf2* are annotated. **J**, Violin plot showing relative expression of *Sulf1* and *Sulf2* in the control and *Wt1* KO epicardial cluster. The Wilcoxon rank-sum test with Bonferroni correction for multiple comparisons with adjusted *P* values indicated (**I** and **J**). **K**, Generation of an inducible *Sulf1* KO mouse (*Sulf1* iKO) with lineage tracing capacity; restricted to the epicardial lineage. **L**, Immunofluorescence images showing WT1+ epicardial cells on the surface of control and *Sulf1* iKO hearts at E11.5. Inset shows the region of interest of the left ventricular apex. Scale bar, 100 µm; inset, 50 µm. Representative images were chosen according to the following criteria: correct anatomic region, accurate representation of the phenotype and mean, high quality, and preserved tissue or cellular integrity. Quantification of (**M**) WT1+ epicardial cells and (**N**) coverage on the outer surface of the ventricular wall. n=4 control and n=5 *Sulf1* iKO hearts. Data are represented as mean±SEM with individual data points indicated. The Mann-Whitney *U* test with *P* values indicated (**L**–**N**).

WT1 expression, which is largely reflective of epicardial activity during development, is most abundant in the newly formed epicardium at E11.5,^2^ and its epicardial functions are essential for cardiac development.^53,54^ In contrast to *Sulf2*, epicardial *Wt1* and *Sulf1* expression decreases in levels as embryonic development progresses; *Wt1* downregulates after E11.5,^2^ and *Sulf1* downregulates after E13.5 (Figure 1), to coincide with reduced epicardial proliferation (Figure S6A and S6B). We bred heterozygous carriers of the *Wt1-CreERT2* knock-in, an allele null for WT1,^54^ to generate *Wt1* knockout embryos (*Wt1^CreERT2/ CreERT2^*; Figure 3A); heterozygotes themselves maintain normal *Wt1* levels (Figure S6C through S6E). *Wt1* knockout hearts demonstrated significant disruption, with few epicardial cells lining the E11.5 ventricles (Figure 3B; Figure S6F), consistent with previous studies.^54^ We sought to determine whether defects in epicardial identity and activity associated with loss of WT1 impacted 6-*O*-endosulfatases. Within either the rare ventricular epicardial cells or the more abundant atrial population, *Sulf1* expression was strikingly downregulated, while *Sulf2* was unexpectedly upregulated in *Wt1* knockout hearts (Figure 3C; Figure S6G). These differences were more accurately quantified in E12.5 ventricular and atrial epicardial explants (Figure 3D through 3G), with *Upk3b* to mark epicardial cells (Figure 3E and 3F), as, in contrast to other epicardial markers, it is only moderately reduced in *Wt1* knockout epicardial cells (Figure S6G through S6I). We determined that the proportion of ventricular epicardial cells expressing medium to high levels of *Sulf1* (bin 3 and bin 4) dropped from 86% in controls to 64% in *Wt1* knockout (Mann-Whitney *U* test with Holm-Šídák correction for multiple comparison on composite bins, 2 comparisons, with adjusted *P*=0.0047 for ventricular explants; Table S1), accompanied by a 3.8-fold increase in ventricular epicardial cells with no or lowly detectable *Sulf1* transcripts (bin 0 and bin 1) compared with controls (adjusted *P*=0.0013 for ventricular explants; Table S1). In contrast, the proportion of ventricular epicardial cells expressing medium to high *Sulf2* transcripts increased, albeit not significantly, from 22% in controls to 34% in *Wt1* knockout (adjusted *P*=0.11 for ventricular explants; Table S1), with a corresponding 26% decrease in ventricular epicardial cells that had no or lowly detectable *Sulf2* transcripts compared with controls (Figure 3F through 3G; adjusted *P*=0.11 for ventricular explants; Table S1).

To determine whether altered *Sulf* expression persists to later stages of development, we analyzed the published scRNA-seq dataset^46^ of control and *Wt1* knockout mice (*Wt1*^*GFPCre/GFPCre*^) at E14.5. In line with this study,^46^ the epicardial cluster separated according to mouse genotype (Figure 3H) due to significant changes in the transcriptional profile, including other epicardial markers (Figure S6I) potentially under WT1 control. *Sulf1* was among the significantly downregulated genes in the *Wt1* knockout epicardium, while *Sulf2* was upregulated, albeit not significantly (Figure 3I and 3J; Figure S6H). Overall, these findings show dysregulation of 6-*O*-endosulfases when the epicardium is impaired, with high *Sulf1* and limited *Sulf2* expression associated with normal epicardial formation and activity.

Given the abundance of *Sulf1* throughout the epicardium (Figures 1 and 2), and its significant downregulation coincident with epicardial disruption (Figure 3B through 3J), we questioned the role of Sulf1 in epicardial formation and function. We generated inducible epicardium-restricted *Sulf1* knockout mice (*Sulf1* iKO; *Sulf1*^*fl/fl*^; *Wt1*^*CreERT2/+*^; Tamoxifen at E9.5), with *Rosa26*^*tdTomato*^ lineage reporting (Figure 3K). At E11.5, *Sulf1* iKO hearts displayed a striking disruption in ventricular epicardial formation (Figure 3L), with a reduction in WT1+/tdTomato+ epicardial cells lining the ventricular surface (Figure 3M; Figure S7A), amounting to a significant 46% decrease in coverage (Figure 3N). This demonstrates not only a requirement for *Sulf1* during epicardial formation but also that the *Sulf1* iKO early cardiac phenotype shows striking similarities with that of the *Wt1* knockout. No overt extra-cardiac defects were observed in *Sulf1* iKO (Figure S7B); however, hearts were initially developmentally delayed, were typically smaller compared with controls, with reduced invasion of EPDCs (Figure S7C), and often showed accompanying myocardial compaction defects at E13.5 (Figure S7D and S7E). Although epicardial formation was initially disrupted in *Sulf1* iKO hearts, the epicardial layer appeared fully restored by E13.5, at which point EPDC invasion had ensued, suggesting that the phenotype is transient. Accordingly, in contrast to the *Wt1* knockout embryos, which are embryonically lethal beyond E13.5 to E14.5,^46,54^ *Sulf1* iKO embryos were viable to at least E17.5 (Figure S7F) and showed similar composition of major cardiac cell types compared with controls (Figure S7G), likely due to the mosaic recombination achieved (Figure S7H) when using the CreERT2-*lox* temporal system. Although *Sulf1* was downregulated in both the epicardium and EPDCs (Figure S7I), with levels halved in most cell types relative to controls (Figure S7J; epicardium and mesenchyme: *P*=0.06 and adjusted *P*=0.25), ≈84% of epicardial cells still contained *Sulf1* transcripts (Figure S7J and S7K). Notwithstanding the remarkable capacity for nontargeted epicardial cells to compensate and replace the targeted population,^41^ functional compensation at multiple levels can be envisaged to permit normal HSPG-mediated signaling in the >80% of cells that retain residual *Sulf1* transcript. Taken together, despite the mosaicism and compensation, the transient phenotype shows all the hallmarks of previously reported epicardial defects^54–56^ and suggests a functional role for Sulf1 in the developing epicardium.

### *Wt1* Positively Correlates With *Sulf1* in the Epicardial Lineage

**Figure 4.**
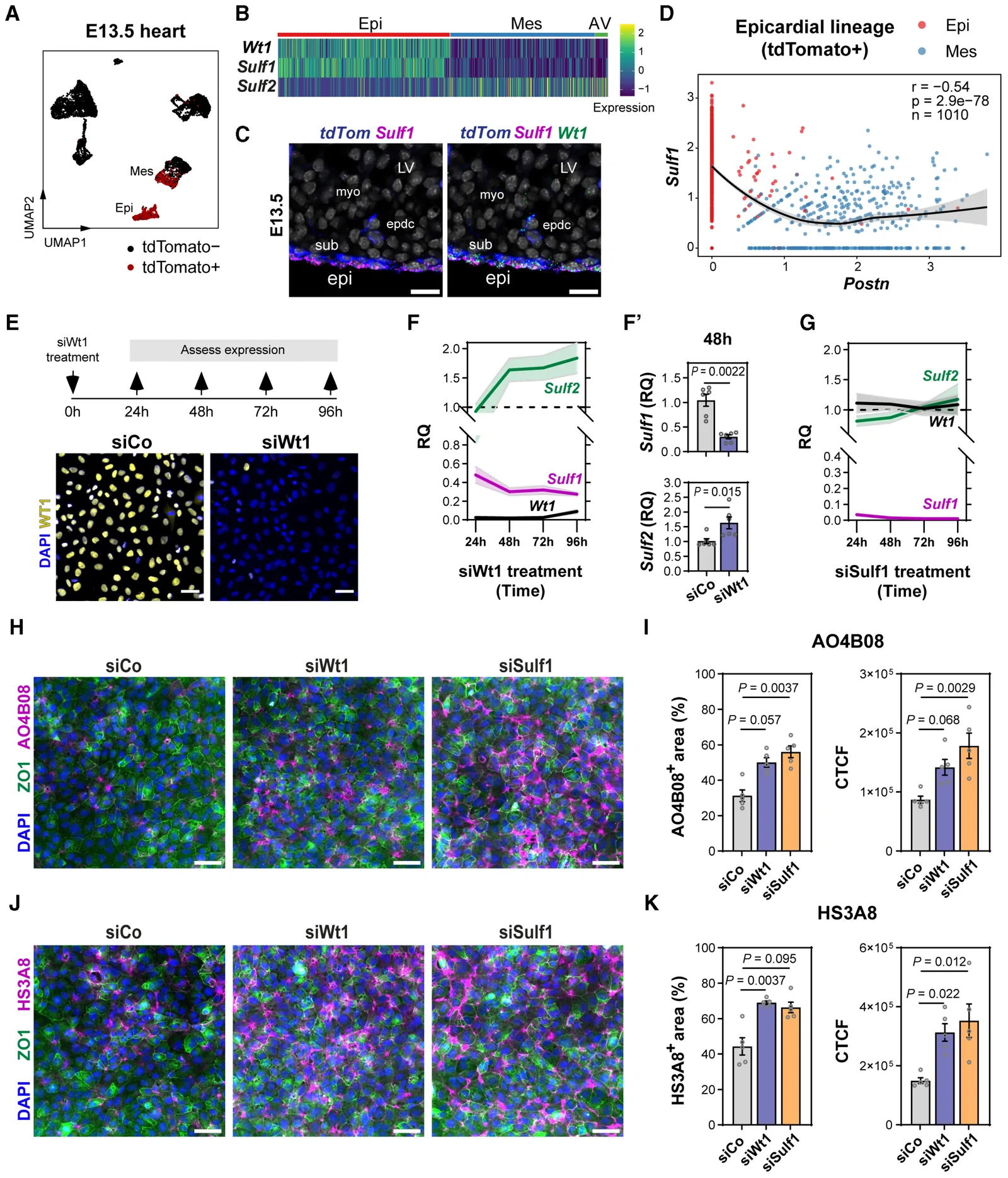
***Wt1* (Wilms tumor 1) positively correlates with *Sulfatase (Sulf)1* expression in the epicardial lineage, and impacts heparan sulfate proteoglycan (HSPG) sulfation. A**, Uniform Manifold Approximation and Projection (UMAP) plot showing *tdTomato (Tandem Dimer Tomato)*+ cells (epicardial lineage) in the E13.5 heart. **B**, Heatmap showing *Wt1*, *Sulf1*, and *Sulfatase (Sulf)2* in the epicardial lineage subset (*tdTomato*+). High expression is indicated in yellow. **C**, Fluorescence in situ hybridization (ISH) of E13.5 hearts for *tdTomato*, *Sulf1*, and *Wt1*, showing downregulated *Wt1* and *Sulf1* in epicardial-derived subepicardial (sub) and mesenchymal cells invading the myocardium (epdc). Scale bars, 20 µm. n=3 hearts. Representative images were chosen according to the following criteria: correct anatomic region, accurate representation of the mean, high quality, and preserved tissue or cellular integrity. **D**, Scatter plot showing quantification of *Sulf1* and *Postn* expression in epicardial and derivative *tdTomato*-positive mesenchymal cells (using E13.5 single cell RNA-sequencing data). A black LOWESS (Locally Weighted Scatterplot Smoothing) line models *Sulf1* expression in epicardium-lineage cells as they upregulate *Postn* expression. Epicardial cells are shown in red, and mesenchymal cells are shown in blue. The Pearson correlation test results annotated. **E**, MEC1 (mouse embryonic epicardial cell line) transfected with control (siCo) or *Wt1* siRNA (siWt1) for 24, 48, 72, and 96 hours. Immunofluorescence images of WT1 showing successful knockdown in *Wt1* siRNA-transfected epicardial cells at 48 hours. Scale bar, 50 µm. **F**, Time-course quantitative real-time polymerase chain reaction (qRT-PCR) analysis of *Wt1*, *Sulf1*, and *Sulf2* relative expression in *Wt1* siRNA-transfected epicardial cells. n=6 experimental replicates for 24, 48, and 72 hours; n=4 experimental replicates for 96 hours. **F’**, Quantification of *Sulf1* and *Sulf2* relative expression at 48 hours. n=6 experimental replicates. Data are represented as mean±SEM with individual data points indicated. The Mann-Whitney *U* test with *P* values indicated. **G**, Time-course qRT-PCR analysis of *Wt1*, *Sulf1*, and *Sulf2* relative expression in *Sulf1* siRNA-transfected epicardial cells. n=6 experimental replicates for 24, 48, and 72 hours; n=4 experimental replicates for 96 hours. Values normalized to *Actb* and *Gapdh* and expressed relative to control (dashed line); shaded data represent mean±SEM (**F** and **G**). **H** through **K**, Immunofluorescence images showing anti-heparan sulfate staining (**H**, AO4B08; **J**, HS3A8) of epicardial cells transfected with siRNAs for 48 hours, and quantification of percentage heparan sulfate-positive area and corrected total cellular fluorescence (CTCF). Scale bars, 50 µm. n=5 experimental replicates. Representative images were chosen according to the following criteria: accurate representation of the mean, high quality, optimal cell density, and preserved cellular integrity. Data are represented as mean±SEM with individual data points indicated. The Kruskal-Wallis test with the Dunn correction for multiple comparisons (2 comparisons), with adjusted *P* values indicated. Epdc indicates epicardium-derived cell; epi, epicardium; LV, left ventricle; myo, myocardium; and sub, subepicardium.

To further explore the relationship between WT1 and 6-*O*-endosulfatases in the epicardial lineage, we assessed expression of these genes at E13.5 when the epicardium is at its most dynamic, undergoing proliferation and extensive EMT,^2^ coinciding with abundant production of factors to support cardiogenesis. Significant perturbation in these essential processes^57^ commonly results in heart failure and embryonic lethality by this stage.^46,54,57^ We subsetted the epicardial lineage (tdTomato+; Figure 4A) and visualized the relative expression of *Wt1*, *Sulf1*, and *Sulf2* in individual cells (Figure 4B). The tdTomato-labeled lineage was composed of an equal proportion of epicardial cells and EPDCs, in line with previous reports.^2^ EPDCs in our scRNA-seq data set are presumed to be the same tdTomato-labeled cells invading the myocardium, enriched in the subepicardium and AVG at E13.5 (Figure S8A), which later contribute to interstitial cells of the ventricular myocardium and atrioventricular valve.^2^ *Wt1* and *Sulf1* strongly colocalized in the epicardium and were consistently both downregulated in EPDCs (Figure 4B) located in the subepicardium and myocardium (Figure 4C; Figure S8A; correlation of declining *Sulf1* with EMT is shown in Figure 4D and with distance migrated shown in Figure S8B). Interestingly, *Sulf2* displayed an inverse correlation, being upregulated in EPDCs (Figure 4B; Figure S8C).

To further assess the dynamics of how WT1 changes 6-*O*-endosulfatases in the epicardial lineage, we utilized RNAi technology and the MEC1 (mouse embryonic epicardial cell) line, derived from E13.5 ventricular epicardial explants and shown to accurately reflect the state and plasticity of the ventricular epicardium.^58^ Temporal knockdown circumvents issues, such as genetic mosaicism, compensation, phenotype penetrance, and indirect regulation, and permits investigation of roles for WT1 and SULF1 in processes that occur beyond epicardial formation. We treated epicardial cells with *Wt1* siRNA and confirmed successful knockdown for up to 96 hours (Figure 4E and 4F). In response to *Wt1* depletion, a 69% reduction in *Sulf1* expression was observed that plateaued at 48 hours (Figure 4F and 4F’), while *Sulf2* expression, from a low baseline level, demonstrated an increase of up to 1.7-fold by 96 hours (Figure 4F), reaffirming the relationship between WT1 and 6-*O*-endosulfatase expression. To exclude the possibility of compensatory activity by the other isoform, we treated epicardial cells with *Sulf1* siRNA and detected no change in *Sulf2* expression (Figure 4G). These results were in line with global *Sulf* knockout studies,^32,34,36,38^ excluding the possibility that *Sulf1* depletion causes compensatory upregulation of *Sulf2* in the epicardium. We used the same approach to validate whether these differential changes occur in primary epicardial cells. After culturing E11.5 atria and ventricles over 24 hours to establish a layer of *Upk3b* and *Wt1*-expressing epicardial cells (Figure S8D), we treated explants with *Wt1* siRNA to achieve 35% to 53% transcript knockdown (proximal-distal location, respectively, with distal showing greater knockdown efficiency) and 87% protein knockdown at 48 hours (Figure S8E through S8G). In accordance with the MEC1 experiments, we identified a 36% reduction in *Sulf1* transcripts and a 2-fold increase in *Sulf2* transcript levels in distally located cells (Figure S8G and S8H). Notably, we observed a change in the distribution of both *Wt1* and *Sulf1* transcripts with *Wt1* knockdown (Figure S8F), represented by predominantly nuclear-restricted mRNA clusters, as opposed to the abundant cytoplasmic puncta in controls (Figure S8I and S8J). Therefore, data in Figure S8G possibly underestimate the reduction in expression, further supported by a greater decrease identified at the protein level (Figure S8E). Taken together, these data implicate 6-*O*-endosulfatases during critical epicardial events and present these as potential gene targets for WT1 in the epicardium.

Extracellular SULF1 and SULF2 remove 6-*O*-sulfate moieties from HS chains.^30,31^ Given that Wt1 depletion leads to downregulation of the predominant endosulfatase isoform, *Sulf1*, we sought to determine the impact on HSPG sulfation in epicardial cells. We analyzed the 6-*O*-sulfate content of HSPGs using phage display anti-HS antibodies, AO4B08 and HS3A8.^59,60^ The AO4B08 antibody, which recognizes both HS and heparin, with strong preference toward 3 consecutive *N*-, 6-*O*-sulfated disaccharide units,^59^ detected a significant increase in HS sulfation with *Sulf1* siRNA treatment, with a similar trend for *Wt1* siRNA treatment (Figure 4H and 4I). The HS3A8 antibody, which recognizes highly sulfated domains within HS with strong preference toward 6-*O*- and *N*-sulfated glucosamine,^60^ detected a significant increase in HS sulfation with both *Wt1* and *Sulf1* knockdown, compared with controls (Figure 4J and 4K), and confirmed that HS sulfation was also increased in the epicardium of *Sulf1* iKO in vivo (Figure S8K and S8L). Overall, these findings show that *Sulf1* depletion in epicardial cells leads to reduced 6-*O*-endosulfatase activity characterized by an increase in sulfated HS chains, an effect that is replicated by reducing WT1 levels.

### WT1 Binds Intronic Cis-Regulatory Element to Enhance *Sulf1* Transcription

**Figure 5.**
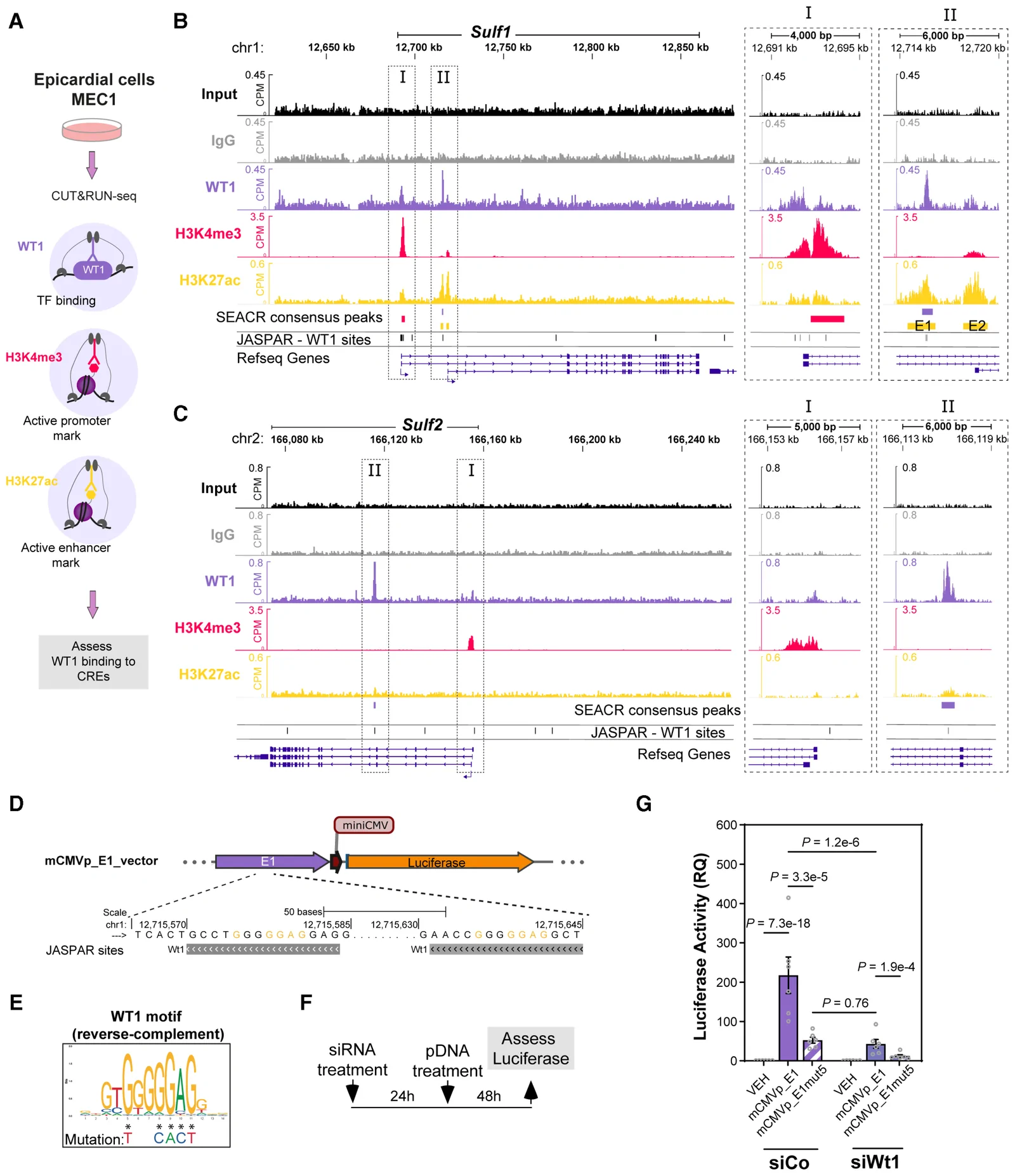
**WT1 (Wilms tumor 1) binds an intronic enhancer proximal to the *Sulfatase (Sulf)1* promoter. A**, Cleavage Under Targets and Release Using Nuclease (CUT&RUN)-seq of MEC1 (mouse embryonic epicardial cells) to assess WT1 transcription factor binding and histone marks. Genome tracks showing binding of WT1 at (**B**) *Sulf1* and (**C**) *Sulfatase (Sulf)2* loci. Track signal is in counts per million (CPM) and merged from n=2 independent replicates. SEACR consensus peaks calculated from n=3 (WT1 and H3K27ac [acetylated lysine 27 of histone H3]) and n=4 (H3K4me3 [methylated lysine 4 of histone H3] and IgG) independent replicates. Region of interest (ROI) indicated in inset I, promoter/transcription start site; inset II, intronic WT1 binding. JASPAR database WT1 binding sites indicated. **D**, Luciferase reporter vector containing intronic enhancer-*Sulf1* fragment E1 upstream of a minimal CMV promoter and Luciferase gene (mCMVp_E1). Two WT1 binding sites located within the E1 fragment sequence. **E**, Five-nucleotide mutation of the WT1 motif located within the enhancer-*Sulf1* fragment E1 (mCMVp_E1mut5) decreases WT1 binding affinity. **F**, MEC1 epicardial cells transfected with control (siCo) or *Wt1* siRNA (siWt1) for 24 hours, followed by transfection with mCMVp_E1 or mCMVp_E1mut5 for 48 hours. **G**, Quantification of luciferase activity to assess transcriptional activity of WT1 on the intronic *Sulf1* enhancer in epicardial cells. n=6 experimental replicates. Values expressed relative to vehicle control. Data are represented as mean±SEM with individual data points indicated. The one-way ANOVA test with Šídák correction for multiple comparisons (5 comparisons) with adjusted *P* values indicated.

The essential requirement for WT1 in cardiogenesis^53^ has been associated, in part, with a proposed role in epicardial EMT.^54,61^ The epithelial cell adhesion protein, E-cadherin (*Cdh1*), and its upstream regulator, *Snai1*, were identified in immortalized epicardial cells as gene targets of WT1.^61^ However, *Cdh1* and *Snai1* were later challenged because both were undetected in the embryonic epicardium, and epicardial EMT appeared to be independent of Snai1.^54,62^ In contrast, bona fide WT1 target genes in nephron progenitor cells have been identified during kidney development,^63^ and notably, 6-*O*-endosulfatases were among the targets identified in these cells.^63–65^ Unlike our findings in the epicardium, WT1 was associated with positive transcription of both 6-*O*-endosulfatases in kidney^63,64,66^ and testis.^67^ In light of this, and the correlations observed in the epicardium, we sought to elucidate whether WT1 regulates 6-*O*-endosulfatases directly, and how this may differ between isoforms. We first assessed the landscape of chromatin accessibility in fluorescence-activated cell sorter–sorted epicardial cells at E13.5 (CD31 [cluster of differentiation 31]^−^tdTomato^+^PDPN^+^; Figure S9A and S9B) and used TOBIAS footprinting^68^ to identify WT1 binding to the *Sulf1* and *Sulf2* loci (Figure S9C and S9D). This predicted WT1 binding to the highly accessible *Sulf1* promoter (Figure S9C), but no binding was predicted for *Sulf2* (Figure S9D). As expected, there was lower accessibility in this locus, reflecting minimal *Sulf2* gene activity in the epicardium. Subsequent CUT&RUN-seq experiments that mapped genome-wide binding sites of WT1 in epicardial cells (Figure 5A) confirmed weak binding to the *Sulf1* promoter although variable across replicates (region of interest I, Figure 5B). In contrast, our analysis revealed significant WT1 binding to an intronic enhancer (E1), marked by active chromatin modification H3K27ac (acetylated lysine 27 of histone H3), located in intron 1 of the *Sulf1* gene (region of interest II, Figure 5B). This region was accessible in E13.5 epicardial cells and contained 2 WT1 binding sites annotated from the JASPAR database (Figure S9E). Next, we applied the activity-by-contact model^69^ incorporating CUT&RUN-seq for histone marks, in vivo ATAC- and RNA-seq data, and we identified a significant interaction between enhancer E1 and the upstream *Sulf1* promoter 1 (Figure S9E and S9F). Based on prominent H3K4me3 (methylated lysine 4 of histone H3) enrichment (Figure 5B; region of interest I; Figure S9E and S9F), promoter 1 appeared to be primarily responsible for expression of *Sulf1* transcript variants 1 and 3 in the epicardium, as opposed to the alternative internal promoter 2, associated with transcript variant 2 (which, nevertheless, generates an identical protein in mice). This assumption was verified by bulk RNA-seq of MEC1 epicardial cells to investigate *Sulf1* transcription in these regions, which revealed a large peak corresponding to promoter 1 (Figure S9F; contributing most of the spliced reads detected in the *Sulf1* locus; Figure S9G) and a small, albeit detectable, peak above background corresponding to promoter 2 (Figure S9F and S9G). Accordingly, transcript variant counts revealed variants 1 and 3 as being more abundant (Figure S9H). Finally, independent corroboration by quantitative real-time polymerase chain reaction confirmed variant 1 as the dominant *Sulf1* transcript in epicardial cells (Figure S9I), with expression of variants 1 and 3 dependent on WT1 (Figure S9J). Interestingly, the CUT&RUN-seq experiments also revealed significant binding of WT1 to the third intron of the *Sulf2* gene (Figure 5C), displaying chromatin accessibility (Figure S9D) without any H3K27ac histone marks or gene activity (region of interest II; Figure 5C). Although WT1 binding was detected at regulatory regions of several key epicardial genes, including *Upk3b, Upk1b, Tcf21,* *Tbx18*, and *Scx* (Figure S10A), not all exhibited marked transcriptional changes in the *Wt1* knockout (Figure S6I). In cases of mesothelial markers, we found that WT1 binds the promoter of *Upk3b* and a downstream intronic region of *Upk1b*, yet expression of these genes was not suppressed in the absence of WT1. In contrast, *Tcf21*, where WT1 binds a distal upstream enhancer marked by H3K27ac, showed the most pronounced downregulation in *Wt1* knockout (Figures S6I and S10A). These findings indicate that WT1 occupancy at annotated regulatory elements does not uniformly translate into transcriptional dependency or measurable effects on promoter activity.

To validate the functional role of WT1 binding to enhancer E1 within the *Sulf1* gene, we performed reporter assays in cultured epicardial cells, with or without endogenous WT1 (Figure 5D through 5G; Figure S10B). We cloned the enhancer sequence into a luciferase reporter vector (mCMVp [murine cytomegalovirus promoter] E1; Figure 5D). We also generated an alternative construct (mCMVp E1mut5) incorporating a 5-nucleotide mutation in both WT1 motif sites (Figure 5E), predicted to abolish WT1 binding without introducing alternative motifs recognized by epicardial transcription factors. Transfection of epicardial cells with mCMVp E1 demonstrated significant luciferase activity, which was attenuated by mutation of the WT1 binding sites or by knockdown of WT1 using siRNA (Figure 5G; Figure S10B). These data identify enhancer E1 as a WT1-dependent regulatory element that drives transcriptional activation, consistent with a direct role in regulating *Sulf1* expression.

### Epicardial Cells Are Primary Orchestrators and Responders in HSPG-Dependent Signaling

**Figure 6.**
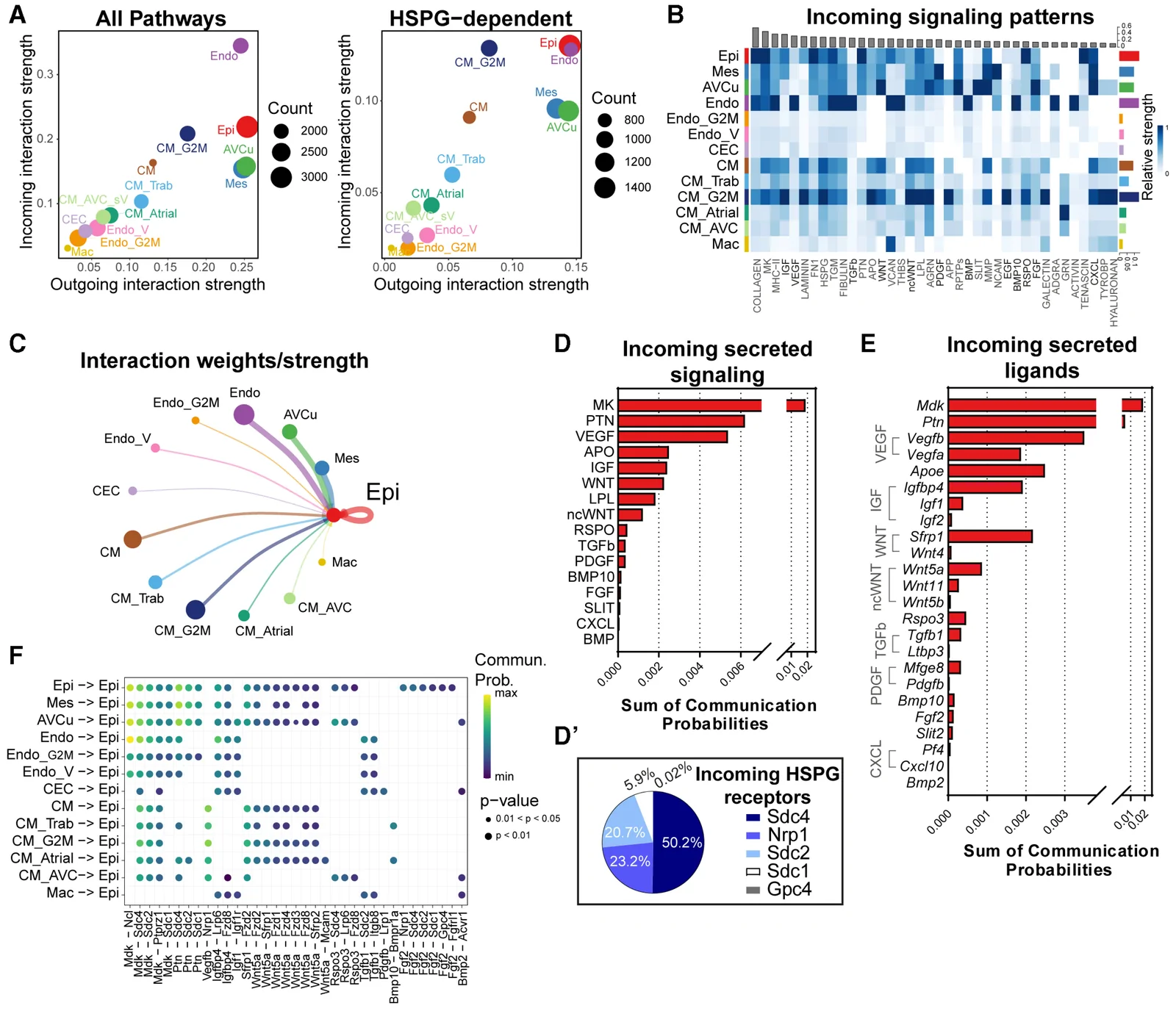
**Heparan sulfate proteoglycan (HSPG)–dependent pathways identified in the E13.5 epicardium. A**, Cell-cell interaction strengths plotted for all cell types, divided into total (all pathways) and HSPG-dependent pathways, indicating incoming and outgoing interactions. **B**, Heatmap and associated bar plots showing most incoming HSPG-dependent signaling patterns. Heatmap represents relative signaling strength of a pathway across cell types. The colored bar plot shows the total signaling strength of a cell type by summarizing all signaling pathways. Gray bar plot shows the total signaling strength of a signaling pathway by summarizing all cell types. High communication probability is indicated in dark blue. **C**, Circle plot of incoming HSPG-dependent signaling networks to the epicardium. **D**, Incoming secreted signaling received by the epicardium ranked from highest to lowest communication probabilities. **D’**, Pie chart showing percentage contribution of HSPG molecules as receptors on the epicardium. **E**, Incoming secreted ligands received by the epicardium ranked from highest to lowest communication probabilities. **F**, Dot plot indicating source cell types communicating with the epicardium and corresponding key ligand-receptor pairs. High communication probability is indicated in yellow; dot size indicates raw *P* values (permutation test; Table S4).

Having demonstrated transcriptional regulation of *Sulf1* by WT1, we next asked whether this regulatory relationship impacts specific HSPG-dependent pathways in the E13.5 epicardium. To infer global HSPG-dependent signaling occurring in the E13.5 heart, we used scRNA-seq and applied CellChat^70^ with an updated database, which we supplemented with literature-supported ligand-receptor interactions, and further annotated pathways associated with HSPG molecules. The epicardium demonstrated the most incoming and outgoing HSPG-dependent signaling interactions, closely followed by the endocardium (Figure 6A). We focused our attention on incoming signaling to the epicardium and identified a broad range of HSPG-dependent pathways (Figure 6B), most of which originated from epicardial, mesenchymal, and endocardial senders (Figure 6C).

Next, we ranked significant incoming signaling (secretory factors) according to the sum of communication probabilities and identified shared pathways involving Midkine, VEGF, and WNT family members and TGF-β in the top 10 (Figure 6D), some of which were shown to signal in the embryonic epicardium,^10,12,54,71^ while others are currently unexplored, or shown to be transmitted, rather than received, by the epicardial lineage.^72–76^ In our analysis, we found that SDC4 (Syndecan 4) and NRP1, expressed on epicardial cells, contributed the majority of HSPG receptor interactions, with SDC4 participating in 50.2% of incoming ligand-HSPG communications (Figure 6D). To prioritize candidate factors for functional validation, we separated pathways into their participant ligands (Figure 6E) and selected factors according to their communication probability scoring and HSPG receptors (Figure 6F; Figure S11A), the expression of which was confirmed in epicardial cells by RNA-seq (Figure S11B). We were also interested in interrogating factors that formed cell type-exclusive interactions, such as VEGFB and BMP10 incoming from cardiomyocytes, PDGFB from CECs, and FGF-2 from epicardial cells (Figure 6F). Our findings suggest that the epicardium is subjected to significant HSPG-dependent signaling at E13.5, mapping a complex communication potential, with SDC4 as principal HSPG partner, and revealing both recognized and new factors that may coordinate cellular responses in this tissue.

### WT1 Regulation of SULF1 Activity Modulates Epicardial Proliferation and EMT

**Figure 7.**
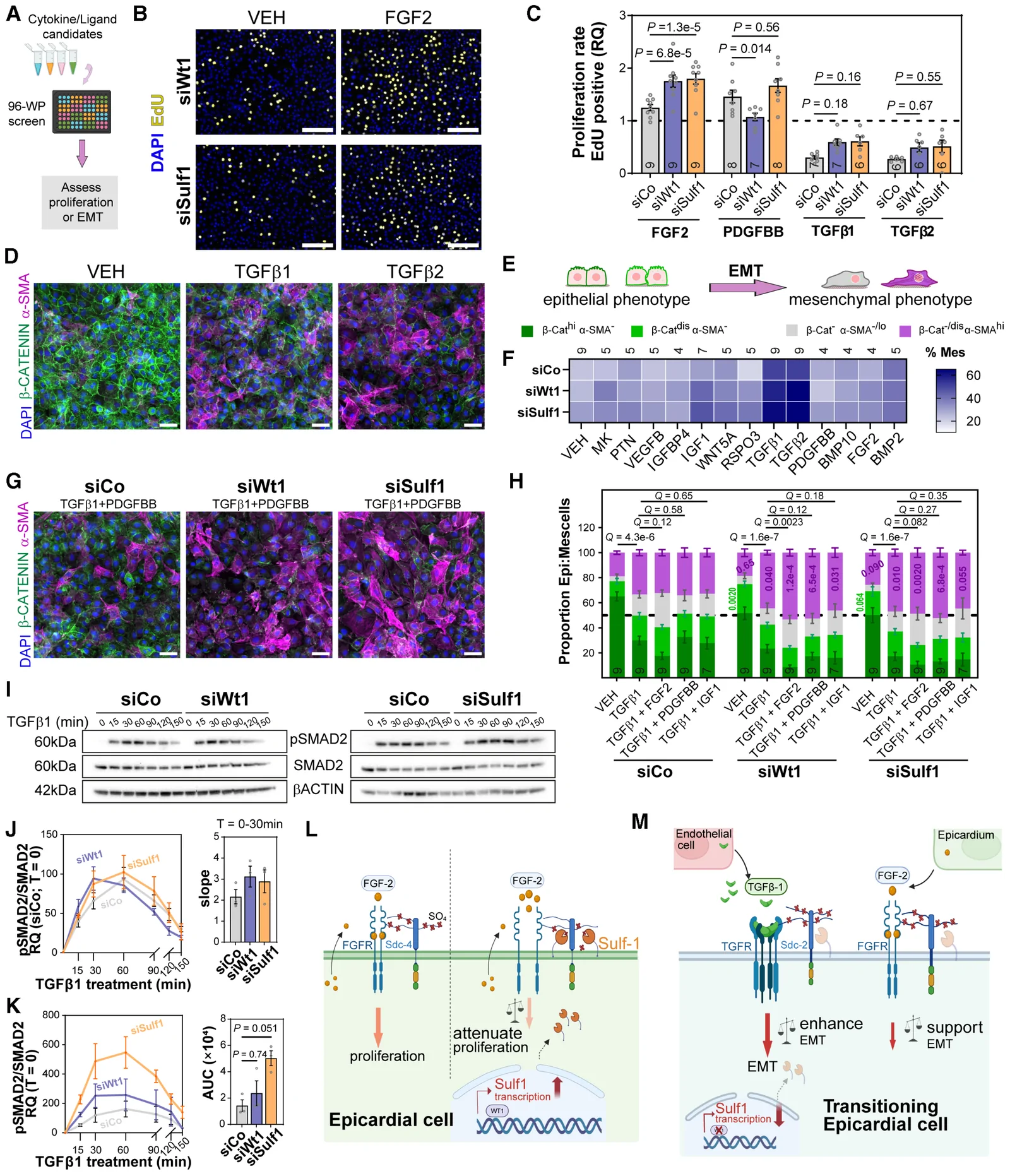
**WT1 (Wilms tumor 1) regulation of SULF1 activity impacts heparan sulfate proteoglycan (HSPG)–dependent pathways modulating epicardial proliferation and epithelial-to-mesenchymal transition (EMT). A**, MEC1 (mouse embryonic epicardial cells) with knockdown of specific target genes were treated with cytokines to assess proliferation and EMT. **B** and **C**, Immunofluorescence images showing EdU (5-ethynyl-2’-deoxyuridine)+ epicardial cells transfected with control (siCo), *Wt1* (siWt1), or *Sulf1* siRNAs (siSulf1) for 48 hours, followed by treatment with cytokines for 24 hours, and quantification of (**C**) the rate of proliferation. Scale bars, 200 µm. n=11 experimental replicates. Representative images were chosen according to the following criteria: accurate representation of the mean, high quality, optimal cell density, and preserved cellular integrity. Values expressed relative to their vehicle control. Data are represented as mean±SEM with individual data points and sample size (experimental replicates) indicated. The two-way ANOVA test with Šídák correction for multiple comparisons (12 comparisons, including siControl+VEH vs siControl+cytokine not shown), with adjusted *P* values indicated. **D** through **F**, Immunofluorescence images showing β-CATENIN and α-SMA (α-smooth muscle actin) staining of epicardial cells transfected with siRNAs for 48 hours, followed by treatment with cytokines for 24 hours, and quantification of (**F**) percentage mesenchymal cells. Scale bars, 50 µm. n=9 experimental replicates. Representative images were chosen according to the following criteria: accurate representation of the mean, high quality, optimal cell density, and preserved cellular integrity. Heatmap data expressed as mean, and sample size (experimental replicates) indicated. The ART ANOVA test with Holm-Šídák correction for multiple comparisons (39 comparisons) with adjusted *P* values in Table S2. **G** and **H**, Immunofluorescence images showing β-CATENIN and α-SMA staining of epicardial cells transfected with siRNAs for 48 hours, followed by treatment with cytokines for 24 hours, and quantification of (**H**) percentage epicardial and mesenchymal cells. Scale bars, 50 µm. n=8 experimental replicates. Representative images were chosen according to the following criteria: accurate representation of the mean, high quality, optimal cell density, and preserved cellular integrity. Data are represented as mean±SEM with sample size (experimental replicates) indicated. The two-way ANOVA test with the Benjamini-Krieger-Yekutieli false discovery rate (FDR) correction for multiple comparisons (12 comparisons for mesenchymal phenotype; 56 comparisons for each cell phenotype), with select *Q* values indicated and found in Table S3. Color of bars represents cell phenotype. Black *Q* values represent mesenchymal cell comparisons between treatment groups, and colored *Q* values represent comparisons between their respective siControl group (eg, siControl+TGF-β [transforming growth factor β] 1 vs siWt1+TGF-β1) for a specific cell phenotype. **I**, Western blot showing SMAD2 (mammalian homolog of Mothers against decapentaplegic 2) signaling in control (siCo), *Wt1* (siWt1), and *Sulf1* (siSulf1) siRNA-transfected epicardial cells (48 hours), followed by treatment with 10-ng/mL TGF-β1 (time response). Blot representative of n=3 experiments. Quantification of SMAD2 signaling expressed relative to (**J**) control siRNA at time=0 and (**K**) corresponding siRNA at time=0. n=3 experiments. Data are represented as mean±SEM. The ART ANOVA test with the Benjamini-Krieger-Yekutieli FDR correction for multiple comparisons (18 comparisons), with *Q* values in Table S6. Total area under the curve (AUC), the Kruskal-Wallis test with the Dunn correction for multiple comparisons (2 comparisons), with adjusted *P* values indicated, and detailed results in Table S8. **L**, Model of transcription factor WT1 positively regulating *Sulf1* expression to temporarily attenuate autocrine FGF (fibroblast growth factor)-2–stimulated proliferation in the epicardium. **M**, Model of WT1 downregulation directly attenuating *Sulf1* expression in transitioning epicardial cells (denoted by semitransparent fill) to promote a robust response to EMT signals coming from endothelial cells, with the EMT process supported by the adjacent FGF-2-expressing epicardium. Schematic in **L** and **M** created in BioRender. Redpath, A.N. (2026) https://BioRender.com/c7z5abu and https://BioRender.com/tdvh6hg.

We interrogated 12 factors shortlisted from our ligand-receptor pair analysis based on properties described above, along with TGF-β2 as a positive control.^14^ We first questioned how these factors influence epicardial proliferation and EMT, and assessed their activity in the presence or absence of WT1 and SULF1. We treated epicardial cells with *Wt1* or *Sulf1* siRNA, to reduce SULF1 activity, before treating with selected factors and assessing proliferation rate using EdU (5-ethynyl-2’-deoxyuridine) incorporation (Figure 7A). Only FGF-2 promoted epicardial proliferation (1.3-fold increase), while TGF-β and BMP10 decreased proliferation by 75% and 38%, respectively (Figure S11C; Table S7). We noted that *Wt1* and *Sulf1* knockdown alone reduced EdU+ cells by 35% to 37%; however, both demonstrated a significant increase in the proliferation rate upon FGF-2 stimulation compared with control transfection (Figure 7B and 7C). Taken together, *Sulf1* depletion elicits a stronger proliferative response to FGF-2 in epicardial cells, verifying an HSPG-dependent pathway impacted by the WT1-*Sulf1* regulatory relationship.

We used a similar approach to assess the impact on epicardial cell transition and differentiation by screening the same 12 factors and quantifying mesenchymal derivatives. We immunostained epicardial cells with β-CATENIN, an intracellular component of anchoring junctions that enable interaction with neighboring cells,^77^ and a classic hallmark of epithelial monolayers. We costained for α-SMA (α-smooth muscle actin) to label actin filaments, characteristic of cells adopting a mesenchymal phenotype with spindle-shaped morphology (Figure 7D and 7E). TGF-β treatment significantly increased the percentage of mesenchymal cells, with 50% in the control siRNA supplemented with TGF-β1 (adjusted *P*=2.2×10^−4^; Table S2), which was augmented to 58% and 63% with *Wt1* and *Sulf1* knockdown, respectively (Figure 7F; adjusted *P*=1.1×10^−^^4^ and *P*=1.8×10^−^^3^; Table S2; Figure S11D). Epicardial cells with reduced SULF1 activity also demonstrated a modest increase in EMT with IGF1 (Insulin-like Growth Factor 1) treatment (Figure S11D). Considering other factors present in the heart could influence cell fate decision once EMT is induced, we next examined TGF-β1 in combination with other shortlisted HSPG-dependent factors (Figure 7G and 7H) and found that cotreatment with FGF-2 modestly expands the proportion of mesenchymal cells displaying complete loss of β-CATENIN without α-SMA upregulation (gray; Figure 7H), albeit this is not statistically significant (*Q*=0.25; Table S3). Interestingly, we detected a 1.9- and 1.6-fold increase, respectively, in the proportion of epicardial cells with disrupted cell-cell contacts (light green) in response to *Wt1* and *Sulf1* knockdown alone (Figure 7H; *Q*=0.0020 and *Q*=0.064; Table S3). Disruption in epicardial cell-cell contacts was previously reported following *Wt1* knockdown in epicardial explants derived from hearts at E12.5.^78^ Furthermore, *Wt1* knockdown in human adult epicardial cells demonstrated morphological changes that suggested greater spontaneous EMT in vitro.^79^ These data consistently support a role for WT1 in maintaining epithelial state and implicate Sulf1 as a key downstream effector. Both *Wt1* and *Sulf1* knockdown enhanced the proportion of α-SMA-high mesenchymal cells following FGF-2, PDGFBB, and IGF1 combinatorial treatments, with the greatest increase in EMT associated with FGF-2/TGF-β in combination (Figure 7H). We concluded that *Sulf1* depletion elicits a robust response to TGF-β in epicardial cells, enhancing EMT, and further permitting FGF-2 to aid in the process.

We validated the expression domains of *Tgfb1*, *Tgfb2*, and *Fgf2* by fluorescent ISH staining and assessed the positioning of signaling cells in the E13.5 heart (Figure S11E through S11G), particularly with regard to their proximity to the epicardium and EPDCs. scRNA-seq analysis showed that ECs and macrophages express *Tgfb1* (Figure S11E), and we further validated abundant expression of this factor in the endocardium and subepicardial domain (Figure S11E’). Indeed, our ligand-receptor inference analysis highlighted that most significant TGF-β1 communications to the epicardium (Figure 6F; Figure S11A; Table S4) originated from the nonproliferative endocardial cluster and CECs. *Pecam1*+ CECs sprouting from the sinus venosus at the base of the heart typically run along the subepicardial space (Figure S11E’) before invading the compact myocardium.^80^ Our data demonstrate CEC proximity to the tdTomato-labeled epicardium and derivative mesenchymal cells, even showing side-by-side alignment with *Tgfb1*-expressing CECs in the interventricular septum (Figure S11E’). In contrast, we found broad *Tgfb2* expression throughout cardiac cell types (Figure S11F). We confirmed abundant expression in the epicardium, cardiomyocytes, and mesenchyme of the atrioventricular groove and cushion. Interestingly, expression of *Tgfb2* in regions with high EMT activity was mainly contributed by the EPDCs themselves (right inset, Figure S11F’). Considering the abundance of *Tgfb2*, we asked why this known inducer of EMT^11,14^ was not predicted in our analysis of HSPG-dependent pathways. We identified that although ≈20% to 37% of the E13.5 epicardium expresses the requisite TGF-β receptors 1 and 2 (Figure S12A), it does not express the HSPG coreceptor *Tgfbr3* (Figures S1F and S2A) essential for TGF-β2 binding to the receptors.^81^ In contrast, *Tgfbr3* is expressed in cultured epicardial cells, supported by the enrichment of H3K4me3 at the *Tgfbr3* promoter (Figure S12B) and detection by RNA-seq (Figure S12C). This is consistent with previously reported gene expression^14^ in MEC1 cells, which may explain their ability to respond to this factor in vitro. We concluded that TGF-β1, expressed by ECs, as opposed to TGF-β2, is likely to promote EMT and support the transition of derivative mesenchymal cells. We further identified that the epicardium is the sole source of incoming FGF communication (*Fgf2*) at E13.5 (Figures S11G and S11G’ and S12D through S12G), suggesting autocrine regulation of its proliferation. It should be noted, however, that the initial proliferation that underpins epicardial formation at E10.5 appears to be FGF-2-independent, given the limited expression of this ligand in the heart at this stage (Figure S12D and S12E). Instead, previously reported roles for BMP and WNT signaling are supported by enhanced incoming signals from cardiomyocytes, including BMP2 and WNT2 (Figure S12F and S12H; Table S5). We also infer strong cardiomyocyte-derived FGF signals from mitogens, such as FGF1 and FGF9 (Figure S12G and S12H; Table S5), which may contribute to epicardial expansion despite their less well-characterized roles in epicardial layer formation. Taken together, epicardial-expressed SULF1 is important in both paracrine and autocrine signaling cues, which regulate proliferation and contribution to the developing heart in a temporally regulated manner.

While both FGF-2^27^ and TGF-β1^82^ directly bind HS, the mechanism by which HS 6-*O* regulates their signaling differs. Nonetheless, 6-*O*-sulfation was required for FGF-2-FGFR1 signaling in mouse embryonic fibroblasts and Human Umbilical Vein Endothelial Cells^27,83^ and for TGF-β1 signaling in lung fibroblasts.^84^ Lastly, therefore, we interrogated how transcriptional regulation of *Sulf1* by WT1 may impact the downstream HSPG-dependent signaling response, to bring about the observed functional changes in epicardial cells, which suggest a mechanism of growth factor sensitization. We examined TGF-β1 signaling in epicardial cells and found that reduced SULF1 activity, achieved by either *Wt1* or *Sulf1* knockdown, enhanced SMAD2 (mammalian homolog of Mothers against decapentaplegic 2) phosphorylation (Figure 7I). Intriguingly, our analysis showed that both the rate of increase in the first 30 minutes of TGF-β1 treatment (Figure 7J; all relative to siControl at T=0) and overall magnitude of phospho-SMAD2 signaling (Figure 7K; relative to their respective siRNA treatment at T=0; *Q*=0.023; Tables S6 and S8) were enhanced under *Sulf1* knockdown conditions. These data support enhanced TGF-β1 signaling with reduced SULF1 activity and suggest that the WT1-*Sulf1* regulatory interaction may influence epicardial cell response to EMT-inducing factors.

## Discussion

Impairing epicardial function, by genetic deletion of key transcription factors^46,54,85^ and essential signaling,^57,74,86,87^ or by direct ablation,^88,89^ proves detrimental to both heart development and repair in disease. Despite improvements in our understanding of embryonic epicardial properties, little is known about how the epicardium deciphers signaling cues to temporally and spatially orchestrate cellular processes. In this study, we explored 6-*O*-endosulfatases capable of modifying the sugar code extracellularly and uncovered a transcriptional regulatory relationship between the critical epicardial regulator WT1 and 6-*O*-endosulfatase isoform *Sulf1*. We propose that this relationship instructs changes in HS sulfation, to adjust epicardial lineage sensitivity to signaling cues, and to modulate cellular responses during heart development (Figure 7L and 7M).

Before exploring this regulatory relationship in detail, we first delineated *Sulf1* and *Sulf2* cell type–specific expression patterns, which, along with the HSPG diversity we observed in the embryonic heart, reinforce the notion of temporal and regional HS patterning in organs dynamically changing over the course of development.^30,31^ Here, we recognized cardiomyocytes as the major source of Sulf2, while an abundant and localized expression of *Sulf1* was detected in the epicardium. Our finding that 6-*O*-endosulfatase isoforms show distinct expression profiles in the embryonic heart, with only a minor overlap, supports their functional diversity in vivo.^32,37^ Embryonic lung, gonads, and eyes similarly show nonoverlapping expression domains.^33,90^ Notably, enriched *Sulf1* expression in the epicardium builds upon evidence that this isoform is commonly found in embryonic mesothelia, epithelia, and connective tissue lining organs, with functions akin to the epicardium during development. Examples include lung pleura,^33,35,90^ pericardium,^33,90^ pancreatic mesothelium,^90^ and periosteum.^90^ Conservation between species is likely as *Sulf1* homologs are enriched in the *Xenopus* pericardium^91^ and human epicardium.^92^ *Sulf1* transcription in visceral mesothelia, such as that of lung and pancreas, may also be subject to control by WT1 during embryogenesis. Thus, the modulation of cellular processes by the WT1-*Sulf1* regulatory relationship that we describe here may apply more broadly across other organs and species.

Our finding that loss of WT1 in epicardial cells confers an incomplete loss of Sulf1 supports the notion that WT1 acts by enhancing *Sulf1* expression and aligns with other reports in mesonephros and podocytes.^64,66,93^ Our data offer improved mapping of WT1-DNA binding sites in the *Sulf1* locus, as well as across the genome. When paired with histone mark information, we were able to identify that WT1 transcriptional regulatory activity is exerted through direct binding to an intronic enhancer, a region interacting with the *Sulf1* promoter. Intriguingly, a similar region was identified in adult glomeruli with high placental mammal conservation.^93^ In this study, we uncovered striking differences in expression of 6-*O*-endosulfatase isoforms between the epicardium and its early mesenchymal derivatives. Specifically, *Sulf1* was downregulated, while *Sulf2* was upregulated at the time of transition. We went on to elaborate the relationship between *Wt1* and *Sulf1* following EMT, finding it comparable to human fetal hearts, where both genes downregulate in EPDCs.^92,94^ In contrast, immature podocytes and adult sertoli cells feature continued activation of both 6-*O*-endosulfatase isoforms by direct binding of WT1 to their promoters.^63,64,67^ We propose that Sulf2 takes on a lesser role in the epicardium, as fewer epicardial cells express this isoform and do so at lower levels. Unlike *Sulf1* transcription, expression appears independent of WT1. Rather, an inverse relationship exists between *Wt1* and *Sulf2* in the epicardial lineage, which we speculate results from adoption of mesenchymal identity. Insights from cancer research may be relevant, as Sulf2 is frequently associated with the terminal mesenchymal state in EMT and, as such, underpins the invasive nature of these cells in tumor metastasis.^95^

Despite challenges in studying HS-modifying enzymes in vivo,^31,33,34,36,37^ we identified a defect in the formation of the epicardium with *Sulf1* deficiency, likely due to dysregulated BMP, FGF, and WNT signaling during early epicardial specification.^6,9,96^ While SULF1 activity may limit FGF, it is required for BMP and WNT signaling.^97,98^ Furthermore, the impaired epicardial phenotype we observed in *Sulf1* iKO hearts partly mimicked the cardiac phenotype reported for *Wt1* nulls,^46,54^ strengthening the functional association between these 2 genes. In clinical cases where mutations in *WT1* underlie inherited disease, dysregulated cellular processes during kidney development associate with downregulation of *SULF1* in patient cells.^99^ This raises questions about whether congenital heart defects associated with *WT1* mutations,^100,101^ although rare, involve disrupted SULF1 activity in the epicardial lineage.

Here, we identified dynamic epicardial *Sulf1* expression, with peak levels at E13.5 coinciding with shifts in environmental signals and cellular processes. In line with others,^102^ we identify that the E13.5 epicardium is less proliferative overall and propose that Sulf1 temporally limits autocrine FGF-2-stimulated proliferation. In *Drosophila*, Sulf1 ensures termination of intestinal stem cell division and return to homeostasis, as it limits mitogenic pathways initially required in the regenerative response.^103^ An enhanced mitogenic response to FGF-2 upon *Sulf1* downregulation was reported in embryonic and cancer cells^31,34,104,105^ and supports our findings of sensitization in *Sulf1*-depleted epicardial cells.

6-*O*-endosulfatases have been implicated in regulating cell fate in a variety of stem and progenitor cell niches.^35,36,106–108^ We and others have identified that the epicardium undergoes EMT over a limited developmental window, with apparent regionalized waves of invasion, first appearing at the atrioventricular grooves around E11.5 and becoming more widespread after E13.5.^2,109,110^ Here, we identify that TGF-β1 signaling in the epicardium is impacted by the WT1-*Sulf1* regulatory relationship. We show that over a third of the epicardial population expresses TGF-β receptors required to respond to the distinct sources of TGF-β1 in vivo. We propose that the endocardium forms a gradient of TGF-β1 in the ventricular myocardium, and transient downregulation of *Sulf1* in EPDCs promotes a robust response to EMT signals, with their transition supported by the adjacent TGF-β1/PDGFB-expressing coronary endothelium and FGF-2-expressing epicardium. With downregulation of WT1 lying upstream of this process, the *Sulf1*-low epicardial lineage, thus, becomes more sensitized to EMT factors, leading to elevated intracellular signaling. Indeed, increased TGF-β1 signaling was previously reported in *Sulf1*-deficient lung fibroblasts, allowing for enhanced transdifferentiation to myofibroblasts.^111^ Curiously, at the final stages of cardiac development, epicardium-derived mesenchymal cells situated deeper within the myocardium, compared with those more superficially, expressed higher levels of *Sulf1*, possibly a consequence of establishing fibroblast cell fate.^38,112^

The membrane-bound localization of 6-*O*-endosulfatases urges us to recognize that their impact on HSPG-dependent signaling is not restricted to autoregulatory epicardial processes. Our identification of the epicardium as the major source of outgoing HSPG-dependent signaling and SULF1 activity raises questions as to how this may influence paracrine signaling to diverse adjacent cell types. For example, SULF1 activity was suggested to enhance the angiogenic effects of CXCL12,^38^ a factor secreted by the epicardium and important in coronary arteriogenesis during development.^52,113^

The epicardium seems to lose its plasticity in adulthood and is considered largely quiescent under physiological conditions. Although in injured hearts upregulated *Sulf1* expression is largely contributed by cells within the infarct zone,^38,114^ it has also been detected in the reactivated ventricular epicardium.^39^ Interestingly, *Sulf1* levels downregulate at 3 to 6 days post-myocardial infarction as the epicardium undergoes EMT to derive the subepicardial mesenchyme.^39^ We speculate that this change in transcription may arise from temporal activation of *Wt1*,^5,115^ which raises questions as to whether the regulatory relationship we describe in development may be recapitulated in cardiac injury.

Overall, our work provides evidence of transcriptional regulation of an HS-modifying enzyme in the developing epicardium to modulate cellular processes critical to heart formation and function. Whether this regulatory relationship additionally impacts paracrine HSPG-dependent signaling in epicardial events following cardiac injury remains to be explored.

## ARTICLE INFORMATION

### Acknowledgments

The authors thank the MRC WIMM Advanced Single Cell OMICS Facility (WASCOF), MRC Weatherall Institute of Molecular Medicine, University of Oxford, for 10X libraries and sequencing; the Dunn School and Jenner Institute Flow Cytometry Facility for Advice and Assistance; and the Biomedical Services Staff for Animal Husbandry. The authors also thank Prof Paul Riley (IDRM, University of Oxford, United Kingdom) for helpful comments that improved the clarity of the article.

### Author Contributions

Conceptualization: A.N. Redpath and N. Smart. Methodology: A.N. Redpath, I.-E. Lupu, and J.M. Vieira. Investigation: A.N. Redpath, I.-E. Lupu, L. Haffreingue, Q.M. Dang, I.R. McCracken, T. Carsana, and J.M. Vieira. Resources: T.H. van Kuppevelt. Formal analysis: A.N. Redpath. Visualization: A.N. Redpath. Writing-original draft: A.N. Redpath. Writing-review and editing: A.N. Redpath, I.-E. Lupu, Q.M. Dang, I.R. McCracken, J.M. Vieira, and N. Smart. Funding acquisition: A.N. Redpath, I.-E. Lupu, J.M. Vieira, and N. Smart. Supervision: A.N. Redpath and N. Smart.

### Disclosures

None.

### Supplemental Material

Supplemental Materials and Methods

Tables S1–S10

Figures S1–S12

Major Resources Tables

ARRIVE Guidelines

Uncropped Western Blots

References 116–126

## Supplementary Material
