# Supplementary material for: Regulated HSPG Signaling Directs Epicardial Behavior to Support Cardiac Formation
Source: Circ Res. 2026 Aug 25;139(8):e327330. doi: 10.1161/CIRCRESAHA.125.327330 (PMC13608351; doi:10.1161/CIRCRESAHA.125.327330)
Supplement: Supplementary file 1 [file res-139-e327330-s001.pdf]

SUPPLEMENTAL MATERIAL

Expanded Materials & Methods

Online Figures S1-S12

Online Tables S9-S10

Major Resources Tables

ARRIVE Guidelines

References [116-126]

Regulated HSPG signaling directs epicardial behavior to support cardiac formation

Andia N. Redpath[1,*], Irina-Elena Lupu[1], Louis Haffreingue[1], Quang M. Dang[1,2], Ian R. McCracken[1], Tamara Carsana[1], Toin H. van Kuppevelt[3], Joaquim Miguel Vieira[4], and Nicola Smart[1,*]

[1]Institute of Developmental and Regenerative Medicine, Department of Physiology, Anatomy & Genetics, University of Oxford, Oxford OX3 7TY, UK.

[2]High-Tech Center, Vinmec International Healthcare System, Hanoi, Vietnam.

[3]Department of Biochemistry, Radboud University Medical Center, Nijmegen, The Netherlands.

[4]School of Cardiovascular & Metabolic Medicine & Sciences, King's College London, London SE5 9NU, UK.

*Correspondence: andia.redpath@dpag.ox.ac.uk and nicola.smart@dpag.ox.ac.uk

Expanded Materials & Methods

Animal models

All procedures were approved by the University of Oxford Animal Welfare and Ethical Review Board, in accordance with Animals (Scientific Procedures) Act 1986 (Home Office, UK). Mice were maintained in individually ventilated cages (IVCs) and ventilated racks at 22 °C and 55% humidity. Mouse strains used include C57BL/6J (Charles River), Gt(ROSA)26Sor$^{tm14(CAG-tdTomato)Hze}$/J (MGI ID: 3809524) or Gt(ROSA)26Sor$^{tm9(CAG-tdTomato)Hze}$/J (MGI ID: 3809523)(Rosa26^{tdTomato}), Wt1$^{tm2(cre/ERT2)Wtp}$/J (MGI ID: 3801682)(Wt1^{CreERT2}), and Sulf1$^{tm1c(KOMP)Mbp}$. Rosa26^{tdTomato}; Wt1$^{CreERT2/+}$ males were bred with Rosa26^{tdTomato}; Wt1$^{CreERT2/+}$ females to generate *Wt1* knock-out embryos (homozygous Wt1^{CreERT2}). Sulf1$^{tm1a(KOMP)Mbp}$ embryonic stem cell clones (MGI ID: 5141826, MMRRC: 063141-UCD, Parental Cell Line JM8A3.N1, ES Cell Clones: DEPD00568_2_A12; DEPD00568_2_H12) were obtained from the KOMP Repository at University of California, Davis. Embryonic stem cells were injected into blastocysts, and resulting chimeras bred to achieve germline transmission of the Sulf1$^{tm1a(KOMP)Mbp}$ allele, before crossing with flippase recombinase-expressing mice Tg(ACTFLPe)9205Dym (MGI ID: 2448985) to remove the SA/LacZ-pA/neo cassette flanked by FRT sites integrated into intron 5 of *Sulf1*. The generated Sulf1$^{tm1c(KOMP)Mbp}$ "floxed" allele (Sulf1$^{fl/fl}$) mice, where critical exons 6 and 7 of *Sulf1* are flanked by loxP sites, were backcrossed to a C57BL/6 background. Conditional deletion of exons 6 and 7 was expected to generate a mis-sense mutation and lead to a premature stop codon in exon 8, resulting in nonsense-mediated decay of the mutant mRNA and loss of protein expression. Sulf1$^{fl/fl}$ mice were bred to carry Rosa26^{tdTomato}; Wt1^{CreERT2} for inducible, tissue-specific lineage tracing and knock-out following tamoxifen administration (*Sulf1* iKO; deletion allele Sulf1$^{tm1d(KOMP)Mbp}$). For embryo collection, mice were paired overnight, and females were checked the next morning for the presence of a vaginal plug. Pregnant females were administered 100 mg/kg body tamoxifen by oral gavage at E9.5, unless stated otherwise.

Epicardial explant culture and siRNA transfection

Embryonic hearts were dissected into atrial and ventricular pieces and cultured on 1% bovine gelatine-coated chamber slides or 13mm round glass coverslips, in separate wells, in culture medium (15% FBS, 1% Penicillin/Streptomycin, DMEM high glucose GlutaMAX, Gibco, 61965059). All epicardial explants were cultured at 37°C in 5% CO_2. On day 1, explants were inspected for adherence to the coated surface and successful epicardial outgrowth. For *Wt1* KO (Wt1$^{CreERT2/ CreERT2}$) and control (Wt1$^{+/+}$ or Wt1$^{CreERT2/+}$) explants, fresh medium was added, and cultures were maintained for a total of 3 days. For epicardial explants from C57BL/6J wild-

type hearts to be subjected to the siRNA transfection, fresh medium was provided, and siRNA and Lipofectamine RNAiMAX transfection reagents (ThermoFisher, 13778075) were prepared according to the manufacturer's instructions. Explants were transfected with 60nM Silencer Select Negative Control (Life Technologies, 4390843) or 60nM Silencer Select Wt1 siRNA (Life Technologies, s76112) for 48 hours in culture medium (10% FBS, 1% Penicillin/Streptomycin, DMEM high glucose GlutaMAX). Finally, all explants were washed with PBS, fixed in 4% paraformaldehyde (PFA, Santa Cruz, sc-281692) for 15 minutes at room temperature, and stored as needed for downstream staining. Fixed explants were stored at 4°C in PBS prior to immunostaining. For RNAscope RNA fluorescence in situ hybridization (RNA FISH), fixed explants were dehydrated through a graded ethanol series (50%, 70%, and 100%) and stored at -20°C in 100% ethanol until further processing.

Epicardial cell line culture and siRNA/pDNA transfection

MEC1, an epicardium-derived cell line [58] (Sigma-Aldrich, SCC187), was cultured on 0.1% bovine gelatine (Sigma-Aldrich, G1393) in MEC1 expansion medium (10% EmbryoMax ES Cell Qualified FBS ES009-M, 1% Penicillin/Streptomycin, EmbryoMax DMEM high glucose SLM-120) at 37°C in 5% CO_2. For all experiments using MEC1 cells, passages between 20 and 35 were typically used, with no more than 15 passages of continuous culture. MEC1 cells were seeded overnight on 1% bovine gelatine-coated, assay-specific, tissue culture treated plates in MEC1 expansion medium, before being subjected to siRNA or plasmid DNA (pDNA) transfection procedures.

For siRNA transfection, cells were provided with low serum medium (2% EmbryoMax ES Cell Qualified FBS, 1% Penicillin/Streptomycin, EmbryoMax DMEM high glucose), while siRNA and Lipofectamine RNAiMAX transfection reagents (ThermoFisher) were prepared according to the manufacturer's instructions. MEC1 cells were transfected with 60nM Silencer Select Negative Control, 60nM Silencer Select Wt1 or 40nM Silencer Select Sulf1 siRNAs (a 1:1 combination of s109342 and s109341; Life Technologies) for 24, 48, 72 or 96 hours in low serum medium at 37°C in 5% CO_2.

For pDNA transfection, cells were provided with serum- and antibiotic-free medium (Opti-MEM, Gibco, 31985070), while pDNA, P3000 enhancer and Lipofectamine 3000 transfection reagents (ThermoFisher, L3000001) were prepared as per the manufacturer's instructions. MEC1 cells were transfected with custom vectors, 100ng pRP[En]-{Sulf1_Enh_12715347_5982_WT_SL}: miniCMV>Luciferase (VB230713-1205ffr) or 100ng pRP[En]-{Sulf1_Enh_12715347_WT1_MUT5_SL}: miniCMV>Luciferase (VB230713-1207huq) (VectorBuilder) and incubated at 37°C in 5% CO_2 for 4 hours in serum- and antibiotic-free Opti-MEM medium (1µg pDNA/ml medium). After 4 hours, pDNA transfection

medium was replaced with MEC1 expansion medium, and cells were incubated for 24 or 48 hours at 37°C in 5% CO_2.

Tissue collection and cryosectioning

Embryos and embryonic hearts were harvested and fixed in 4% PFA for 1.5-2 hours at room temperature. After PBS washes, tissues were equilibrated overnight at 4°C in 30% sucrose/PBS and gradually transitioned into O.C.T. embedding medium. Samples were stored at -80°C before being cryosectioned at 8-12µm thickness.

RNAscope RNA fluorescence in situ hybridization (RNA FISH)

RNAscope Multiplex Fluorescent v2 assay (Bio-Techne, 323110) was performed on 8-12 µm cryosections or epicardial explants according to manufacturer's instructions, with minor modifications [2]. Briefly, for cryosections, the assay was conducted according to manufacturer's instructions, with the following adjustments: the target retrieval step which was reduced to 10 minutes, and the Protease Plus digestion which was optimised for the embryonic stage of the tissue (i.e. E9.5-E11.5: 10 minutes; E13.5: 15 minutes; E17.5: 20 minutes) at room temperature. For epicardial explants, samples were rehydrated through a graded ethanol series (70%, 50%) into PBS, followed by digestion with protease III (1:15, in PBS) at room temperature for 10 minutes. The RNAscope assay was then performed according to manufacturer's instructions. All catalogue probes, including the negative control, and TSA Plus fluorophores are listed in the Major Resources Table. Where required, RNA FISH was followed by immunostaining. Samples were blocked in a buffer containing 1% BSA and 10% donkey serum in PBS for 15 minutes at room temperature. Primary antibodies were added, and samples were incubated either for 1 hour at room temperature (for conjugated antibodies; cTNT, BD Biosciences, 565744, 1:100) or overnight at 4°C (for unconjugated antibodies; SMMHC, Abcam, ab125884, 1:200). After PBS washes, when required, secondary antibodies were added and incubated for 1 hour at room temperature, followed by additional PBS washes. Nuclei were stained with DAPI. RNAscope 3-plex Negative Control Probe and no-primary-antibody controls (secondary antibody only) were included to assess probe or antibody targeting, and to guide microscopy exposure and gain settings for tissue/cell autofluorescence and background signal. Samples were mounted in Prolong Gold Antifade Mountant (ThermoFisher, P36930) with glass coverslips, then stored at 4°C until imaging. Fluorescence images of cryosections were acquired using a Leica DM6000B microscope, while confocal fluorescence images were captured with a Zeiss LSM 880 microscope. Image processing and analysis were performed using ImageJ/Fiji and CellProfiler software. Quantification was performed using custom CellProfiler pipelines, adapted from a previously published method [116]. Briefly, nuclei were segmented by intensity thresholding of the DAPI

channel, and cell boundaries were subsequently defined by propagation from the segmented nuclei. Additional intensity thresholds and filtering criteria were applied to identify and quantify RNAscope signal puncta (dots) on a per-cell basis. Additional filtering based on marker expression (e.g., tdTomato positive) was used to define the population of interest. The analysis pipeline quantified both the mean number of dots per cell and the percentage of cells within predefined expression bins for each target gene and image (ROI). Quantitative measurements obtained from multiple small or large ROIs were subsequently averaged to generate representative mean values for each biological replicate.

Immunofluorescent staining

Cryosections were rehydrated and permeabilized with 0.5% Triton X-100 in PBS for 15 minutes. Samples were blocked in staining buffer containing 4% FBS and 10% donkey serum in 0.2% Triton X-100/PBS for 1 hour at room temperature. Primary antibodies listed in the Major Resources Table were added, and samples were incubated overnight at 4°C. Following incubation, samples were washed in 0.1% Triton X-100 in PBS (PBST). Secondary antibodies were then added and incubated for 1 hour at room temperature, followed by additional PBST washes. Nuclei were stained with DAPI. Cultured epicardial explants and MEC1 cells followed the same immunofluorescence protocol, omitting the initial permeabilization step. No-primary-antibody controls (secondary antibody only) were included to assess antibody targeting, and to guide microscopy exposure and gain settings for tissue/cell autofluorescence and background signal. Fluorescence images were acquired using a Leica DM6000B fluorescence microscope for cryosections, and a Molecular Devices ImageXpress Pico Automated Cell Imaging System for stained MEC1 cells in PhenoPlate™ 96-well microplates. Image processing and analysis were performed using ImageJ/Fiji, CellReporterXpress, or CellProfiler software. Quantification was completed using CellReporterXpress or a custom CellProfiler pipeline.

RNA extraction and quantitative RT-PCR

RNA was extracted from embryonic hearts using RNeasy Mini kit (Qiagen, 74104) and from cultured MEC1 cells using RNeasy Plus Micro kit (Qiagen, 74034), following the manufacturer's instructions. RNA concentrations were measured using BMG Labtech Spectrostar Nano absorbance microplate reader equipped with an LVis Plate. cDNA was synthesized with High-Capacity cDNA Reverse Transcription kit (ThermoFisher, 4368814) according to the manufacturer's instructions and analysed by quantitative RT-PCR using the Fast SYBR Green Master Mix (ThermoFisher, 4385616). Primer sequences are detailed in Major Resources Table. The delta-delta cycle threshold (ΔΔCT) method was used for relative

quantification. qRT-PCR program used: Stage 1: Hold: 95 °C for 20 sec; Stage 2: PCR, Denaturation: 95 °C for 1 sec, Annealing: 63 °C for 20 sec. Total of 40 cycles; Stage 3: Melt curve: 95°C for 15 sec, 63°C for 1 min, 95°C for 15 sec. For embryonic hearts ΔΔCT values were calculated, normalised to the mean of the housekeeping genes *Gapdh* and *Hprt1,* and referenced to E11.5 samples. For MEC1 cells ΔΔCT values were calculated, normalised to the mean of the housekeeping genes *Gapdh* and *Actb,* and referenced to control siRNA samples at each time point. For *Sulf1* transcript variant quantification, primer amplification efficiency was determined by standard curve analysis. qRT-PCR program used: Stage 1: Hold: 95 °C for 20 sec; Stage 2: PCR, Denaturation: 95 °C for 1 sec, Annealing: 60 °C for 20 sec. Total of 40 cycles; Stage 3: Melt curve: 95°C for 1 sec, 60°C for 20 sec, 95°C for 1 sec. ΔCt values were calculated by normalisation to the housekeeping gene *Actb*, and expression levels were calculated relative to the reference gene *Upk3b* using the efficiency-corrected Pfaffl method.

Heart tissue dissociation and cell isolation

Whole embryonic hearts were enzymatically dissociated using the Neonatal Heart Dissociation Kit (Miltenyi Biotec, 130-098-373) and the gentleMACS Octo Dissociator with heaters (Miltenyi Biotec, 130-096-427) following the manufacturer's instructions and as previously described [117]. Where necessary, embryonic hearts were pooled prior to dissociation. The dissociation yielded single-cell suspensions for downstream applications, including PrimeFlow RNA Assay, FACS and 10X Genomics. The total number of cells per sample was determined using a hemocytometer and 0.4% trypan blue, with cell viability estimated to be >94%.

PrimeFlow RNA Assay

E17.5 control hearts (Rosa26^{tdTomato}; Wt1$^{CreERT2/+}$) from littermate embryos were pooled prior to dissociation (2-3 hearts/sample) to maximise cell numbers for multiple PrimeFlow RNA staining combinations and corresponding fluorescence minus one (FMO) controls. *Sulf1* iKO hearts (Rosa26^{tdTomato}; Wt1$^{CreERT2/+}$; Sulf1$^{fl/fl}$) were dissociated individually. Single-cell suspensions were processed using the PrimeFlow RNA Assay Kit (ThermoFisher, 88-18005-210) according to the manufacturer's instructions, with minor modifications [117]. Briefly, cells were surface-stained with the Zombie Aqua Fixable Viability Kit (Biolegend, 423101, 1:1000) for 30 minutes at room temperature. Samples in cell staining buffer (Biolegend, 420201) were pre-treated on ice for 5 minutes with TruStain FcX antibody to block Fcγ receptors (Biolegend, 101319, 1:50), followed by incubation with BV605 anti-CD31 antibody (Biolegend, 102427, 1:50) for 30 minutes on ice. Samples were further fixed and permeabilised using the kit reagents. Intracellular staining was performed using BV421 anti-cardiac troponin T (cTNT)

antibody (BD Biosciences, 565618, 1:200) and/or Alexa Fluor 488 anti-Wilms tumor antibody (Abcam, ab202635, 1:100) for 30 minutes on ice, followed by additional fixation, RNA target probe hybridization, and signal amplification per the manufacturer's instructions. After signal amplification and washes, samples were stored in IC fixation buffer (1:1, cell suspension:IC fixation buffer) at 2°C-8°C, and data were acquired within 3 days. PrimeFlow catalogue probes used are detailed in the Major Resources Table. PrimeFlow RNA negative controls (dapB probe) were used to assess probe targeting (upon initial validation), and fluorescence minus one (FMO) controls were included in each experiment to guide population gating. All samples were analysed using BD LSRFortessa X-20 cytometer equipped with a High Throughput Sampler (HTS), and data were processed with FlowJo software. Percent positive was calculated using Super-Enhanced Dmax Subtraction algorithm (%SED; FlowJo) for histogram comparisons. Geometric mean fluorescence intensity (gMFI) was calculated by subtracting the FMO gMFI from sample gMFI.

Fluorescence-activated cell sorting (FACS)

E12.5 or E13.5 hearts ($Rosa26^{tdTomato}$; $Wt1^{CreERT2/+}$) from littermate embryos were pooled prior to dissociation (E12.5: 7 hearts/sample; E13.5: 9-13 hearts/sample) to maximise cell numbers for FACS and corresponding FMO controls, and achieve a target sort of 50,000 epicardial cells/sample for the downstream ATAC-seq protocol. Single-cell suspensions in cell staining buffer (Biolegend) were pre-treated for 5 minutes on ice with TruStain FcX antibody, followed by surface staining with BV421 anti-CD31 (Biolegend, 102424, 1:50) and APC/Cy7 anti-Podoplanin (PDPN; Biolegend, 127418, 1:100) antibody for 30 minutes on ice, then washed with PBS before being incubated with SYTOX Green Dead Cell Stain (ThermoFisher Scientific, S34860, 1:50,000) for 5 minutes on ice. After PBS washes, cells were resuspended in sorting buffer containing 2% FBS in PBS. BD FACSAria III was used to sort 40,000-50,000 live cells negative for SYTOX Green and CD31, and positive for tdTomato and PDPN, into 0.5% BSA in PBS.

ATAC-seq and Analysis

Live epicardial cells ($CD31^{-}tdTomato^{+}PDPN^{+}$) isolated by FACS were subjected to the ATAC-seq protocol as previously described [118] (dx.doi.org/10.17504/protocols.io.bv9mn946). In brief, 40,000-50,000 sorted cells were centrifuged at 500 ×g for 5 minutes, the supernatant was removed, and the pellet was resuspended in 50µL of ATAC Resuspension Buffer (RSB: 10mM Tris-HCl pH 7.5, 10mM NaCl, 3mM $MgCl_2$) supplemented with 0.1% NP-40, 0.1% Tween-20, and 0.01% Digitonin to extract nuclei. After a 3-minute incubation, 1mL of ATAC RSB with 0.1% Tween-20 was added, mixed by inversion, and centrifuged at 500 ×g for 10 minutes. The supernatant was carefully removed, and nuclei were resuspended in 50µL of transposition

mix (100mM tagmentation enzyme, Illumina, 20034197, 0.01% Digitonin, 0.1% Tween-20, 0.33X PBS, in TD buffer). The tagmentation enzyme amount was adjusted according to cell count (for 40,000 cells, the enzyme was diluted to a 0.8× concentration in TD buffer before being added to the transposition mix). Incubation and centrifugation steps were performed on ice or at 4°C, respectively, up to this point. Transposition reactions were carried out at 37°C for 30 minutes with shaking at 1,000 RPM using an Eppendorf ThermoMixer.

ATAC-seq DNA libraries were generated as previously described [118] (dx.doi.org/10.17504/protocols.io.bv9mn946), with minor modifications. Reactions were purified using the MinElute Reaction Cleanup Kit (Qiagen, 28204) following the manufacturer's instructions. Pre-amplification of transposed fragments (5 cycles) was performed using NEBNext Ultra II Q5 Master Mix (NEB, M0544S) and Nextera i7 index adapters (IDT). The number of additional amplification cycles (typically 4) was determined using the qPCR method [119]. Final PCR products were purified using a double-sided bead purification method with SPRIselect beads (0.5X volume followed by 1.3X volume of SPRI beads; Beckman Coulter, B23317). Final clean-up was performed with the MinElute Reaction Cleanup Kit (Qiagen). Libraries were assessed for quality using an Agilent TapeStation with a D1000 DNA ScreenTape assay (Agilent, 5067-5583), and quantified with the KAPA Library Quantification Kit (Roche, 07960336001). Next-generation sequencing (NGS) was conducted on equimolar pooled libraries using the NextSeq 500/550 High Output v2.5 kit (75 cycles) (Illumina, 20024906), with paired-end reads (40 bp x 2) and 8 bp single index reads.

Sequencing data were demultiplexed using the Illumina platform and processed using Samtools, Bowtie2, Picard, Genrich, and deepTools. Nextera adaptor sequences and low-quality reads were trimmed during the bcl2fastq demultiplexing step. Reads were aligned to the mouse genome (mm10) using Bowtie2 with default parameters and a maximum fragment length of 1,000 bp. Peak calling and filtering were performed using Genrich, Samtools, and Picard. Duplicate reads were removed, and remaining reads were filtered for high quality (MAPQ ≥ 30), non-mitochondrial chromosomes, genomic blacklist regions (mm10 exclusion), and properly paired reads. Read coverage was normalized using reads per genome coverage (RPGC) and an effective genome size of 2,308,125,349. Transcription factor occupancy was predicted using TOBIAS [68]. For visualization in the Integrative Genomics Viewer (IGV), the mean signal of two independent E13.5 samples was computed using Galaxy server's bigwigCompare tool, with the operation set to "mean" and a bin size of 1.

scRNA-seq and Analysis

For E17.5 single-cell RNA sequencing (scRNA-seq), a whole embryonic heart was dissociated and processed using the Chromium Single Cell 3' Reagent Kit (v3 chemistry, 10x Genomics, PN-1000092) following the manufacturer's instructions. Libraries were sequenced in three runs on the Illumina NextSeq 500 platform using the NextSeq® 500/550 High Output Kit v2 (150 cycles, Illumina, FC-404-200), generating 400 million reads, with a target of 8,000 cells per sample. Sequencing reads were processed with CellRanger (10x Genomics), and downstream analysis was performed in Seurat v5, as described below. Cells with more than 25% mitochondrial reads, fewer than 1,000 genes, or more than 7,000 genes were excluded from further analysis. Data were visualized using UMAP. The E10.5 scRNA-seq dataset [40] was processed as previously described [2]. The E13.5 and E17.5 scRNA-seq datasets were processed as described in a prior study [41], with additional doublet removal performed using the scDblFinder package (based on doublet density). For annotating tdTomato+ cells, tdTomato expression>1 was used. For batch correction and data integration of E11.5, E13.5, and E17.5 datasets, publicly available E11.5 scRNA-seq of the heart [48] was obtained from GEO (GSE193746). Read counts from datasets (18542 cells in total) were combined and underwent shifted logarithm (log1p) normalisation. Highly variable genes were selected in a batch-aware manner using Scanpy v1.9.5 [120]. Using the top 5000 most highly variable features, datasets were integrated using scGen v2.1.0 [121] with default parameters. Batch-corrected latent representation from scGen was used to calculate a new UMAP embedding. The Leiden algorithm was then used to perform cell clustering using Scanpy. The integrated epicardial cluster was identified by its expression of the markers *Upk3b*, *Upk1b*, and *Wt1*. The epicardial cluster (1093 cells) was subset and re-normalised using global scaling by the total counts per cell. Median values of gene expression were computed using Scanpy. Cell cycle phase scores (S-phase and G2M-phase) for individual cells were assigned using Seurat, and cells with *Mki67* expression levels greater than 0.5 were considered positive in the analysis. Publicly available E17.5 scRNA-seq data from endothelial cells isolated from *BmxCreERT2*;*Rosa*^{tdTomato} lineage traced mouse hearts [52] was obtained from GEO (GSE213274) and processed as previously described [122]. Publicly available E14.5 scRNA-seq data of Wt1$^{GFPCre/GFPCre}$ (*Wt1* knock-out) mouse hearts [46] was obtained from GEO (GSE193346). Seurat differential expression (DE) analysis, based on the non-parametric Wilcoxon rank-sum test with Bonferroni correction for multiple comparisons, was conducted on the epicardial cluster to compare control versus *Wt1* knock-out. Results were visualized in a volcano plot, with significance thresholds set at an adjusted p-value < 0.05 and fold change (FC) > 0.5. Although samples were individually barcoded in this dataset [46], biological replication was limited, comprising one control mouse and two *Wt1* KO mice, with ~99.7% of

Wt1 KO cells contributed by a single biological replicate. Consequently, statistical analyses were restricted to population-level comparisons, with multiple testing correction performed based on the number of features tested.

Ligand-receptor inference analysis was performed on the E13.5 scRNA-seq dataset using CellChat [70]. To enhance the analysis, we customized the ligand-receptor interaction database by updating the CellChatDB v1.6.0 repository with additional ligand-receptor pairs from resources such as CellPhoneDBcore (≤4.1) [123], experimentally validated interactions from scTalkDB [124] and STRING, and included literature-based annotations for HSPG-dependent pathways.

CUT&RUN-seq and Analysis

MEC1 cells cultured for 2 days in expansion medium were harvested from T75 flasks using 0.05% Trypsin-EDTA (2-3 minutes at 37°C). Cell viability was estimated at >95%, and cells were divided based on immunogen ($1x10^6$ cells/reaction). CUT&RUN was performed using the CUT&RUN Assay Kit (Cell Signaling Technologies, 86652), following the manufacturer's instructions with minor modifications. Cells were incubated with activated Concanavalin A magnetic beads for 10 minutes at room temperature. After washes, cell:bead suspensions were incubated overnight at 4°C with anti-WT1 (Abcam, ab89901, 1:50), anti-H3K27ac (CST, 8173, 1:100), anti-H3K4me3 (CST, 9751, 1:50), or anti-IgG (CST, 66362, 1:20) antibodies on a gentle rocker. The next day, pA/G-MNase was added, and cells were incubated at 4°C for 1 hour, followed by $CaCl_2$-mediated MNase activation for DNA digestion (30 minutes at 4°C, Eppendorf ThermoMixer). Reactions were terminated with Stop buffer supplemented with 50 pg spike-in DNA per reaction (*S. cerevisiae* fragmented genomic DNA), and DNA fragments were released by incubation at 37°C for 10 minutes. Input genomic DNA samples ($1x10^5$ cells/reaction) were prepared using DNA extraction buffer (containing Proteinase K and RNase A) according to the manufacturer's protocol. Samples were incubated at 55°C for 1 hour with shaking (500 RPM, Eppendorf ThermoMixer), cooled on ice, then sonicated using a Diagenode Bioruptor Pico set to "Easy Mode" (25 cycles of 30 sec on/off at 4°C). DNA fragments were purified using spin columns (CST, 14209). DNA was quantified using the Qubit dsDNA HS Assay Kit (Life Technologies, Q32851), and size distribution was assessed with the High Sensitivity DNA Kit (Agilent, 5067-4626) using a Bioanalyzer. Qubit concentrations were adjusted for fragments between 50 bp and 750 bp (50 bp–1000 bp for input), calculated by normalising to the percentage of total signal in this region.

DNA libraries were prepared using the NEBNext Ultra II DNA Library Prep Kit (NEB, E7645S), with modifications guided by the protocol "Library Prep for CUT&RUN with NEBNext® Ultra™ II DNA Library Prep Kit for Illumina® (E7645) V.2"

(dx.doi.org/10.17504/protocols.io.bagaibse). Less than 6 ng CUT&RUN DNA was used as input. NEBNext End Prep was performed at 20°C for 30 minutes and 50°C for 60 min. Adaptor concentrations were adjusted to input amounts (e.g., 3 µM NEBNext adaptor for 6 ng CUT&RUN DNA). Ligations were performed at 20°C for 15 min, and USER enzyme was used to cleave the uracil loop (37°C for 15 min). DNA cleanup steps used 1.75X SPRIselect beads for WT1/IgG CUT&RUN and 1.1X for histone modifications (H3K27ac, H3K4me3). To amplify the library, the ligation product was mixed with 2x Ultra II Q5 mix, universal primer and index primers (NEB, E7335S, E7500S, E7710S). PCR amplification was typically 12 - 15 cycles (WT1/IgG: 98°C for 30 sec, 12 - 15 cycles of 98°C for 10 sec, 65°C for 10 sec, final extension at 65°C for 5 min; histone modifications: 13sec annealing/extension). Double size selection (0.8X, 1.2X SPRIselect beads) was used for WT1 and IgG samples, while single size selection (1.1X beads) was applied for histone modification samples. Input genomic DNA samples (50 ng input) followed the standard NEBNext Ultra II DNA Library Prep Kit protocol. Libraries were assessed using a High Sensitivity DNA Kit on a Bioanalyzer (Agilent) and quantified using the KAPA Library Quantification Kit (Roche, 07960336001). Next-generation sequencing (NGS) was conducted on equimolar pooled libraries using the NextSeq 500/550 High Output v2.5 kit (75 cycles) (Illumina), with paired-end reads (42 bp x 2) and 6 bp single index reads.

NGS data were demultiplexed using Illumina bcl2fastq, and processed with the nf-core/cut&run v2 pipeline using TrimGalore, Bowtie2, Samtools, Picard, deepTools, Bedtools, and SEACR. TrimGalore removed adaptor sequences, polyG+ sequences and low-quality reads, and Bowtie2 aligned reads to the mouse genome (mm10) with default parameters. Filtering was conducted with Samtools and Picard to exclude low-quality alignments (MAPQ ≥ 20 for WT1/IgG, MAPQ ≥ 10 for histone modifications), genomic blacklist regions, and duplicate reads. Peaks were called using SEACR, normalizing against IgG controls and scaling background control by a factor of 0.8. Read normalization was performed using Counts Per Million (CPM), and consensus peaks were computed from at least two biological replicates. For IGV visualization, the mean signal of two independent biological replicates was computed using Galaxy server's bigwigCompare tool with the operation set to "mean" and bin size of 1.

Enhancer-promoter (E-P) connections were predicted using the Activity-by-Contact (ABC) model (version 0.2.2) [69]. A custom gene annotation file was compiled to facilitate the analysis by using NCBI RefSeq annotation (build 38.1) of TSS and gene body for the mm10 genome. To infer E-P connections, 501-bp candidate enhancer elements were predicted based on MACS2-called ATAC-seq narrow peaks (using the parameters: -f BEDPE -g mm -q 0.01 --nomodel --shift -73 --extsize 146 --call-summits --cutoff-analysis --keep-dup all). Enhancer activities within the 150000 strongest elements were quantified using a combination of E13.5

epicardial ATAC-seq, H3K27ac CUT&RUN-seq of the MEC1 epicardial cell line, and TPM-normalized E13.5 epicardial gene expression (scRNA-seq). ABC scores were then computed by combining region activity and Hi-C contact frequency estimated by a power-law function of genomic distance. The power-law exponent was set to 0.87. To flag genes that are impervious to the effect of distal enhancers and exclude them from the analysis, a published list of ubiquitously expressed genes across mouse tissues [125] was utilised. E-P pairs were retained if their ABC scores exceeded the threshold of 0.02, corresponding to approximately 70% recall and 60% precision.

RNA-seq and Analysis

MEC1 epicardial cell bulk RNA-seq data deposited on GEO (GSE313583; control replicate 1-3), fastq were processed using the nf-core/rnaseq pipeline (version 3.14.0), executed with Nextflow. Briefly, reads were aligned to the mouse mm10 (UCSC) reference genome using the STAR–Salmon workflow, based on GENCODE vM25 gene annotation. Adapter and quality trimming were performed using TrimGalore, implemented by nf-core/rnaseq, with poly-G trimming enabled (--trim-nextseq 20) to mitigate sequencing chemistry–specific artifacts. Genome alignment was performed using STAR. Duplicate marking, coordinate sorting, and BAM indexing were performed. In parallel, transcript-level quantification was performed using Salmon in alignment-based mode, using the STAR alignments. To assess promoter usage of *Sulf1*, we implemented a strand-aware, splice-filtered analysis focused on 5′ read starts. All analyses were restricted to a genomic window encompassing the *Sulf1* locus (chr1:12,690,000–12,720,000; mm10). Promoter 2 was defined a priori based on genomic inspection at chr1:12,717,837–12,719,310. A widened window (±1 kb) around this region was additionally used to account for RNA-seq fragmentation–induced dispersion of 5′ read starts. For each sample, reads mapping to the *Sulf1* locus were extracted using samtools view (v1.18). We retained only spliced reads (CIGAR strings containing an N) for promoter analysis to reduce contamination from internal exon fragments and degraded RNA. Spliced reads were converted to BED12 format using the bamtobed command in bedtools (v2.31.1). Strand-aware 5′ read start positions were then derived from BED12 coordinates. Because mouse *Sulf1* is annotated on the positive strand, only + strand 5′ read starts were retained for downstream analyses. Each read contributed a single base-pair interval corresponding to its inferred 5′ end. Promoter usage was quantified by counting unique spliced, positive-strand 5′ read starts overlapping the Promoter 2 window using the bedtools intersect command. Counts were reported for both the Promoter 2 definition and the Promoter 1 (canonical) window.

Construction of custom vectors and pDNA purification

The consensus WT1 CUT&RUN peak sequence at the *Sulf1* locus (mm10, chr1:12,715,347-12,715,982, 636 bp, wild-type) was cloned into a mammalian enhancer-testing vector containing a miniCMV promoter and luciferase reporter gene (WT_SL; VectorBuilder Inc.). For loss of functional TF binding site analysis, a mutant sequence (WT1_MUT5_SL) was created, in which the two predicted WT1 binding motifs (JASPAR Wt1_MA1627.1) were mutated by altering five nucleotides each. To verify these mutations and assess their impact, *in silico* motif scanning was performed using MEME Suite tools (MAST for motif scanning, Tomtom for motif comparison), ensuring that WT1 binding was abolished without creating new significant binding sites for other transcription factors expressed in epicardial cells.

Custom vectors, pRP[En]-{Sulf1_Enh_12715347_5982_WT_SL}: miniCMV>Luciferase (VB230713-1205ffr) and pRP[En]-{Sulf1_Enh_12715347_WT1_MUT5_SL}: miniCMV>Luciferase (VB230713-1207huq), were generated by VectorBuilder and supplied as E. coli stocks. Bacteria harbouring plasmid DNA vectors were plated on LB-agar containing ampicillin and incubated overnight at 37°C. Individual colonies were picked and expanded in culture, and plasmid DNA was extracted using the PureLink™ HiPure Plasmid Maxiprep Kit (Invitrogen, K210006). The incorporation of the enhancer insert was confirmed by restriction digest using NheI-HF, Bsu36I, and XhoI (NEB, R3131S, R0524S, R0146S).

Heparan sulfate (HS) staining and analysis

Following siRNA transfection of MEC1 cells (48 hours) in PhenoPlate 96-well, flat, optically clear-bottom, black TC-treated microplates (Revvity), the medium was removed, and cells were washed with PBS and fixed with 4% PFA for 10 minutes at room temperature. Cells were then washed and stored in PBS at 4°C prior to HS staining targeting 2-*O*, *N*- and 6-*O*-sulfated epitopes. Cells were blocked in staining buffer containing 0.1% (w/v) BSA in PBS for 30min at room temperature. VSV-tagged phage-display single-chain variable fragment (ScFv) antibodies, AO4B08 ([60], 1:25) or HS3B8 ([126], 1:25) were added, and samples were incubated for 1 hour at room temperature. After washing with PBS, samples were incubated with a Cy3-conjugated anti-VSV glycoprotein antibody (Sigma-Aldrich, C7706, 1:100) for 1 hour at room temperature, followed by additional PBS washes. Stained cells were then rinsed in 100% ethanol, then washed again in PBS, before nuclei were stained with DAPI. No-primary-antibody controls (secondary antibody only) were included to assess antibody targeting, and to guide microscopy exposure and gain settings for cell autofluorescence and background signal. Fluorescence images were captured using the Molecular Devices ImageXpress Pico Automated Cell Imaging System. Each condition was tested in single wells, with five images per well captured using a 20X objective. Image analysis was conducted using ImageJ/Fiji and

CellProfiler software. Quantification was performed with a custom CellProfiler pipeline. Briefly, nuclei segmentation was achieved through thresholding on DAPI staining intensities. HS staining was detected by thresholding on fluorescence intensity within areas co-localising with cellular regions. For each image, both the total HS-positive area (e.g., AO4B08-positive area) and total intensity were measured. Corrected total cellular fluorescence (CTCF) was calculated as: CTCF = Total Intensity – (Area occupied by cells x Mean Background Intensity). This value was normalized by nuclei count to account for cell density differences, and images containing fewer than 700 nuclei were excluded from analysis.

Cryosections were rehydrated in PBS for 15 minutes. Samples were blocked in staining buffer containing 0.1% (w/v) BSA in PBS for 30 minutes at room temperature. VSV-tagged phage-display single-chain variable fragment (ScFv) antibodies, AO4B08 (1:10) or HS3B8 (1:10), and anti-Podoplanin (ThermoFisher, 14-5381-82, 1:100) or Alexa Fluor 647 anti-cTNT (1:100) antibody were added, and samples were incubated for 1.5 hours at room temperature. After washing with PBS, samples were incubated with a Dylight488-conjugated anti-VSV-G antibody (Rockland, 600-441-386, 1:250) and relevant secondary antibodies for 1.5 hours at room temperature, followed by additional PBS washes. Stained samples were rinsed in 100% ethanol, then washed again in PBS, before nuclei were stained with DAPI. No-primary-antibody controls (secondary antibody only) were included to assess antibody targeting, and to guide microscopy exposure and gain settings for cell autofluorescence and background signal. Samples were mounted in Prolong Gold Antifade Mountant (ThermoFisher) with glass coverslips, then stored at 4°C until imaging. Fluorescence images of cryosections were acquired using a Zeiss LSM 880 microscope. Image processing and analysis were performed using ImageJ/Fiji and CellProfiler software. Quantification was performed using custom CellProfiler pipelines,

Luciferase Activity Assay

MEC1 cells were seeded into 96-well, flat, clear-bottom, white TC-treated microplates and transfected with siRNAs for 24 hours, followed by plasmid DNA (pDNA) transfection (4 hours) and an additional 24 or 48 hours at 37°C in 5% CO_2. Luciferase activity was measured using the Bright-Glo luciferase assay system (Promega, E2610) following the manufacturer's protocol. Briefly, the 96-well plates were removed from the incubator and allowed to equilibrate at room temperature for 5 minutes. Bright-Glo reagent was then added to each well in a 1:1 ratio with the medium (100 µL reagent:100 µL medium). After a 2-minute incubation at room temperature, luminescence was measured using a BMG Labtech CLARIOstar luminometer microplate reader.

Proliferation Assay

Following siRNA transfection of MEC1 cells (48 hours) in PhenoPlate 96-well, flat, optically clear-bottom, black TC-treated microplates (Revvity), the medium was removed, and cells were washed with DMEM. The medium was replaced with EmbryoMax DMEM containing 2% KnockOut Serum Replacement and 1% Penicillin/Streptomycin. Cells were incubated at 37°C in 5% CO_2 for 4-5 hours before adding recombinant proteins at the following concentrations: 10 ng/mL hTGF-β1, 10 ng/mL hTGF-β2, 20 ng/mL (50 ng/mL) hFGF2, 50 ng/mL hPDGF-BB, 100 ng/mL (10 ng/mL) hMidkine, 100 ng/mL (20 ng/mL) hPleiotrophin, 100 ng/mL (500 ng/mL) h/mWnt-5a, 50 ng/mL (100 ng/mL) mVEGF-B, 50 ng/mL hIGF-I, 20 ng/mL hR-Spondin-3, 50 ng/mL (10 ng/mL) hBMP-10, 100 ng/mL (50 ng/mL) hIGF-BP4, and 50 ng/mL h/m/rBMP-2 Additional concentrations tested in brackets. An hour before the 24-hour treatment at 37°C in 5% CO_2 ended, 10 µM EdU (ThermoFisher) was added to the medium, and cells were incubated for 1 hour at 37°C in 5% CO_2. After incubation, cells were washed with PBS, fixed with 4% paraformaldehyde (PFA) for 15 minutes at room temperature, and stored in PBS at 4°C after additional washes. For EdU detection, cells were permeabilized with 0.5% Triton-X-100/PBS for 20 minutes at room temperature, then stained using the Click-iT EdU Alexa Fluor 488 Imaging Kit (5 µM Alexa Fluor azide, ThermoFisher Scientific, C10337), following the manufacturer's protocol. Fluorescence images were captured using the Molecular Devices ImageXpress Pico Automated Cell Imaging System. Each condition was tested in duplicate wells, with one image per well captured using a 4X objective. Image analysis was conducted using ImageJ/Fiji, CellReporterXpress, or CellProfiler software. DAPI staining was used to identify nuclei, and a threshold was set to detect EdU-positive nuclei, quantifying cell proliferation. Quantification was performed with CellReporterXpress or a custom CellProfiler pipeline. Images containing fewer than 100 cells were excluded from analysis.

Epithelial-Mesenchymal Transition Assay

Following siRNA transfection of MEC1 cells (48 hours) in PhenoPlate 96-well, flat, optically clear-bottom, black TC-treated microplates (Revvity), the medium was removed, and cells were washed with DMEM. The medium was replaced with EmbryoMax DMEM containing 2% KnockOut Serum Replacement (Gibco, 10828010) and 1% Penicillin/Streptomycin. Recombinant proteins were added at the following concentrations: 10 ng/mL hTGF-β1, 10 ng/mL hTGF-β2, 20 ng/mL (50 ng/mL) hFGF2, 50 ng/mL hPDGF-BB, 100 ng/mL (10 ng/mL) hMidkine, 100 ng/mL (20 ng/mL) hPleiotrophin, 100 ng/mL (500 ng/mL) h/mWnt-5a, 50 ng/mL (100 ng/mL) mVEGF-B, 50 ng/mL hIGF-I, 20 ng/mL hR-Spondin-3, 50 ng/mL (10 ng/mL) hBMP-10, 100 ng/mL (50 ng/mL) hIGF-BP4, and 50 ng/mL h/m/rBMP-2. Additional concentrations tested in brackets. After a 24-hour incubation at 37°C in 5% CO_2, cells were

washed with PBS and fixed with 4% paraformaldehyde (PFA) for 10 minutes at room temperature. Cells were then washed and stored in PBS at 4°C prior to immunostaining, conducted as outlined in this study. Fluorescence images were captured using the Molecular Devices ImageXpress Pico Automated Cell Imaging System. Each condition was tested in duplicate wells, with five images per well captured using a 20X objective. Image analysis was conducted using ImageJ/Fiji or CellProfiler software. Quantification was performed with a custom CellProfiler pipeline. Briefly, the pipeline classified cells based on signal localisation, structure, texture and fluorescence intensity of β-CATENIN and α-SMA staining, categorising cells into four populations: 1. positive for membrane β-CATENIN and negative for α-SMA filaments (β-cat^{hi} α-SMA^{-}), 2. disrupted membrane β-CATENIN and negative for α-SMA filaments (β-cat^{dis} α-SMA^{-}), 3. negative for membrane β-CATENIN and low or negative for α-SMA filaments (β-cat^{-} α-$SMA^{-/lo}$), 4. disrupted/absent membrane β-CATENIN and positive for α-SMA filaments (β-$cat^{-/dis}$ α-SMA^{hi}). Nuclei and cell segmentation was achieved through thresholding on DAPI and β-CATENIN staining intensities, respectively. Cell body boundaries were estimated using β-CATENIN staining and autofluorescence, and outlined by propagating from the segmented nuclei. The cytoplasm was defined by subtracting the nuclear area from the cell body area. Tubeness and line structure filters were applied to enhance and segment α-SMA filaments. Circularity was measured to identify and exclude artifacts, such as fluorescent antibody precipitates. Additional thresholds were applied to refine classification based on: 1. mean intensity of a-SMA filaments, 2. mean intensity of β-CATENIN at cell boundaries, and 3. average sum texture of β-CATENIN at cell boundaries. Images containing fewer than 100 cells were excluded from analysis.

Western Blot and analysis

Following siRNA transfection of MEC1 cells (48 hours) in 12-well plates, the medium was removed, and cells were washed with DMEM. The medium was replaced with EmbryoMax DMEM containing 2% KnockOut Serum Replacement and 1% Penicillin/Streptomycin. Cells were incubated at 37°C in 5% CO_2 for 4 - 5 hours prior to adding recombinant hTGF-β1 protein at a concentration of 10 ng/mL. Cells were incubated with hTGF-β1 for various time points (15, 30, 60, 90, 120, 150 minutes) at 37°C in 5% CO_2. Following incubation, cells were washed with cold PBS and lysed using RIPA buffer (ThermoFisher, 89901) supplemented with 5mM NEM, 1mM DTT, 0.125U/mL Benzonase, and protease/phosphatase inhibitor cocktails (Sigma, Roche). Protein concentrations were quantified using the Pierce BCA Protein Assay Kit - Reducing Agent Compatible (Thermo Scientific, 23250) and measured using BMG Labtech Spectrostar Nano absorbance microplate reader. Samples were reduced, denatured, and loaded onto NuPAGE 10% Bis-Tris Protein Gels (ThermoFisher Scientific, NP0303BOX). After SDS-PAGE, proteins were transferred to PVDF membranes (Bio-Rad, 1704156). Blots

were blocked in 8% skimmed milk/TBST (TBS + 0.1% Tween 20, Sigma) for 1 hour, washed with TBST, and incubated overnight at 4°C with primary antibodies, anti-pSmad2 (CST, 3108,1:1000), anti-Smad2 (CST, 3103,1:1000) and anti-β-Actin (Abcam, ab8224, 1:2500) diluted in 5% BSA/TBST. The following day, blots were washed and incubated with HRP-conjugated secondary antibodies (diluted 1:2500-1:5000 in 5% skimmed milk/TBST) for 1-2 hours at room temperature. Blots were washed in TBST and developed using Immobilon Crescendo Western HRP substrate, then imaged on a ChemiDoc system. Blots were stripped and re-probed to detect different protein targets. Western blot band intensities were quantified using ImageJ software.

Statistical Analysis

Statistical analyses were performed using GraphPad Prism version 10 (GraphPad Software, USA) and R software. Data are presented graphically as mean ± SEM, with the exception of bulk RNA-seq data, which are presented as mean ± SD. Normality was assessed using the Shapiro-Wilk test and inspection of QQ plots. Equality of variance was assessed using the F test for comparisons between 2 groups and the Brown-Forsythe test for comparisons among ≥3 groups, in combination with inspection of SD values. For datasets with sample sizes <6 or nonnormally distributed data, nonparametric tests were applied: 2-tailed Mann-Whitney *U* tests for single-factor comparisons between 2 groups; Mann-Whitney tests with Holm-Šídák correction for multiple comparisons when independently assessing multiple outcomes between 2 groups; and Friedman tests (paired/within-group comparisons) or Kruskal-Wallis tests followed by Dunn multiple comparisons correction for single-factor comparisons among >2 groups, as appropriate; Aligned Rank Transform (ART) ANOVA was performed, and post hoc pairwise comparisons were computed from the ART-transformed model (multifactor contrasts), with Holm-Šídák or Benjamini-Krieger-Yekutieli false discovery rate (FDR) correction for multiple comparisons for 2-factor experimental designs. For datasets with sample sizes ≥6 and normally distributed data, parametric tests were used: Welch t tests for datasets with unequal variance and/or unequal pooling for single-factor comparisons between 2 groups, with Holm-Šídák correction for multiple comparisons when independently assessing multiple outcomes; 1-way ANOVA with Šídák correction for multiple comparisons, for single-factor comparisons among >2 groups with equal variances; and 2-way ANOVA with Šídák or Benjamini-Krieger-Yekutieli FDR correction for multiple comparisons for 2-factor experimental designs. For log-normally distributed data, as determined by lognormality testing and the nature of the assay readout (eg, luciferase assays), values were log-transformed [log(Y+0.5)] before parametric testing using 1-way ANOVA with Šídák correction for multiple comparisons. For proportional data (eg, Epi:Mes cell phenotype proportions), values were logit-transformed

[logit(Y/100)] before analysis using 2-way ANOVA with Benjamini-Krieger-Yekutieli FDR correction for multiple comparisons. 1-way ANOVA with Fisher's LSD post hoc testing was used when multiple comparison correction was not applicable because comparisons were predefined between specific groups. Fisher's exact tests were used for embryonic outcome analyses with small sample sizes. Correlations between variables were assessed using the Pearson's correlation test. Signalling responses over time were additionally analysed using nonlinear regression curve fitting. Linear regression was applied to the first 30 minutes of the response, while Gaussian and lognormal models were fitted to the complete response dataset. Goodness-of-fit analyses were performed to determine the most appropriate model for each dataset. Best-fit parameters, including amplitude, A parameter, area under the curve (AUC), and peak X and Y values, were assessed to evaluate total signalling output over time across each knockdown condition. Statistical significance was defined as $P<0.05$ or $Q<0.05$, and exact values are indicated in the figures or Supplemental Tables where appropriate. Statistical tests and correction methods used for each analysis are detailed in the corresponding figure legends and Table S9. Results of Shapiro-Wilk tests for normality are detailed in Table S10. No statistical methods were used to predetermine sample size. Sample sizes, including biological replicates and independent experimental replicates, are indicated in the figure legends or directly within the figures. As multiple independent experiments were conducted, correction for multiple testing was applied within each experiment and not across experiments.

Supplemental Figures and Figure Legends

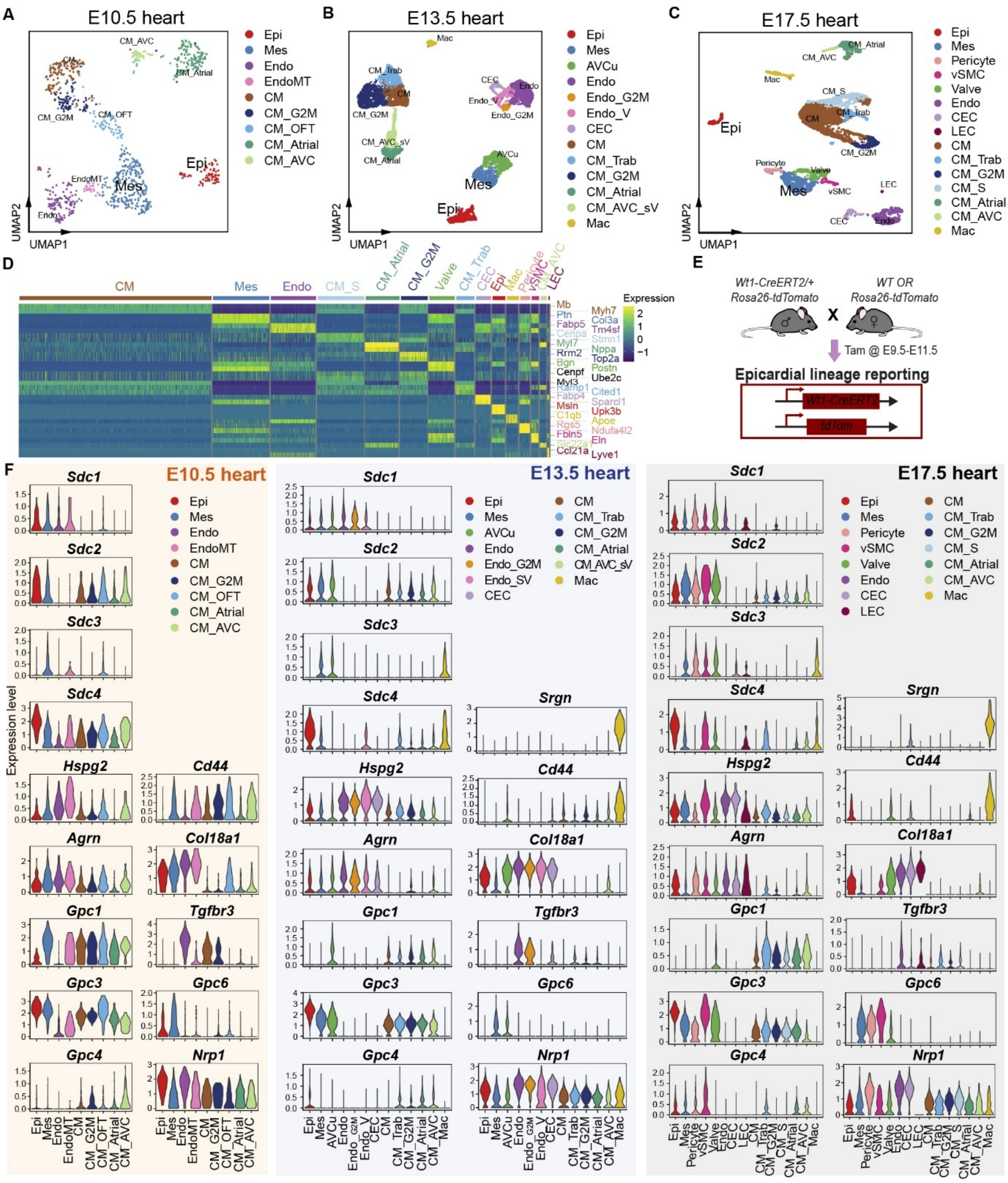

Figure S1 Transcriptional profiling of heparan sulfate proteoglycans in the developing mouse heart

(A) Uniform manifold approximation and projection (UMAP) plot showing the major clusters in E10.5 hearts (total 928 cells, n = 2 batches). (B) UMAP plot showing the major clusters in E13.5 hearts (total of 6,939 cells, n = 3 hearts). (C) UMAP plot showing the major clusters in a E17.5 heart (total of 6,341 cells, n = 1 heart). (D) Heatmap of the top 2 differentially

expressed genes for each cluster in the E17.5 scRNA-seq data. High expression is indicated in yellow. (E) Generation of epicardial-lineage reporting hearts by crossing heterozygous carrier of Wt1-CreERT2 allele and Rosa26-tdTomato allele. Pregnant females induced with tamoxifen at E9.5 - E11.5 to label the newly-formed epicardium (E10.5 - E11.5) and trace its derivatives. (F) Violin plots showing relative expression of 'full-time' and 'part-time' heparan sulfate proteoglycans (HSPG) in the developing mouse heart at E10.5, E13.5 and E17.5.

AVCu, atrioventricular cushion; CEC, coronary endothelial cells; CM, ventricular cardiomyocytes; CM_Atrial, atrial cardiomyocytes; CM_AVC, atrioventricular canal cardiomyocytes; CM_OFT, outflow tract cardiomyocytes; CM_S, S-G2M phase progressing cardiomyocytes; CM_Trab, trabecular cardiomyocytes; Endo, endocardial cells; Endo_V, valvular endothelial and endocardial cells; EndoMT, endocardial-to-mesenchymal transition; Epi, epicardium; G2M, proliferating; LEC, lymphatic endothelial cells; Mac, macrophages; Mes, mesenchyme; Valve, valve mesenchyme; vSMC, vascular smooth muscle cells.

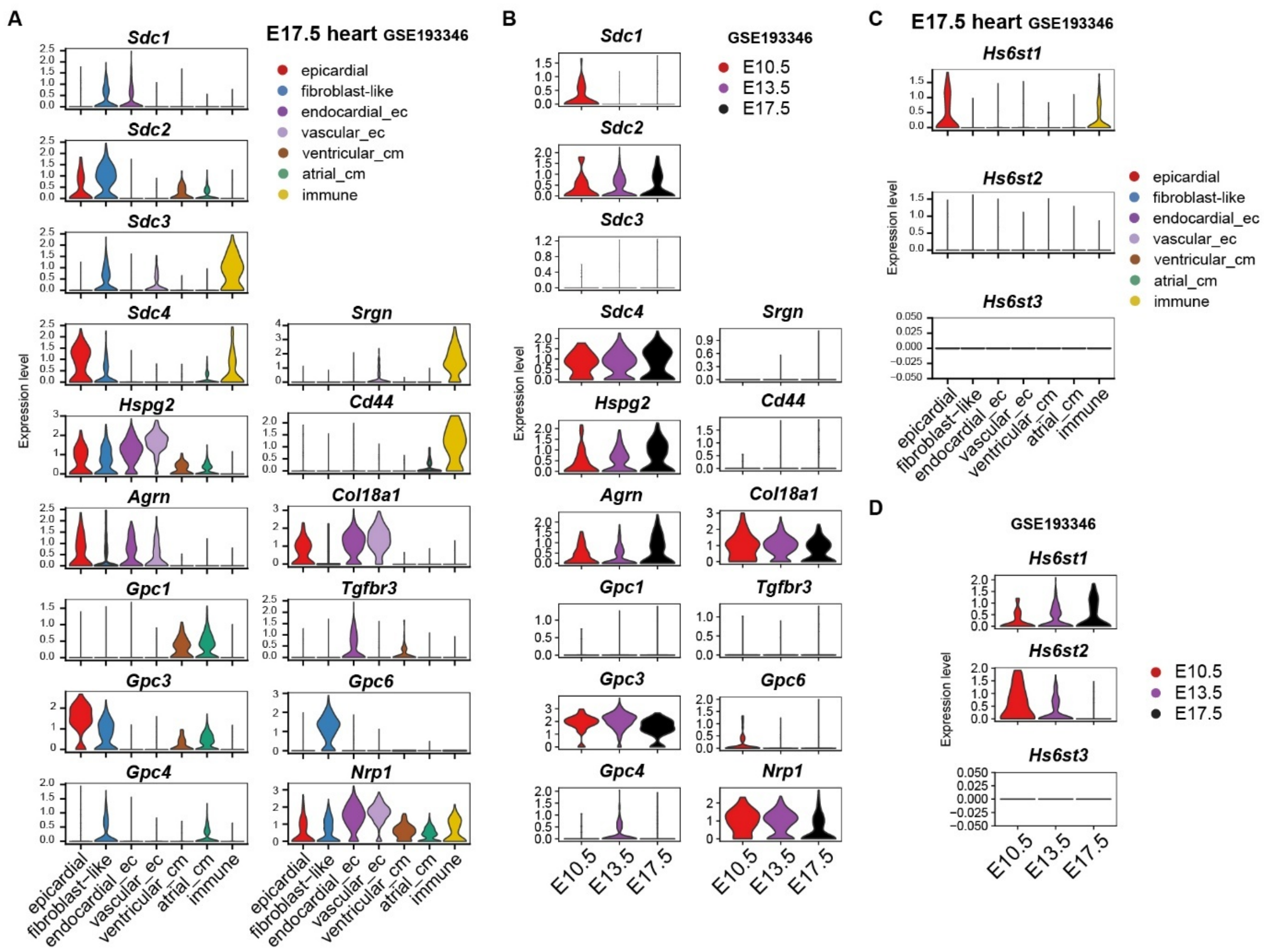

Figure S2 Transcriptional profiling of heparan sulfate proteoglycans and heparan sulfate 6-*O*-sulfotransferases in the developing epicardium

(A) Violin plots showing relative expression of 'full-time' and 'part-time' heparan sulfate proteoglycans (HSPG) in the mouse embryonic heart at E17.5. (B) Violin plots showing relative expression of HSPGs in epicardial cells at E10.5, E13.5 and E17.5. (C) Violin plots showing relative expression of heparan sulfate 6-*O*-sulfotransferases (HS6ST) in the mouse embryonic heart at E17.5. (D) Violin plots showing relative expression of HS6STs in epicardial cells at E10.5, E13.5 and E17.5.

ec, endothelial cells; cm, cardiomyocytes;

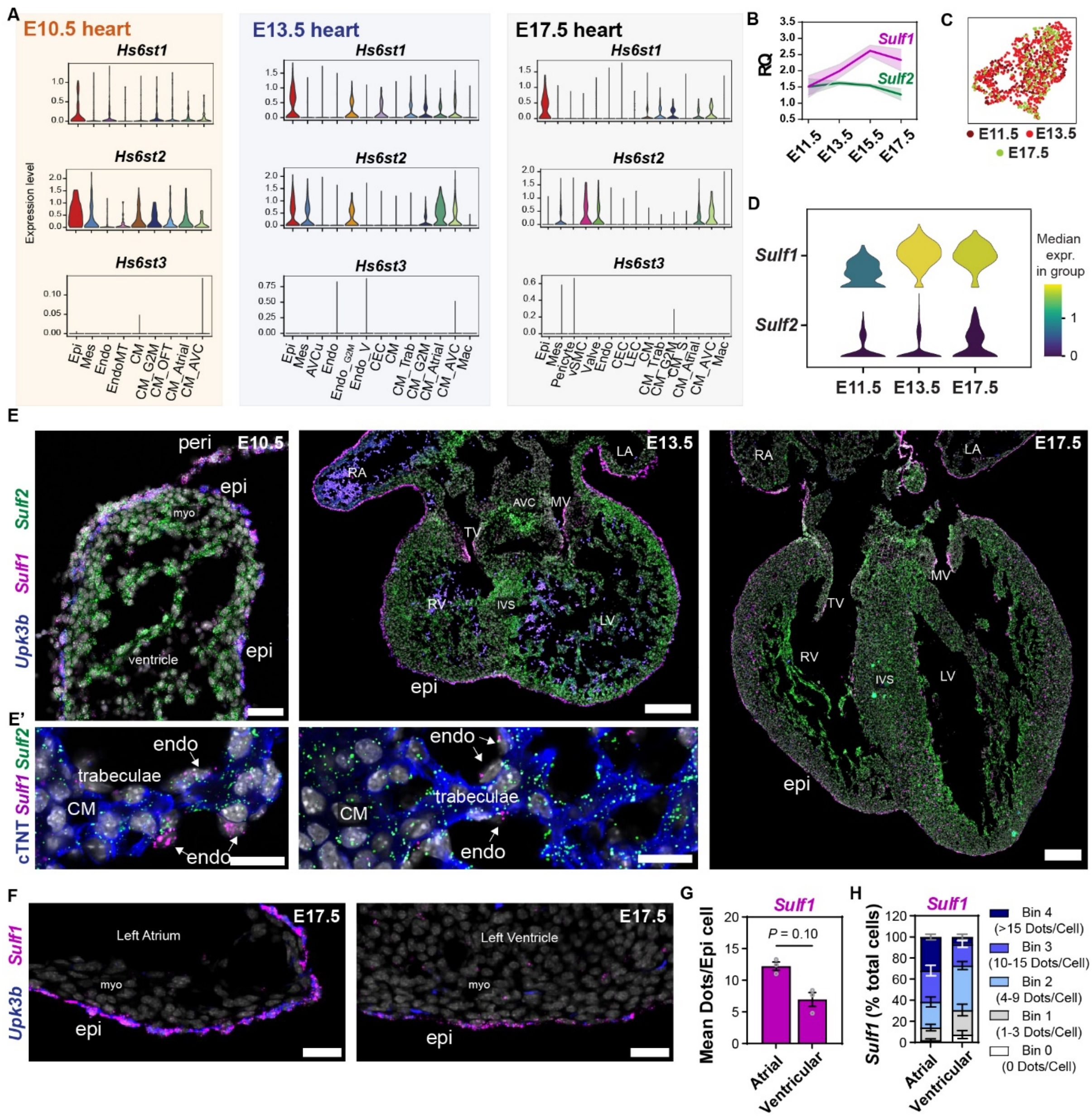

Figure S3 Transcriptional profiling of heparan sulfate 6-*O*-sulfotransferases and 6-*O*-endosulfatases in the developing mouse heart

(A) Violin plots showing relative expression of *Hs6st1*, *Hs6st2* and *Hs6st3* in the developing mouse embryonic heart at E10.5, E13.5 and E17.5. (B) qRT-PCR analysis of *Sulf1* and *Sulf2* transcript expression in whole heart lysates at E11.5, E13.5, E15.5 and E17.5. Values normalized to *Hprt* and *Gapdh*, data are represented as mean ± SEM with shaded error bars (n = 4 hearts). (C) UMAP plot showing integrated epicardial clusters (1093 cells) of E11.5, E13.5 and E17.5 hearts. (D) Stacked violin plot showing the expression of *Sulf1* and *Sulf2* in the epicardial clusters. High median expression is indicated in yellow. (E) Fluorescence ISH on cryosections from E10.5 embryos, E13.5 and E17.5 hearts for *Upk3b*, *Sulf1* and *Sulf2*, showing expression of *Sulf1* in the pericardium, epicardium and derivatives, while widespread

expression of *Sulf2* is detected in the pericardium and myocardium. *Sulf1* is also detected in the forming atrioventricular valves, and early endocardium. Insets show cardiomyocytes expressing *Sulf2*, and endocardial cells expressing *Sulf1* in the trabeculated myocardium. Scale bars: 50 µm (E10.5), 200 µm (E13.5, E17.5) and inset 20 µm. Images representative of n = 7 embryos for E10.5, n = 4 hearts for E13.5, and n = 5 hearts for E17.5. Representative images were chosen according to the following criteria: correct anatomical region, accurate representation of the mean, high quality, and preserved tissue or cellular integrity (F) Fluorescence ISH of E17.5 heart cryosections for *Upk3b* and *Sulf1*, and corresponding quantification (G) of *Sulf1* in atrial and ventricular epicardial cells. Scale bars: 20 µm. Images representative of n = 3 hearts. Representative images were chosen according to the following criteria: correct anatomical region, accurate representation of the mean, high quality, and preserved tissue or cellular integrity. Data are represented as mean ± SEM with individual data points indicated (n = 3 hearts). Mann-Whitney *U* test, with *P* values indicated. (H) Percentage of epicardial cells expressing *Sulf1*, grouped into 5 bins based on the number of dots (individual RNA molecules) per cell and sample size consistent with G.

AVCu, atrioventricular cushion; CEC, coronary endothelial cells; CM, ventricular cardiomyocytes; CM_Atrial, atrial cardiomyocytes; CM_AVC, atrioventricular canal cardiomyocytes; CM_OFT, outflow tract cardiomyocytes; CM_S, S-G2M phase progressing cardiomyocytes; CM_Trab, trabecular cardiomyocytes; Endo, endocardial cells; Endo_V, valvular endothelial and endocardial cells; EndoMT, endocardial-to-mesenchymal transition; Epi, epicardium; G2M, proliferating; LEC, lymphatic endothelial cells; Mac, macrophages; Mes, mesenchyme; Valve, valve mesenchyme; vSMC, vascular smooth muscle cells, peri, pericardium; epi, epicardium; myo, myocardium; RA, right atrium; RV, right ventricle; IVS, interventricular septum; LA, left atrium; LV, left ventricle; AVC, atrioventricular canal; TV, tricuspid valves; MV, mitral valves.

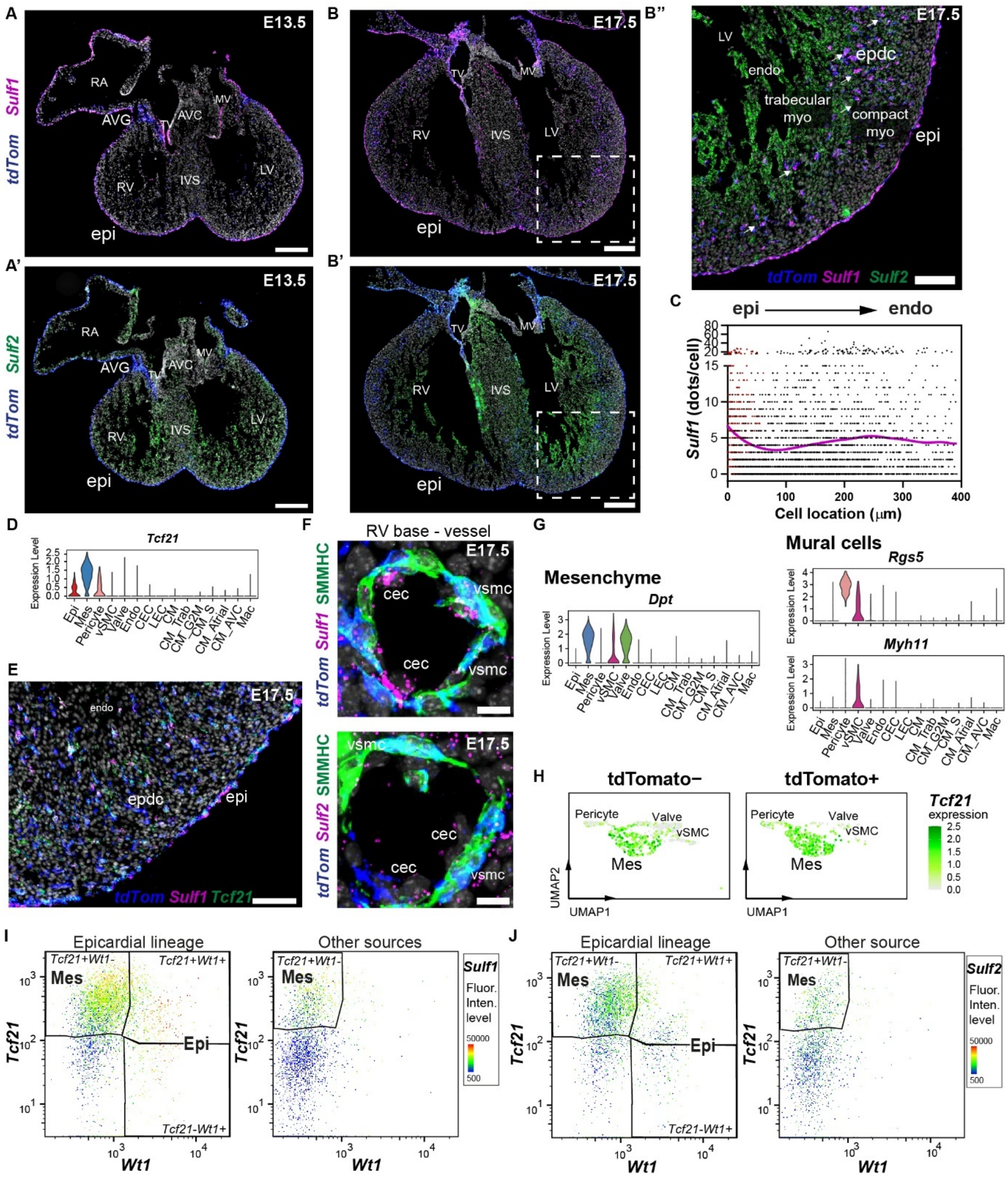

Figure S4 6-*O*-endosulfatases are distinctively expressed in cardiac mesenchyme compartments

(A) Fluorescence ISH of E13.5 heart cryosections for *tdTomato*, *Sulf1* and *Sulf2*, showing majority expression of *Sulf1* in the epicardium, and atrioventricular valves. Scale bars: 250 µm. Images representative of n = 5 hearts. Representative images were chosen according to the following criteria: correct anatomical region, accurate representation of the mean, high quality, and preserved tissue or cellular integrity. (B) Fluorescence ISH of E17.5 heart cryosections for *tdTomato*, *Sulf1* and *Sulf2* showing expression of *Sulf1* and *Sulf2* in epicardium-derived mesenchymal cells invading the myocardium. (B’’) *Sulf1* demonstrates a

trend, from high expression in the epicardium, to low expression in EPDCs located in the compact myocardium, and high expression in EPDCs located proximal to the trabecular myocardium. *Sulf2* demonstrates a gradient, from high expression in the trabecular myocardium to low expression in the compact myocardium. Scale bars: 250 µm and inset 100 µm. Images representative of n = 5 hearts. Representative images were chosen according to the following criteria: correct anatomical region, accurate representation of the mean, high quality, and preserved tissue or cellular integrity. (C) Scatter plot showing quantification of *Sulf1* expression in ventricular epicardial and derivative *tdTomato* positive cells (using Fluorescence ISH images; ROI apex and mid). Data of individual cells from n = 4 hearts, and two ROI images per heart. Cell location is indicated by the Y center coordinate within each ROI image: values of *y*<100µm predominantly correspond to cells in the epicardium and subepicardium, while higher *y* values reflect increasing depth into the compact and trabecular myocardium. A magenta LOWESS smoothing line models *Sulf1* expression in epicardium-lineage cells along their spatial distribution from the epicardium toward the endocardium. Epicardial cells are shown in red. (D) Violin plot showing relative expression of epicardial and mesenchymal marker *Tcf21* in E17.5 hearts. (E) Fluorescence ISH of E17.5 heart cryosections for *tdTomato, Sulf1* and *Tcf21*, showing expression of *Sulf1* in epicardium-derived mesenchymal cells. Scale bars: 100 µm. Images representative of n = 2 hearts. (F) Fluorescence ISH of E17.5 heart cryosections for *tdTomato*, *Sulf1*, *Sulf2* and SMMHC, showing low expression of *Sulf1* and *Sulf2* in epicardium-derived smooth muscle cells. Scale bars: 10 µm. Images representative of n = 2 hearts. (G) Violin plots showing relative expression of markers used to distinguish mesenchyme and mural cells in E17.5 hearts. (H) UMAP feature plot showing expression of *Tcf21* in E17.5 mesenchyme, showing that *Tcf21* is expressed in mesenchymal cells originating from epicardium (tdTomato+) and other sources (tdTomato-). (I-J) FC analysis of E17.5 hearts, populations separated as tdTomato+ epicardial lineage (Epi Lin) or tdTomato- non-epicardial lineage (Other). Colour mapping displays levels of (I) *Sulf1* and (J) *Sulf2*. n=3; pooled 2 hearts per replicate. Gated epicardial lineage as CD31-cTnT-tdTomato+ and non-epicardial as CD31-cTnT-tdTomato-, with subsequent gating of mesenchymal cells (*Tcf21*+*Wt1*-/low) and epicardial cells (*Tcf21*+*Wt1*+ and *Tcf21*-/low*Wt1*+). Colour map highest expression in red.

CEC, coronary endothelial cells; CM, ventricular cardiomyocytes; CM_Atrial, atrial cardiomyocytes; CM_AVC, atrioventricular canal cardiomyocytes; CM_S, S-G2M phase progressing cardiomyocytes; CM_Trab, trabecular cardiomyocytes; Endo, endocardial cells; Epi, epicardium; G2M, proliferating; LEC, lymphatic endothelial cells; Mac, macrophages; Mes, mesenchyme; Valve, valve mesenchyme; vSMC, vascular smooth muscle cells; epi, epicardium; myo, myocardium; RA, right atrium; RV, right ventricle; IVS, interventricular septum; LV, left ventricle; AVC, atrioventricular canal; AVG, atrioventricular groove; TV, tricuspid valves; MV, mitral valves; epdc, epicardium-derived cells.

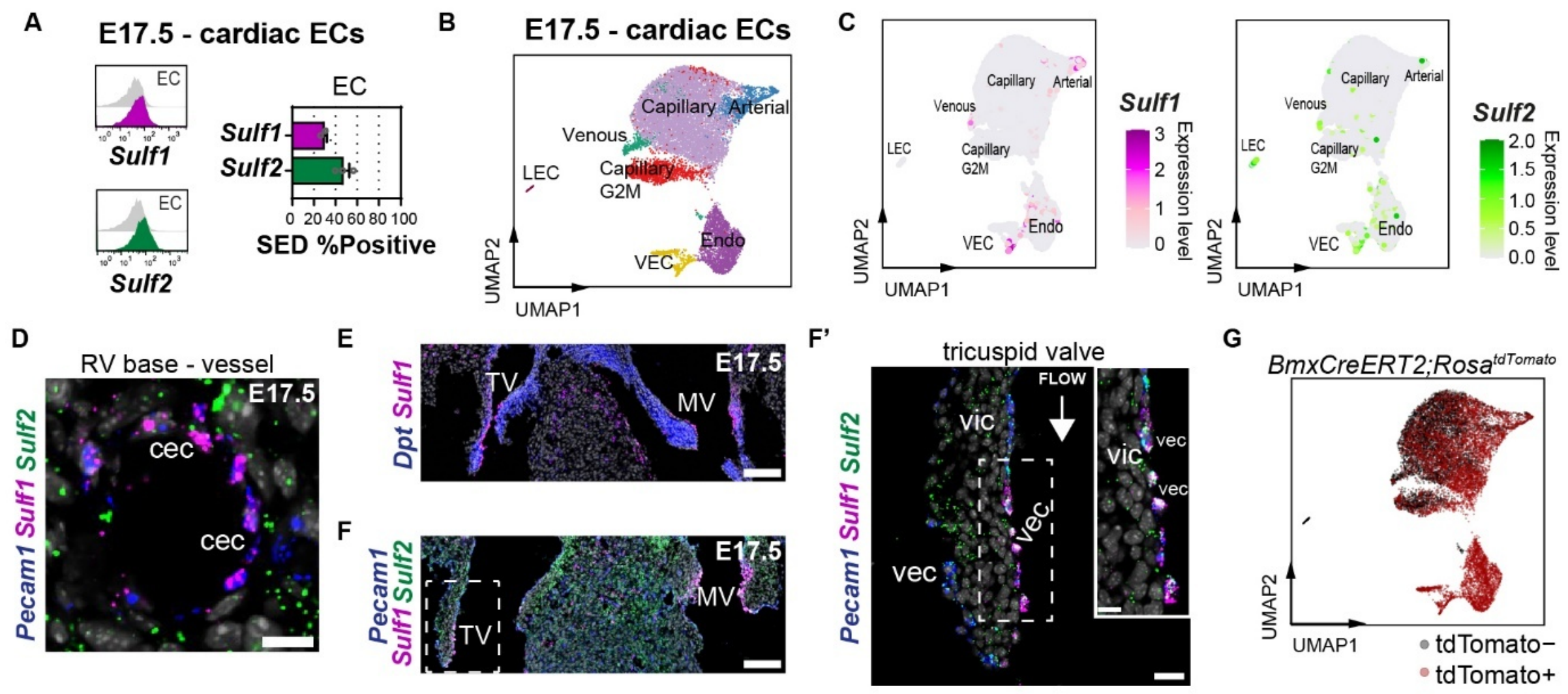

Figure S5 6-*O*-endosulfatases are differentially expressed in cardiac endothelial cell compartments

(A) Flow cytometry analysis of E17.5 endothelial cells in the heart, and corresponding quantification of percentage positive for *Sulf1* and *Sulf2*. Endothelial cells were selected by gating CD31$^+$ cells. Fluorescence minus one (FMO) control shown (grey). Data are represented as mean ± SEM (pooled 2 hearts per replicate; n = 3). (B) UMAP plot showing the major clusters in E17.5 endothelial cells from *BmxCreERT2*;*Rosa^{tdTomato}* hearts. (C) UMAP feature plot showing expression of *Sulf1* and *Sulf2*, in individual endothelial cells of E17.5 *BmxCreERT2*;*Rosa^{tdTomato}* hearts. (D) Fluorescence ISH of E17.5 heart cryosections for *Sulf1*, *Sulf2* and *Pecam1*, showing expression of *Sulf1* enriched in coronary endothelial cells of macrovessels. Scale bars: 10 µm. Images representative of n = 3 hearts. (E-F) Fluorescence ISH of E17.5 heart cryosections for *Sulf1, Dpt*, *Sulf2* and *Pecam1*, showing co-expression of *Sulf1 and Sulf2* in atrioventricular valve endothelial cells. (F') White arrow represents blood flow. Scale bars: 20 µm and inset 10 µm. Images representative of n = 3 hearts. Representative images were chosen according to the following criteria: correct anatomical region, accurate representation of the mean, high quality, and preserved tissue or cellular integrity (D through F'). (G) UMAP plot showing *tdTomato* positive and negative endothelial cells in the E17.5 *BmxCreERT2*;*Rosa^{tdTomato}* hearts. *tdTomato* positive ECs mark the endocardial lineage achieved through temporal labelling (Tamoxifen induction at E9.5).

EC, endothelial cells; Endo, endocardial cells; LEC, lymphatic endothelial cells; cec, coronary endothelial cells; TV, tricuspid valves; MV, mitral valves; vec, valve endothelial cells; vic, valve interstitial cells.

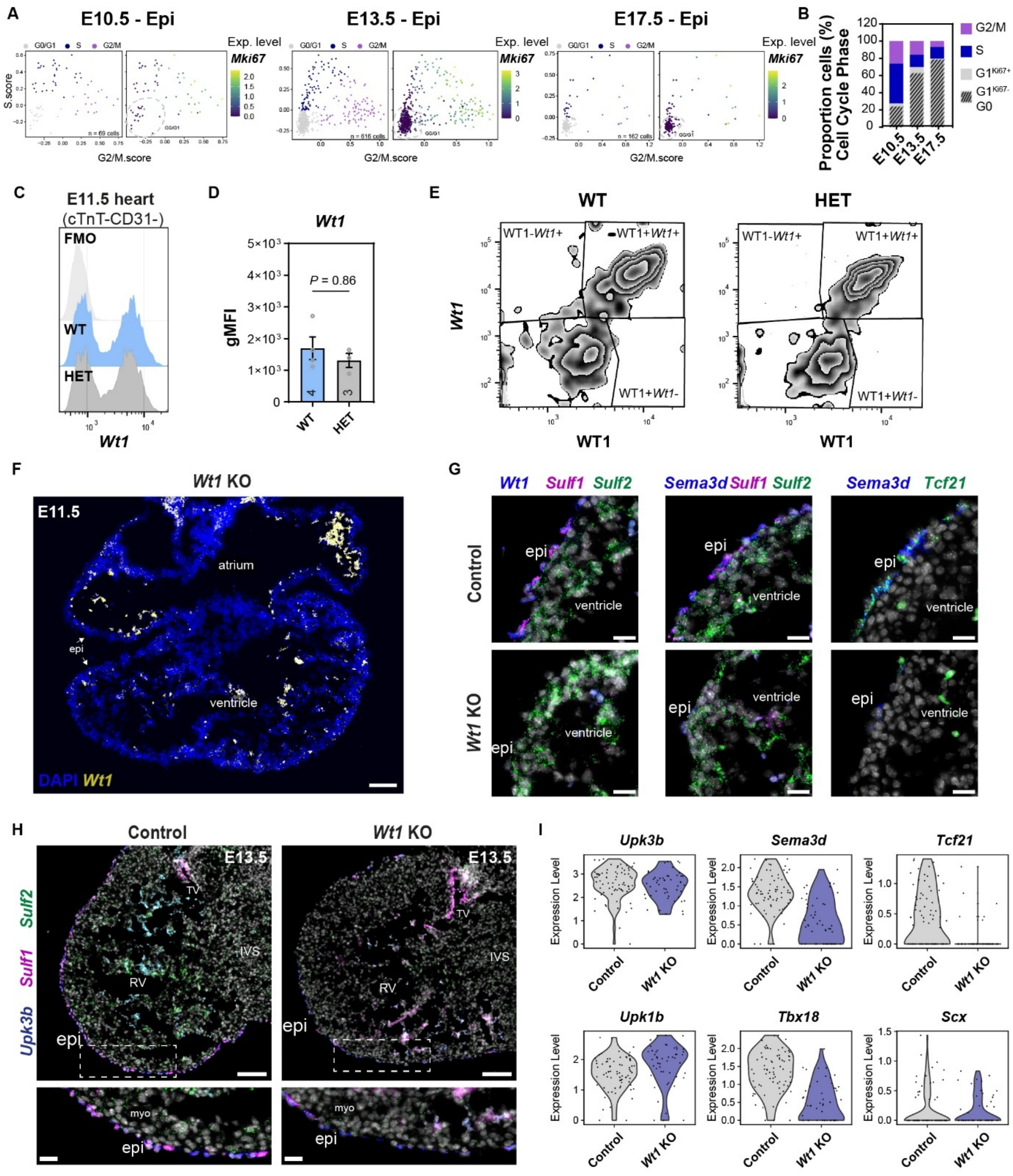

Figure S6 *Wt1* KO hearts demonstrate a dysregulated epicardium with reduced expression of 'classical' epicardial markers

(A) Scatter plot showing the distribution of epicardial cells in the indicated cell cycle phases at E10.5, E13.5 and E17.5. Feature plot representing range of expression of *Mki67*, further separating quiescent and slowly-dividing epicardial cells. (B) Percentage of epicardial cells in the indicated cell cycle phases. (C) Flow cytometry analysis of control hearts at E11.5, and corresponding expression of *Wt1* in non-myocyte and non-endothelial cells (cTnT-CD31-) enriched with epicardial cells. Wild-type (WT; *Wt1*$^{+/+}$, light blue), Heterozygous (HET;

Wt1$^{CreERT2/+}$, dark grey) and Fluorescence minus one (FMO) control shown (light grey). (D) Population expression of *Wt1* quantified as geometric mean fluorescence intensity (gMFI). Data are represented as mean ± SEM with individual data points and sample size indicated (n = 4 WT and n = 3 HET; pooled average of 8 hearts per replicate). Mann-Whitney *U* test, with *P* values indicated. (E) Flow cytometry analysis of control hearts at E11.5, and corresponding correlation between *Wt1* transcript and WT1 protein levels in non-myocyte and non-endothelial cells (cTnT-CD31-) enriched with epicardial cells. n = 2; pooled average of 4 hearts per replicate. (F) Fluorescence ISH of E11.5 hearts for *Wt1* showing complete loss of expression in *Wt1* KO hearts. Scale bars: 100 µm. (G) Fluorescence ISH of E11.5 hearts characterising *Sulf1* and *Sulf2* expression alongside other 'classical' epicardial markers *Sema3d* and *Tcf21*. Scale bars: 25 µm. Representative images were chosen according to the following criteria: correct anatomical region, accurate representation of the phenotype, high quality, and preserved tissue or cellular integrity. n = 3 control and n = 2 *Wt1* KO hearts. (H) Fluorescence ISH of E13.5 hearts for *Upk3b*, *Sulf1* and *Sulf2,* showing an incomplete ventricular epicardium in *Wt1* KO hearts with reduced *Sulf1* expression. Scale bars: 100 µm. Representative images were chosen according to the following criteria: correct anatomical region, accurate representation of the phenotype, high quality, and preserved tissue or cellular integrity. n = 2 control and n = 2 *Wt1* KO hearts. (I) Violin plots showing relative expression of epicardial markers in the control and *Wt1* KO epicardial cluster (E14.5 scRNA-seq).

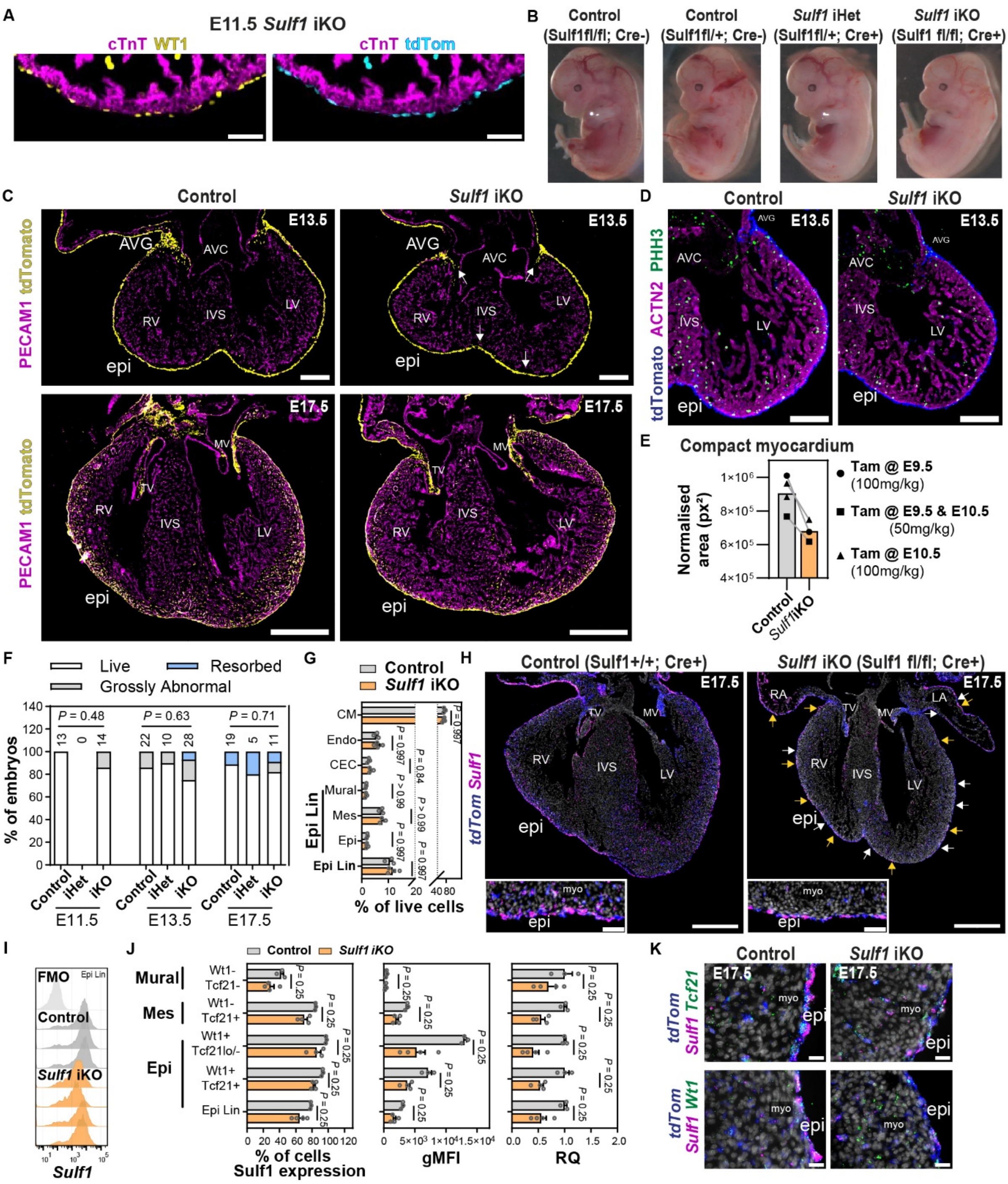

Figure S7 *Sulf1* iKO hearts demonstrate mosaicism and compensation as development progresses

(A) Immunofluorescence images showing WT1+tdTomato+ epicardial cells on the surface of *Sulf1* iKO hearts at E11.5. Region of interest of the left ventricular apex. Scale bar, 50 µm. Representative images were chosen according to the following criteria: correct anatomical region, accurate representation of the phenotype and/or mean, high quality, and preserved tissue or cellular integrity. (B) Images representative of E13.5 littermate embryos and their associated genotype, showing no gross abnormalities or overt extra-cardiac defects in *Sulf1* iKO embryos. Representative images were chosen according to the following criteria: correct

embryonic stage, litter encompassing full genotype outcomes, accurate representation of the phenotype, high quality, and preserved anatomy and tissue integrity. 4 litters. (C) Immunofluorescence images showing tdTomato-labelled epicardium and derivatives in control and *Sulf1* iKO embryonic mouse hearts at E13.5 and E17.5. White arrows highlight reduced invasion of EPDCs at the AVG, subepicardium and IVS. Scale bar, 200 µm (E13.5) and 500 µm (E17.5). Images representative of n = 2 hearts. (D) Immunofluorescence images showing mitotic cells expressing PHH3 in control and *Sulf1* iKO embryonic mouse hearts at E13.5. Scale bar, 200µm. Images representative of n = 2 hearts. Representative images were chosen according to the following criteria: correct anatomical region, accurate representation of the phenotype, high quality, and preserved tissue or cellular integrity (C through D). (E) Quantification of the compact myocardial area in the left ventricle of control and *Sulf1* iKO hearts at E13.5. Compact myocardial area was corrected using a normalization factor based on the length of the left ventricular surface. Data from littermate pairs are connected by lines, and different symbols indicate the tamoxifen regimens used. (F) Percentage of embryos categorised as live, grossly abnormal or resorbed, and their associated genotype at E11.5, E13.5 and E17.5. Number of embryos annotated at the top of each stacked bar. 6-9 litters. Fisher's exact test (comparison between control and *Sulf1* iKO), with *P* values indicated. (G) Flow cytometry analysis of control and *Sulf1* iKO hearts at E17.5, and corresponding quantification of epicardial-lineage cells and other major cardiac cell types. The epicardial lineage was selected by gating $CD31^-cTnT^-tdTomato^+$, with subsequent gating of mesenchymal cells (Dpt^+Rgs5^-) and mural cells ($Rgs5^+$). Endothelial cells were selected by gating $CD31^+Npr3^{-/low}$ for coronary (CEC) and lymphatic endothelial cells (LEC), and $CD31^+Npr3^+$ for endocardial (Endo) and valve endothelial cells (VEC). Cardiomyocytes were selected by gating cells positive for cTNT. Data are represented as mean ± SEM with individual data points indicated (pooled 2 hearts per control replicate; n = 3 control and n = 4 *Sulf1* iKO replicates; across 2 litters per group). Mann-Whitney test with Holm-Šídák correction for multiple comparisons (7 comparisons), with adjusted *P* values indicated. (H) Fluorescence ISH of E17.5 control and *Sulf1* iKO heart cryosections for *tdTomato* and *Sulf1*, showing a mosaic expression profile of *Sulf1* in the epicardium. White arrows highlight epicardial cells with undetectable *Sulf1* transcripts, and yellow arrows highlight epicardial cells expressing *Sulf1* comparable to control. Scale bars: 500 µm and inset 50 µm. n = 1. (I) Flow cytometry analysis of control and *Sulf1* iKO hearts at E17.5, and corresponding expression of *Sulf1* in epicardial-lineage cells (Epi Lin). Fluorescence minus one (FMO) control shown (light grey). (J) Flow cytometry analysis of control and *Sulf1* iKO hearts at E17.5, and corresponding quantification of percentage positive epicardial-lineage cells for *Sulf1*. Population expression of *Sulf1* quantified as geometric mean fluorescence intensity (gMFI), and expressed relative to control hearts (RQ). Data are represented as mean ± SEM with individual data points

indicated (pooled 2 hearts per control replicate; n = 3 control and n = 4 *Sulf1* iKO replicates; across 2 litters per group). Mann-Whitney test with Holm-Šídák correction for multiple comparisons (7 comparisons), with adjusted *P* values indicated. (K) Fluorescence ISH of E17.5 control and *Sulf1* iKO heart cryosections for *tdTomato*, *Sulf1*, *Tcf21* and *Wt1*, showing expression of *Sulf1* in the epicardium and its derivative mesenchymal cells invading the myocardium. Scale bars: 20 µm. n = 1.

epi, epicardium; myo, myocardium; RA, right atrium; RV, right ventricle; IVS, interventricular septum; LV, left ventricle; AVC, atrioventricular canal; AVG, atrioventricular groove; TV, tricuspid valves; MV, mitral valve.

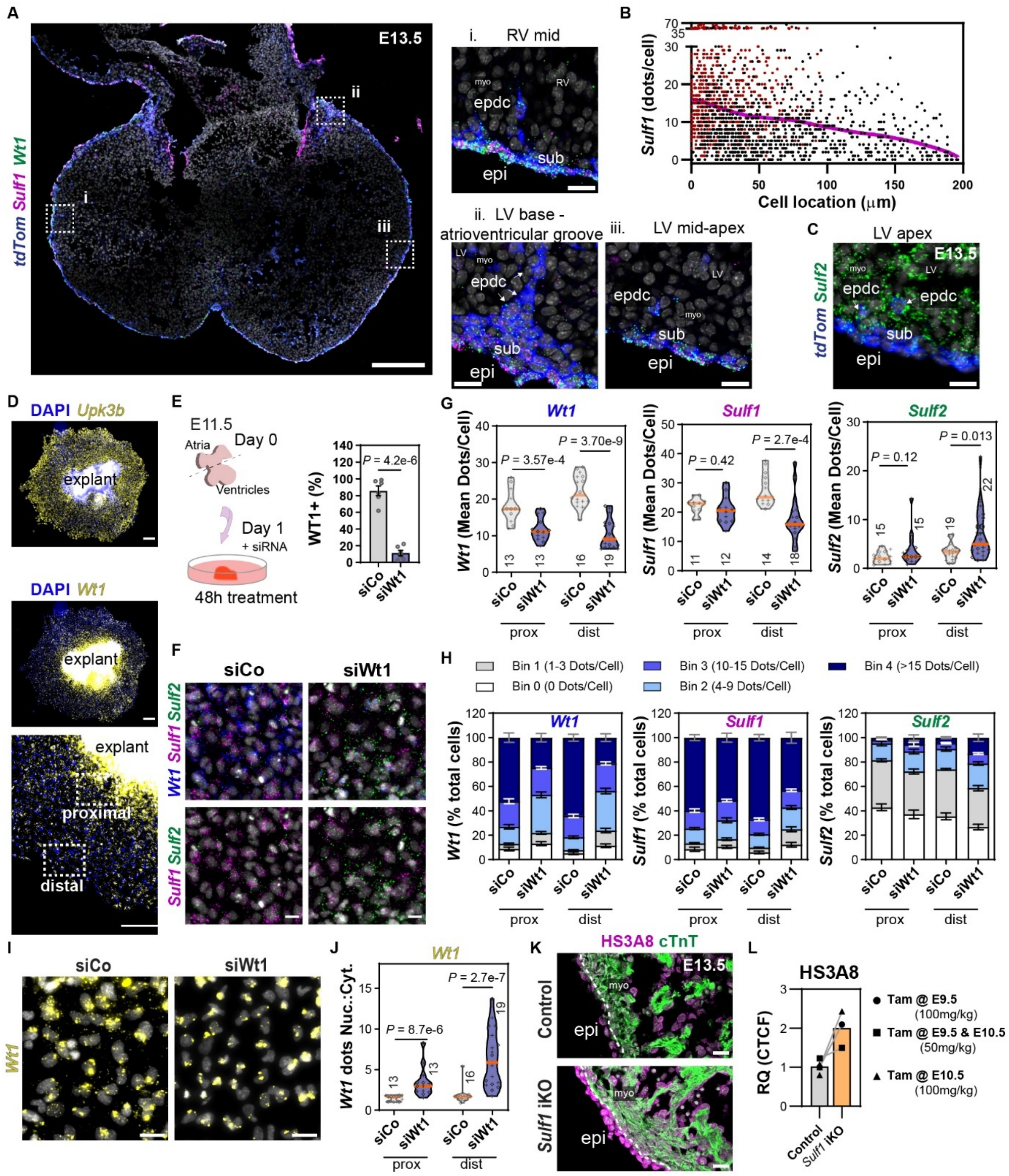

Figure S8 Diminishing expression of *Sulf1* and *Wt1* coincides with epicardial EMT

(A) Fluorescence ISH of E13.5 heart cryosections for *tdTomato*, *Sulf1* and *Wt1*, showing downregulated expression of *Wt1* and *Sulf1* in epicardial-lineage subepicardial (sub) and mesenchymal cells invading the myocardium (epdc). Scale bars: 20 µm. Representative images were chosen according to the following criteria: correct anatomical region, accurate representation of the mean, high quality, and preserved tissue or cellular integrity. Images representative of n = 4 hearts. (B) Scatter plot showing quantification of *Sulf1* expression in ventricular *tdTomato* positive epicardial and derivative cells at E13.5 (using Fluorescence ISH images; ROI base, mid and apex). Data of individual cells from n = 5 hearts, and at least three

ROI images per heart. Cell location is indicated by the Y center coordinate within each ROI image: values of *y*<100µm predominantly correspond to cells in the epicardium and subepicardium, while higher *y* values reflect increasing depth into the myocardium. A magenta LOWESS smoothing line models *Sulf1* expression in epicardium-lineage cells along their spatial distribution from the epicardium toward the endocardium. Epicardial cells are shown in red. (C) Fluorescence ISH of E13.5 heart cryosections for *tdTomato* and *Sulf2*, showing upregulated expression of *Sulf2* in epicardial-lineage subepicardial (sub) and mesenchymal cells invading the myocardium (epdc). Scale bars: 20 µm. Representative images were chosen according to the following criteria: correct anatomical region, accurate representation of the mean, high quality, and preserved tissue or cellular integrity. Images representative of n = 5 hearts. (D) Fluorescence ISH of E11.5 epicardial explants for *Upk3b* and *Wt1*, showing highest *Wt1* expression proximal to the explant piece. Scale bars, 200 µm. (E) E11.5 atrial and ventricular epicardial explants transfected with control (siCo) or Wt1 siRNA (siWt1) for 48h. Quantification of percentage WT1 positive epicardial cells in control and Wt1 siRNA-transfected explants showing successful knock-down. Data are represented as mean ± SEM with individual data points indicated (n = 6 explants; 3 ventricular and 3 atrial epicardial explants). Unpaired Welch t test, with *P* values indicated. (F) Fluorescence ISH of control and Wt1 siRNA-transfected epicardial explants for *Wt1*, *Sulf1* and *Sulf2,* and (G) corresponding quantification of expression levels. Violin plot symbol cross (+) represents atrial explant; diamond (◊) represents ventricular explant. Orange line indicates the median. Unpaired Welch t test with Holm-Šídák correction for multiple comparisons (2 comparisons), with adjusted *P* values indicated. (H) Percentage of epicardial cells expressing *Sulf1* and *Sulf2*, grouped into 5 bins based on the number of dots (individual RNA molecules) per cell. Scale bars, 20 µm. Images representative of n = 14-19 explants. Data are represented as mean ± SEM; the sample size (explants) per group is indicated in G and is identical for H (D through G). (I) Fluorescence ISH of control and Wt1 siRNA-transfected epicardial explants showing *Wt1* expression, with corresponding quantification of (J) *Wt1* transcript localisation across cell compartments. Scale bars, 20 µm. Images representative of n = 13-19 explants. Violin plot symbol cross (+) represents atrial explant; diamond (◊) represents ventricular explant. Orange line indicates the median; the sample size (explants) per group is indicated. Mann-Whitney test with Holm-Šídák correction for multiple comparisons (2 comparisons), with adjusted *P* values indicated (K) Immunofluorescence images showing anti-heparan sulfate staining (HS3A8) in the epicardium of control and *Sulf1* iKO hearts at E13.5, and (L) relative quantification of corrected total cellular fluorescence (CTCF). Scale bars: 20 µm. Representative images were chosen according to the following criteria: correct anatomical region, accurate representation of the phenotype and/or mean, high quality, and preserved

tissue or cellular integrity. n=5 control and n = 3 *Sulf1* iKO hearts. Data from littermate pairs are connected by lines, and different symbols indicate the tamoxifen regimens used.

epi, epicardium; myo, myocardium; sub, subepicardium; epdc, epicardium-derived cells; RV, right ventricle; LV, left ventricle; prox, proximal; dist, distal.

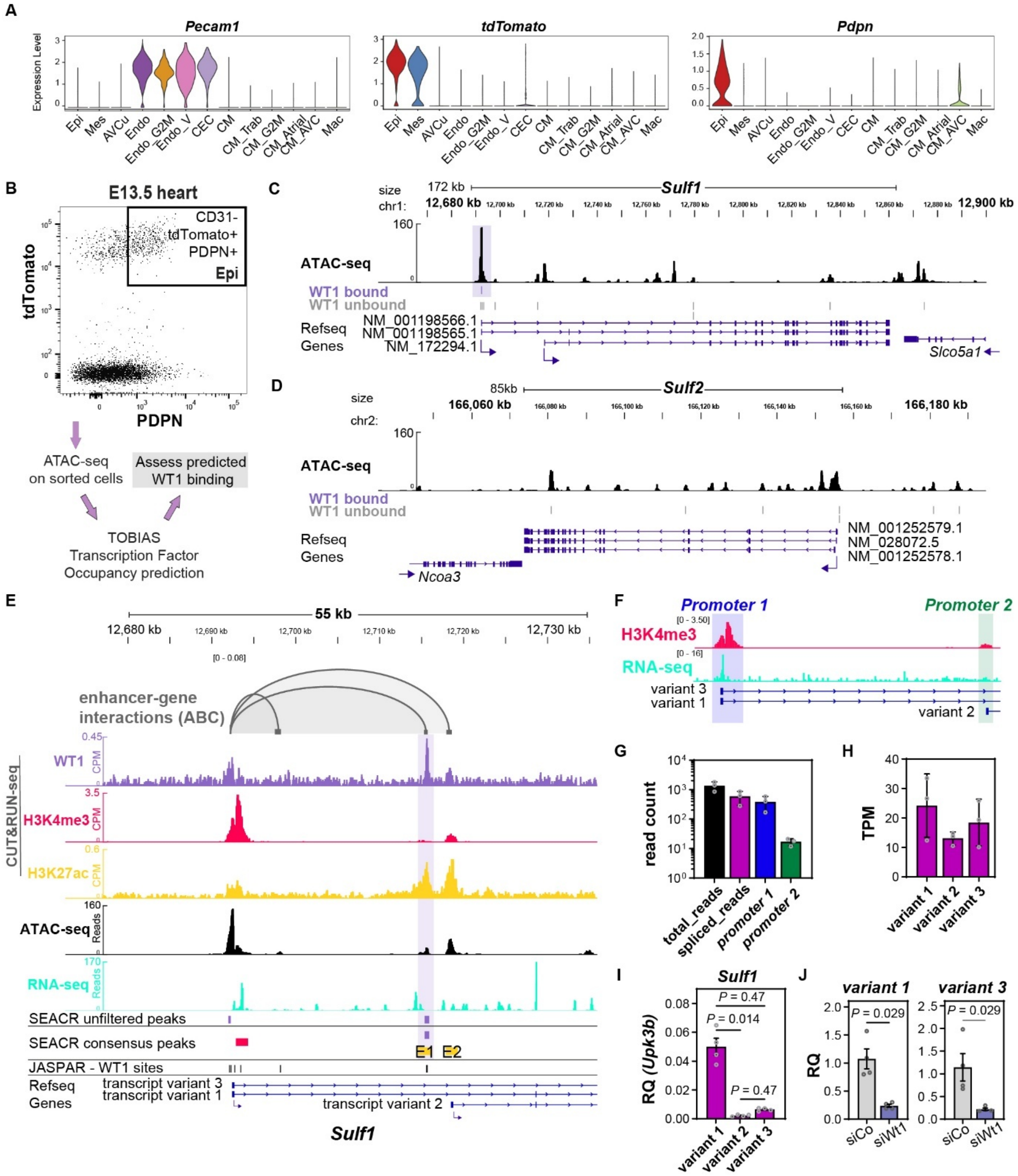

Figure S9 Identification of WT1 associated CREs regulating *Sulf1* and *Sulf2* transcription in the epicardium

(A) Violin plots showing relative expression of markers used to distinguish epicardium in E13.5 hearts. (B) Strategy used to gate and purify epicardial cells (CD31^{-}tdTomato^{+}PDPN^{+}) from E13.5 hearts. Purified epicardial cells were processed for ATAC-seq analysis, and downstream transcription factor occupancy prediction to assess binding of WT1 in the *Sulf1* and *Sulf2* loci. (C-D) Genome tracks showing predicted binding of WT1 at the (B) *Sulf1* and (C) *Sulf2* locus in sorted E13.5 epicardial cells. Track signal is in reads per million (scaled) and merged from n = 2 independent experimental replicates. TOBIAS predicted WT1 binding

calculated from n = 3 independent experimental replicates (n = 2, E13.5; n = 1, E12.5). Region of interest (ROI) with bound WT1 indicated with purple shading. (E) Genome tracks of CUT&RUN-seq, ATAC-seq and RNA-seq showing direct binding of WT1 to intronic enhancer (E1) at the *Sulf1* locus of epicardial cells. Chromatin interaction tracks show linkages between WT1-bound enhancer E1 and upstream *Sulf1* promoter (ABC). All linkages shown have an ABC score >0.04. SEACR consensus peaks calculated from n = 3 (WT1, IgG and H3K27ac) and n = 4 (H3K4me3) independent experimental replicates. JASPAR database WT1 binding sites indicated. (F) Genome tracks of CUT&RUN-seq and RNA-seq showing enriched transcription through Promoter 1. n = 4 (H3K4me3) and n = 3 (RNA-seq) independent experimental replicates. (G) Mean read counts showing transcription in the *Sulf1* locus, with greatest number of reads detected in the Promoter 1 region. (H) RNA-seq quantification of *Sulf1* transcript variants. Data are shown as Transcripts Per Million (TPM) and represented as mean ± SD with individual data points indicated. (I) qRT-qRT-PCR analysis of *Sulf1* transcript variants expressed relative to *Upk3b*. n=4 experimental replicates. Data are represented as mean ± SEM with individual data points indicated. Friedman test with Dunn's correction for multiple comparisons (3 comparisons), with adjusted *P* values indicated (J) MEC1 epicardial cells transfected with control (siCo) or Wt1 siRNA (siWt1) for 48h. qRT-PCR analysis of Sulf1 transcript variant 1 and variant 3 relative expression in Wt1 siRNA-transfected epicardial cells. n = 4 experimental replicates. Data are represented as mean ± SEM with individual data points indicated. Mann-Whitney *U* test, with *P* values indicated.

AVCu, atrioventricular cushion; CEC, coronary endothelial cells; CM, ventricular cardiomyocytes; CM_Atrial, atrial cardiomyocytes; CM_AVC_sV, atrioventricular canal and sinus venosus cardiomyocytes; CM_Trab, trabecular cardiomyocytes; Endo, endocardial cells; Endo_V, valvular endothelial and endocardial cells; Epi, epicardium; G2M, proliferating; Mac, macrophages; Mes, mesenchyme.

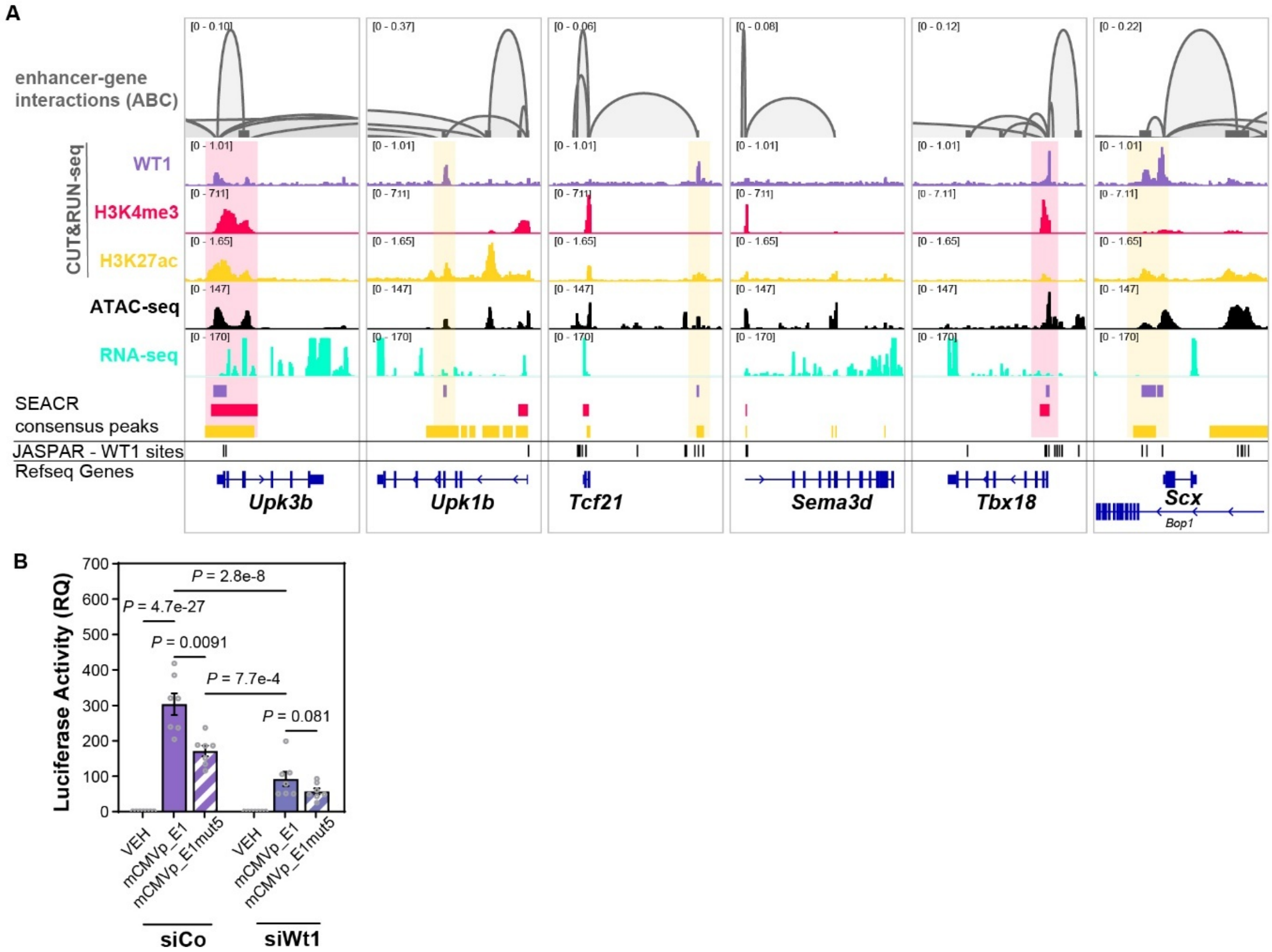

Figure S10 Identification of WT1 associated CREs regulating transcription in the epicardium

(A) Genome tracks of CUT&RUN-seq, ATAC-seq and RNA-seq showing direct binding of WT1 to active promoters, promoter-proximal and distal enhancers of epicardial marker gene loci. Chromatin interaction tracks show linkages between WT1-bound CREs and gene promoters (ABC). All linkages shown have an ABC score >0.04. SEACR consensus peaks calculated from n = 3 (WT1, IgG and H3K27ac) and n = 4 (H3K4me3) independent experimental replicates. JASPAR database WT1 binding sites indicated. (B) Epicardial cell line transfected with control (siCo) or Wt1 siRNA (siWt1) for 24h, followed by transfection with mCMVp_E1 and mCMVp_E1mut5 for 24h. Corresponding quantification of Luciferase activity to assess transcriptional activity of WT1 on intronic *Sulf1* enhancer in the epicardial cell line. n = 7 experimental replicates. Values expressed relative to vehicle control. Data are represented as mean ± SEM with individual data points indicated. 1-way ANOVA test with Šídák correction for multiple comparisons (5 comparisons), with adjusted *P* values indicated.

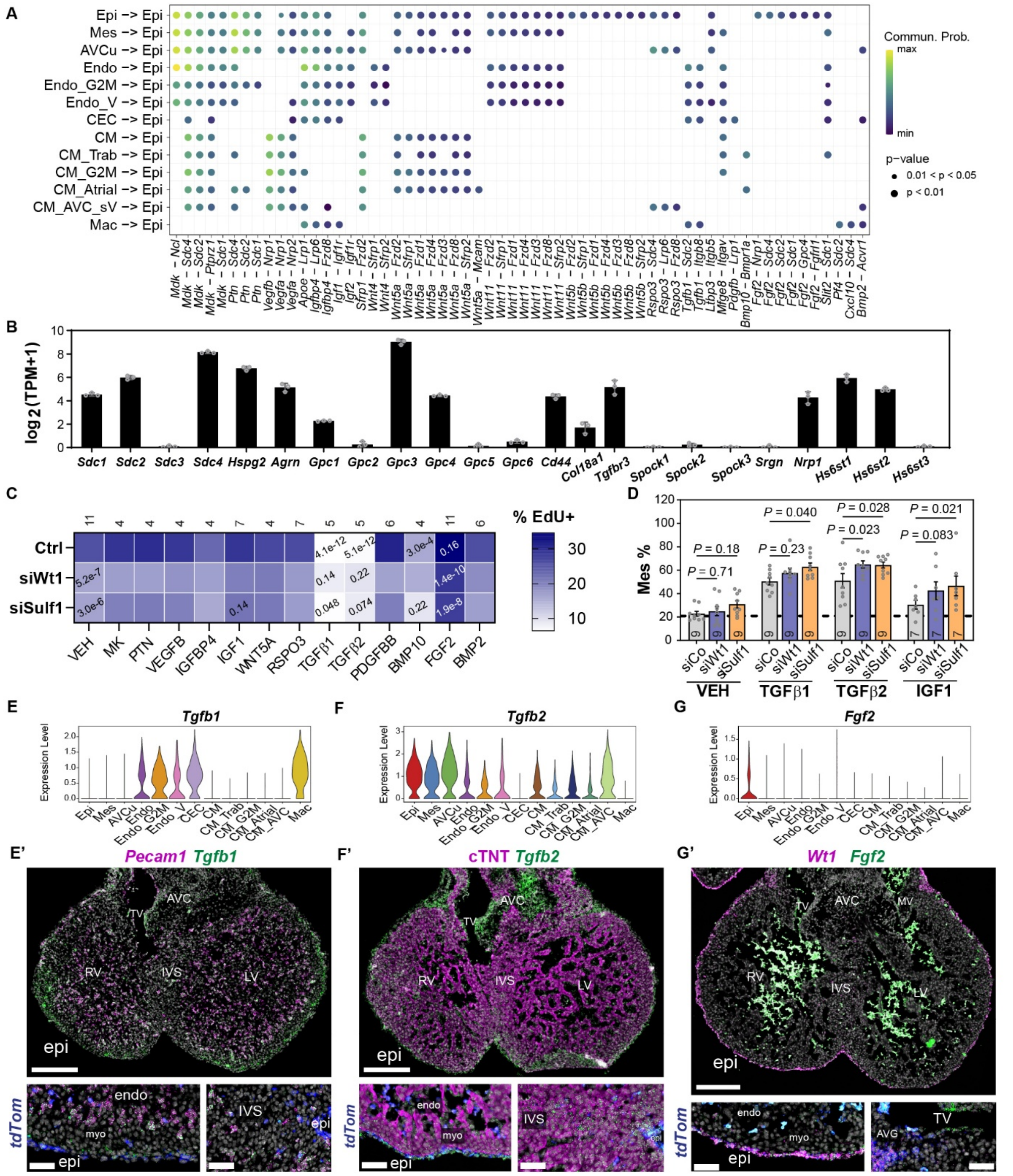

Figure S11 Ligand-Receptor inference analysis identified HSPG-dependent incoming signalling and potential cell type sources in the E13.5 heart

(A) Dot plot indicating source cell types communicating with the epicardium and corresponding key ligand-receptor pairs. High communication probability is indicated in yellow, dot size indicates p-values. (B) RNA-seq quantification of HSPGs and HS6STs expressed in MEC1 epicardial cells. Data are shown as Transcripts Per Million (TPM) and represented as mean ± SD with individual data points indicated (n = 3 independent experimental replicates). (C) Epicardial cells transfected with control (siCo), Wt1 (siWt1) or Sulf1 siRNA (siSulf1) for 48h, followed by treatment with candidate cytokines for 24h, and corresponding quantification of

percentage EdU positive cells. Heatmap data expressed as mean and sample size (experimental replicates) indicated. ART ANOVA test with Holm-Šídák correction for multiple comparisons (41 comparisons; includes comparison across siRNA treatments in the Vehicle group), with select adjusted *P* values (*P*<0.25) indicated and found in Table S7. (D) Epicardial cells transfected with siRNAs for 48h, followed by treatment with candidate cytokines for 24h, and corresponding quantification of percentage mesenchymal cells. Data are represented as mean ± SEM with individual data points and sample size (experimental replicates) indicated. 2-Way ANOVA and Fisher's LSD test, with *P* values indicated. (E) Violin plot showing relative expression of *Tgfb1* in hearts at E13.5, and corresponding (E') fluorescence ISH of heart cryosections for *Pecam1* and *Tgfb1*, showing majority source of *Tgfb1* from endocardial and coronary endothelial cells. Insets highlight proximity of epicardial-lineage cells (*tdTomato* positive) to *Tgfb1*-expressing endothelial cells. (F) Violin plot showing relative expression of *Tgfb2* in hearts at E13.5, and corresponding (F') fluorescence ISH of heart cryosections for cTNT and *Tgfb2*, showing widespread expression of *Tgfb2* throughout the ventricles. Insets highlight enriched expression in the epicardial layer and apical region of the intraventricular septum. (G) Violin plot showing relative expression of *Fgf2* in hearts at E13.5, and corresponding (G') fluorescence ISH of heart cryosections for *Wt1* and *Fgf2*, showing two specific sources of *Fgf2*, the epicardium and atrioventricular valves. Insets highlight localised expression in epicardial and valve endothelial cells. Scale bars: 200 µm and inset 50 µm. Representative images were chosen according to the following criteria: correct anatomical region, accurate representation of the mean, high quality, and preserved tissue or cellular integrity. Images representative of n = 3 hearts (E' through G').

AVCu, atrioventricular cushion; CEC, coronary endothelial cells; CM, ventricular cardiomyocytes; CM_Atrial, atrial cardiomyocytes; CM_AVC_sV, atrioventricular canal and sinus venosus cardiomyocytes; CM_Trab, trabecular cardiomyocytes; Endo, endocardial cells; Endo_V, valvular endothelial and endocardial cells; Epi, epicardium; G2M, proliferating; Mac, macrophages; Mes, mesenchyme; epi, epicardium; myo, myocardium; endo, endocardium; RV, right ventricle; IVS, interventricular septum; LV, left ventricle; AVC, atrioventricular canal; AVG, atrioventricular groove; TV, tricuspid valves; MV, mitral valve.

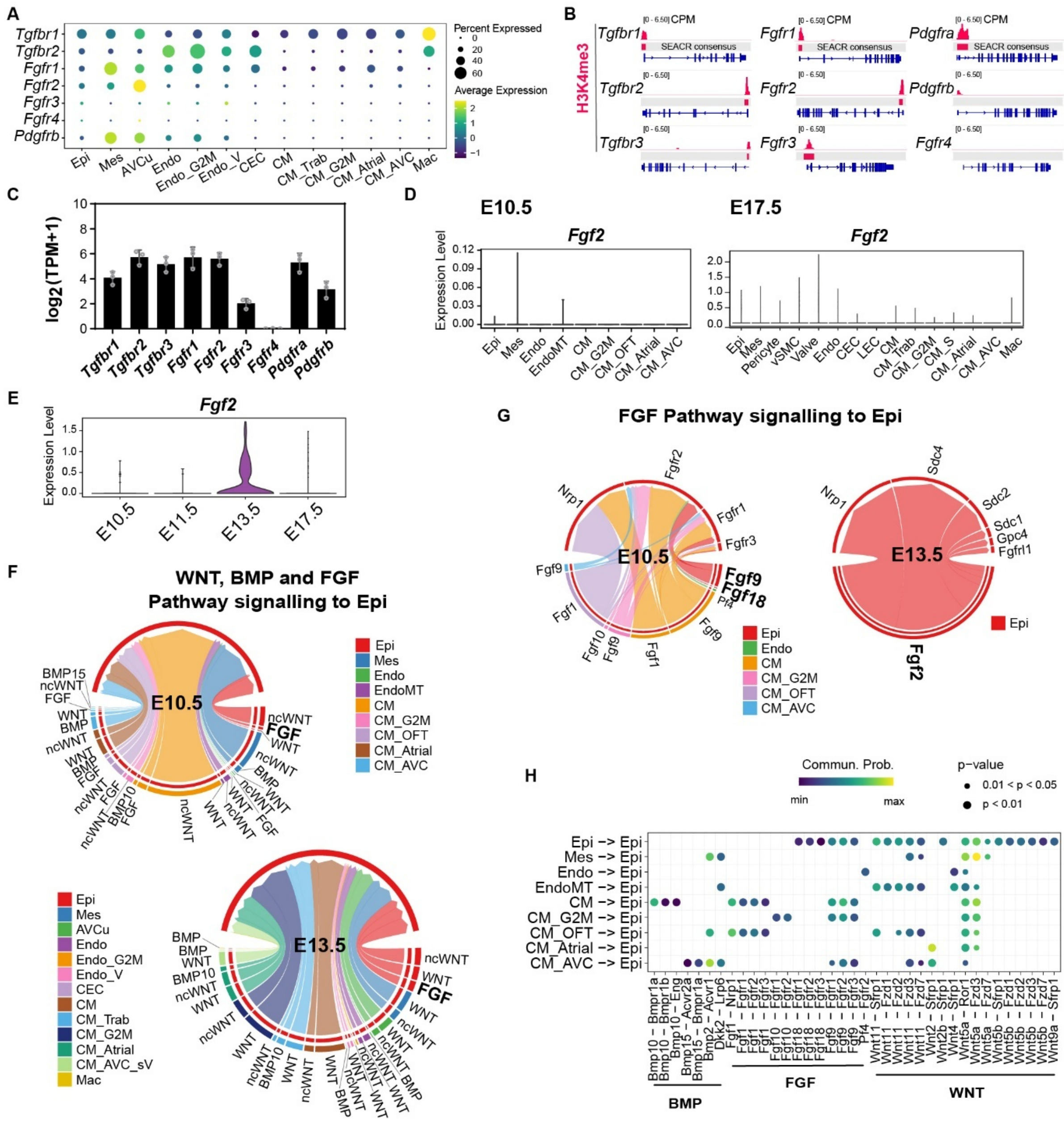

Figure S12 HSPG-dependent incoming signalling mediating epicardial changes are temporal with FGF2 being stage-specific

(A) Dot plot showing relative expression of growth factor receptors in hearts at E13.5. High expression is indicated in yellow, dot size indicates percentage expressed. (B) Genome tracks showing H3K4me3 profiles at *Tgfbr1, Tgfbr2, Tgfbr3, Fgfr1, Fgfr2, Fgfr3, Fgfr4, Pdgfra* and *Pdgfrb* locus. Track signal is in counts per million (CPM) and merged from n = 2 independent experimental replicates. SEACR consensus peaks calculated from n = 4 (H3K4me3 and IgG) independent experimental replicates. (C) RNA-seq quantification of TGFβ, FGF and PDGF receptors expressed in MEC1 epicardial cells. Data are shown as Transcripts Per Million (TPM) and represented as mean ± SD with individual data points indicated (n = 3 independent experimental replicates). (D) Violin plots showing relative expression of *Fgf2* in the developing mouse heart at E10.5 and E17.5. (E) Violin plots showing relative expression of *Fgf2* in

epicardial cells at E10.5, E11.5, E13.5 and E17.5. (F) Chord diagrams showing WNT, BMP and FGF pathway signalling to the epicardium at E10.5 and E13.5. (G) Chord diagrams showing FGF ligand-receptor pairs signalling to the epicardium at E10.5 and E13.5. The inner bar size is proportional to the signal strength received by epicardial cells (F through G). (H) Dot plot indicating source cell types communicating with the epicardium and corresponding key ligand-receptor pairs in WNT, BMP and FGF pathway signalling at E10.5. High communication probability is indicated in yellow, dot size indicates raw p-values (Permutation test; Table S5).

Major Resources Tables

Animals

Resource	Vendor/Source	Catalog # / Identifier
Mouse: Gt(ROSA)26Sor$^{tm14(CAG-tdTomato)Hze}$/J	The Jackson Laboratory	MGI ID: 3809524<br>JAX Stock No: 007914<br>RRID:IMSR_JAX: 007914
Mouse: Gt(ROSA)26Sor$^{tm9(CAG-tdTomato)Hze}$/J	The Jackson Laboratory	MGI ID: 3809523<br>JAX Stock No: 007909<br>RRID:IMSR_JAX: 007909
Mouse: Wt1$^{tm2(cre/ERT2)Wtp}$/J	The Jackson Laboratory	MGI ID: 3801682<br>JAX Stock No: 010912<br>RRID:IMSR_JAX: 010912
Mouse: Sulf1$^{tm1a(KOMP)Mbp}$ and Sulf1$^{tm1c(KOMP)Mbp}$	KOMP<br>This paper	MGI ID: 5141826<br>MMRRC: 063141-UCD<br>Parental Cell Line JM8A3.N1.<br>ES Cell Clones:<br>DEPD00568_2_A12<br>DEPD00568_2_H12
Mouse: Tg(ACTFLPe)9205Dym	The Jackson Laboratory	MGI ID: 2448985<br>JAX Stock No: 005703<br>RRID:IMSR_JAX: 005703
Mouse: C57BL/6J Wild-types	Charles River	-

Antibodies & Dyes

Reagent	Vendor/Source	Catalog # / Identifier
Anti-Wilms Tumor Protein antibody [CAN-R9(IHC)-56-2] (1:100, IF; 1:50, Cut&Run)	Abcam	ab89901<br>RRID:AB_2043201
Anti-CD31 antibody [2H8] (1:100, IF)	Abcam	ab119341<br>RRID:AB_10900179
Anti-smooth muscle Myosin heavy chain 11 antibody (1:200, IF)	Abcam	ab125884
Ki-67 Monoclonal Antibody (SolA15) (1:100, IF)	Invitrogen eBioscience	14-5698-82<br>RRID:AB_10854564
p-Histone H3 Antibody (Ser 10) (1:100, IF)	Santa Cruz	sc-8656<br>RRID:AB_653256
Anti-Sarcomeric Alpha Actinin antibody [EA-53] (1:100, IF)	Abcam	ab9465<br>RRID:AB_307264
Endomucin Antibody (V.7C7) (1:100, IF)	Santa Cruz	sc-65495<br>RRID:AB_2100037
Alexa Fluor® 647 Mouse Anti-Cardiac Troponin T (1:100, IF)	BD Biosciences	565744<br>RRID:AB_2739341
Anti-beta Catenin antibody [E247] - ChIP Grade (1:250, IF)	Abcam	ab32572<br>RRID:AB_725966
Anti-Actin, α-Smooth Muscle - Cy3™ antibody (1:400, IF)	Sigma-Aldrich	C6198<br>RRID:AB_476856
Anti-Wilms tumor protein Alexa Fluor 488 (CAN-R9(IHC)-56-2) antibody (1:100, FC)	Abcam	ab202635<br>RRID:AB_2889326

Anti-Cardiac Troponin T BV421 (13-11) (1:200, FC)	BD Biosciences	Cat# 565618<br>RRID:AB_2739306
Brilliant Violet 605™ anti-mouse CD31 (1:50, FC)	Biolegend	Cat# 102427<br>RRID:AB_2563982
Brilliant Violet 421™ anti-mouse CD31 (1:50, FC)	Biolegend	Cat# 102424<br>RRID:AB_2650892
FITC anti-mouse CD31 (1:50, FC)	Biolegend	Cat# 102406<br>RRID:AB_312901
APC/Cy7 anti-mouse Podoplanin (1:100, FC)	Biolegend	Cat# 127418<br>RRID:AB_2629804
TruStain FcX (1:50, FC)	Biolegend	Cat# 101319<br>RRID:AB_1574973
Zombie Aqua Fixable Viability Kit (1:1000, FC)	Biolegend	Cat# 423101
SYTOX™ Green Dead Cell Stain (1:50000)	ThermoFisher Scientific/ Invitrogen	Cat# S34860
Rabbit (DA1E) mAb IgG XP® Isotype Control (1:20, Cut&Run)	Cell Signaling Technology	Cat# 66362<br>RRID:AB_2924329
Tri-Methyl-Histone H3 (Lys4) (C42D8) Rabbit mAb (1:50, Cut&Run)	Cell Signaling Technology	Cat# 9751<br>RRID:AB_2616028
Acetyl-Histone H3 (Lys27) (D5E4) XP® Rabbit mAb (1:100, Cut&Run)	Cell Signaling Technology	Cat# 8173<br>RRID:AB_10949503
Phospho-Smad2 (Ser465/467) (138D4) Rabbit mAb (1:1000, WB)	Cell Signaling Technology	Cat# 3108<br>RRID:AB_490941
Smad2 (L16D3) Mouse mAb (1:1000, WB)	Cell Signaling Technology	Cat# 3103<br>RRID:AB_490816
Anti-beta Actin antibody [mAbcam 8224] - Loading Control (1:2500, WB)	abcam	Cat# ab8224<br>RRID:AB_449644
AO4B08 (1:10 for cryosections, 1:25 for cells, IF)	Dr. Toin van Kuppevelt	
HS3B8 (1:10 for cryosections, 1:25 for cells, IF)	Dr. Toin van Kuppevelt	
Monoclonal anti-VSV glycoprotein clone P5D4 Cy3 (1:100, IF)	Sigma-Aldrich	Cat#:C7706<br>RRID:AB_259043
VSV-G Epitope Tag Antibody Dylight(TM) 488 Conjugated (1:250, IF)	Rockland Immunochemicals	Cat#: 600-441-386<br>RRID:AB_1961807
Podoplanin Monoclonal Antibody (eBio8.1.1 (8.1.1), 1:100, IF)	ThermoFisher Scientific/ Invitrogen	Cat#: 14-5381-82<br>RRID:AB_1210505
Donkey anti-Rabbit IgG (H+L) Highly Cross-Adsorbed Secondary Antibody, Alexa Fluor™ 488 (1:200-1:400, IF)	ThermoFisher Scientific/ Invitrogen	A21206<br>RRID:AB_2535792
Donkey anti-Rabbit IgG (H+L) Highly Cross-Adsorbed Secondary Antibody, Alexa Fluor 546 (1:200-1:400, IF)	ThermoFisher Scientific/ Invitrogen	A10040<br>RRID:AB_2534016
Donkey anti-Rabbit IgG (H+L) Highly Cross-Adsorbed Secondary Antibody, Alexa Fluor 647 (1:200-1:400, IF)	ThermoFisher Scientific/ Invitrogen	A31573<br>RRID:AB_2536183
Donkey anti-Rat IgG (H+L) Highly Cross-Adsorbed Secondary Antibody, Alexa Fluor™ 488 (1:200-1:400, IF)	ThermoFisher Scientific/ Invitrogen	A21208<br>RRID:AB_2535794
Donkey anti-Rat IgG (H+L) Cross-Adsorbed Secondary Antibody, DyLight 650 (1:50, IF)	ThermoFisher Scientific/ Invitrogen	SA5-10029<br>RRID:AB_2556609
Goat Anti-Armenian hamster IgG H&L (Alexa Fluor 488) (1:200-1:400, IF)	Abcam	ab173003<br>RRID:AB_2936402
Goat Anti-Armenian hamster IgG H&L (Alexa Fluor 647) (1:200-1:400, IF)	Abcam	ab173004<br>RRID:AB_2732023

Goat anti-Syrian Hamster IgG (H+L) Cross-Adsorbed Secondary Antibody, Alexa Fluor™ 488 (1:200-1:400, IF)	ThermoFisher Scientific/ Invitrogen	A21110<br>RRID:AB_2535759
Goat anti-Syrian Hamster IgG (H+L) Cross-Adsorbed Secondary Antibody, Alexa Fluor™ 647 (1:200-1:400, IF)	ThermoFisher Scientific/ Invitrogen	A21451<br>RRID:AB_2535868
Donkey anti-Mouse IgG (H+L) Highly Cross-Adsorbed Secondary Antibody, Alexa Fluor 647 (1:200-1:400, IF)	ThermoFisher Scientific/ Invitrogen	A31571<br>RRID:AB_162542

RNAscope & PrimeFlow Probes

Probe/Reagent	Vendor/Source	Catalog #
RNAscope 3-plex Negative Control Probe	Bio-Techne	320871
Mm-Upk3b	Bio-Techne	568561
Mm-Wt1	Bio-Techne	432711<br>432711-C2<br>416051-C3
Mm-Sulf1	Bio-Techne	495411<br>495411-C2
Mm-Sulf2	Bio-Techne	467901-C3
Mm-Postn	Bio-Techne	418581-C2
Mm-Dpt	Bio-Techne	561511
Mm-Rgs5	Bio-Techne	430181
tdTomato	Bio-Techne	317041<br>317041-C2<br>317041-C3
Mm-Pecam1	Bio-Techne	316721
Mm-Npr3	Bio-Techne	502991
Mm-Tcf21	Bio-Techne	508661-C2<br>508661-C3
Mm-Sema3d	Bio-Techne	488111
Mm-Fgf2	Bio-Techne	316851
Mm-Tgfb1	Bio-Techne	407751-C3
Mm-Tgfb2	Bio-Techne	406181
TSA plus FITC/Cy3/Cy5 (1:250/ 1:1000/ 1:1500, FISH)	Akoya Biosciences	NEL741001KT NEL752001KT
Mm-Wt1	ThermoFisher Scientific	VB4-13886-PF
Mm-Sulf1	ThermoFisher Scientific	VB4-3133182-PF<br>VB1-3048051-PF<br>VB6-3217745-PF
Mm-Sulf2	ThermoFisher Scientific	VB1-3038415-PF<br>VB6-3208199-PF
Mm-Tcf21	ThermoFisher Scientific	VB6-3197774-PF
Mm-Dpt	ThermoFisher Scientific	VB1-3034260-PF
Mm-Rgs5	ThermoFisher Scientific	VB4-3116783-PF
Mm-Npr3	ThermoFisher Scientific	VB1-3031000-PF

Primers

Target gene	Sequence (5'->3')	Amplicon size (bp)
Mm Gapdh	Forward: AGGTCGGTGTGAACGGATTTG<br>Reverse: TGTAGACCATGTAGTTGAGGTCA	123
Mm Actb	1. Forward: CTGTCGAGTCGCGTCCACC<br>1. Reverse: GCTTTGCACATGCCGGAGC<br>and<br>2. Forward: CATTGCTGACAGGATGCAGAAGG<br>2. Reverse: TGCTGGAAGGTGGACAGTGAGG	1. 135<br>and<br>2. 138
Mm Hprt1	Forward: TCAGTCAACGGGGGACATAAA<br>Reverse: GGGGCTGTACTGCTTAACCAG	142
Mm Sulf1	1. Forward: ACACACAGTAGAACGGAGCA<br>1. Reverse: CTGTGCGGGTCATAGTTTCCT<br>and<br>2. Forward: GGTCAAGGAACTGTGCCAGCAA<br>2. Reverse: AGGTTGCGTGCATTCTGACGGA	1. 144<br>and<br>2. 156
Mm Sulf2	Forward: CGTCTCGCTCCTCCATTCTC<br>Reverse: AAGAAAGCTGTCCGGTAGCC	154
Mm Wt1	Forward: TTCAAGGACTGCGAGAGAAG<br>Reverse: GGGAAAACTTTCGCTGACAA	112
Mm Sulf1 variant 1	Forward: TCCAAGTGAGAGTTGCAGGC<br>Reverse: GGCCCTCAGCACCTGAAAA	108
Mm Sulf1 variant 2	Forward: AGGAGGAGGAAGGAAGTCCC<br>Reverse: TGGCCCTCAGCACAAAAGTC	114
Mm Sulf1 variant 3	Forward: ACGCAGTATTTTCAGGACTCCAT<br>Reverse: CTCCTTGGTCGGCTGAGTTC	114
Mm Upk3b	Forward: GTGCTTCGGGTGGGCAATGATT<br>Reverse: TGGACCACTTCGTCTCAGCCTT	139

Recombinant DNA

Name	Vendor/Source	Repository URL
Custom vector pRP[En]-{Sulf1_Enh_12715347_5982_WT_SL}: miniCMV>Luciferase (VB230713-1205ffr)	VectorBuilder; This paper	https://github.com/RedpathAN/Epicardium_WT1_Sulf1_Regulatory_Interaction
Custom vector pRP[En]-{Sulf1_Enh_12715347_WT1_MUT5_SL}: miniCMV>Luciferase (VB230713-1207huq)	VectorBuilder; This paper	https://github.com/RedpathAN/Epicardium_WT1_Sulf1_Regulatory_Interaction

Cultured Cells

Name	Vendor/Source	Catalog # / Identifier
MEC1 Mouse Embryonic Epicardial Cell Line	Sigma-Aldrich	Cat# SCC187<br>RRID:CVCL_VR92

Recombinant Proteins

Name	Vendor/Source	Catalog #
Recombinant Human TGF-β1 (CHO derived)	PeproTech	100-21C
Recombinant Human TGF-β2 (HEK293 derived)	PeproTech	100-35B
Recombinant Human FGF-basic (146 a.a.)	PeproTech	100-18C
Recombinant Human PDGF-BB	PeproTech	100-14B
Recombinant Human Midkine	PeproTech	450-16
Recombinant Human Pleiotrophin	PeproTech	450-15
Recombinant Human/Mouse Wnt-5a	Bio-Techne	645-WN-010
Recombinant Mouse VEGF-B 167	Bio-Techne	2595-VE-025
Recombinant Human IGF-I	PeproTech	100-11
Recombinant Human R-Spondin-3	PeproTech	120-44
Recombinant Human BMP-10	PeproTech	120-40
Recombinant Human IGF-BP4	PeproTech	350-05B
Recombinant Human/Murine/Rat BMP-2 (CHO derived)	PeproTech	120-02C

Data & Code Availability

Description	Source/ Repository	Accession
scRNA-seq E10.5 heart	Gene Expression Omnibus	GSE76118
scRNA-seq E13.5 heart	Gene Expression Omnibus	GSE205797
scRNA-seq E11.5 heart	Gene Expression Omnibus	GSE193746
scRNA-seq E17.5 heart	This paper; Gene Expression Omnibus	GSE299996
ATAC-seq E13.5 epicardial cells	This paper; Gene Expression Omnibus	GSE299995
scRNA-seq FACS-sorted CD31+/tdTomato+ and CD31+/tdTomato-	Gene Expression Omnibus	GSE213274
scRNA-seq E10.5, E11.5, E13.5, and E17.5 C57BL/6 and E14.5 $Wt1^{GFPCre/GFPCre}$ hearts	Gene Expression Omnibus	GSE193346
CUT&RUN-seq MEC1 epicardial cell line	This paper; Gene Expression Omnibus	GSE299998
RNA-seq MEC1 epicardial cell line (control)	Gene Expression Omnibus	GSE313583
Scripts and custom pipelines	Github	https://github.com/RedpathAN/Epicardium_WT1_Sulf1_Regulatory_Interaction

Software & Algorithms

Description	Vendor/Source	Identifier URL
BD FACSDiva	BD Biosciences	https://www.bdbiosciences.com RRID:SCR_001456
FlowJo v10	FlowJo LLC	https://www.flowjo.com RRID:SCR_008520
CellProfiler v4.2.6	Broad Institute of MIT and Harvard	https://cellprofiler.org/ RRID:SCR_007358
ImageJ 1.54f/Fiji	Rasband and U. S. National Institutes of Health	https://imagej.net/ RRID:SCR_003070

cellranger	10x Genomics	https://support.10xgenomics.com/single-cell-gene-expression/software/ RRID:SCR_023221
Seurat v5.0.1	-	https://github.com/satijalab/seurat RRID:SCR_016341
CellChat v1.6.1	-	https://github.com/sqjin/CellChat RRID:SCR_021946
R v 4.3.2	The R Foundation	https://www.r-project.org RRID:SCR_001905
RStudio	Posit Software	https://posit.co RRID:SCR_000432
nf-core/cutandrun v2.0.0	https://doi.org/10.5281/zenodo.6624266	https://github.com/nf-core/cutandrun/tree/2.0
IGV v2.17.4	-	https://igv.org/ RRID:SCR_011793
Scanpy v1.9.5	-	https://github.com/theislab/scanpy RRID:SCR_018139
scGen v2.1.0	-	https://github.com/theislab/scgen
Prism 10.2.3	Graphpad	https://www.graphpad.com/ RRID:SCR_002798
ARTool v0.11.2	-	https://github.com/mjskay/artool RRID:SCR_027412

Other

Reagent/Resource	Vendor/Source	Catalog #
RNAscope® Multiplex Fluorescent Detection Kit v2	Bio-Techne	323110
Neonatal Heart Dissociation Kit, mouse and rat	Miltenyi Biotec	130-098-373
PrimeFlow RNA Assay Kit	ThermoFisher Scientific	88-18005-210
CUT&RUN Assay Kit	Cell Signaling Technology	86652
Qubit dsDNA HS Assay Kit-100 assays	ThermoFisher Scientific/ Life Technologies	Q32851
Bright-Glo Luciferase Assay System	Promega	E2610
Click-iT EdU Alexa Fluor 488 Imaging Kit-1 kit	ThermoFisher Scientific/ Life Technologies	C10337
EmbryoMAX®-DMEM with 4,500mg/L Glucose, 2.25g/L Sodium Bicarb and L-Glut, without Sodium Pyruvate	Sigma-Aldrich	SLM-120
EmbryoMax ES Cell Qualified Fetal Bovine Serum	Sigma-Aldrich	ES009-M
Silencer™ Select Negative Control No. 1 siRNA	ThermoFisher Scientific/ Life Technologies	4390843
Silencer® Select Wt1 siRNA - s76112	ThermoFisher Scientific/ Life Technologies	Assay ID: s76112
Silencer® Select Sulf1 siRNA - s109342	ThermoFisher Scientific/ Life Technologies	Assay ID: s109342
Silencer® Select Sulf1 siRNA - s109341	ThermoFisher Scientific/ Life Technologies	Assay ID: s109341
Lipofectamine RNAiMAX Transfection Reagent	ThermoFisher Scientific/ Life Technologies	13778075
Lipofectamine 3000 Transfection Reagent	ThermoFisher Scientific/ Life Technologies	L3000001

Illumina Tagment DNA Enzyme and Buffer Small Kit	Illumina	20034197
NEBNext(R) Ultra(TM) II Q5(R) Master Mix	New England Biolabs	M0544S
NEBNext(R) Ultra(TM) II DNA Library Prep Kit for Illumina(R)	New England Biolabs	E7645S
NEBNext(R) Multiplex Oligos for Illumina(R) (Index Primers Set 1)	New England Biolabs	E7335S
NEBNext(R) Multiplex Oligos for Illumina(R) (Index Primers Set 2)	New England Biolabs	E7500S
NEBNext(R) Multiplex Oligos for Illumina(R) (Index Primers Set 3)	New England Biolabs	E7710S
KAPA Library Quanitification Kits - Complete Kit (ROX Low)	Roche	07960336001
ImageXpress Pico Automated Cell Imaging System	Molecular Devices	-
Cell Staining Buffer (FC)	Biolegend	420201
SPRIselect	Beckman Coulter	B23317

ARRIVE GUIDELINES

The ARRIVE guidelines (https://arriveguidelines.org/) are a checklist of recommendations to improve the reporting of research involving animals. Key elements of study design should be included below to better enable readers to scrutinize the research adequately, evaluate its methodological rigor, and reproduce the methods or findings.

Study Design

Details of animals used, sex (when relevant), age, and groups are included in the manuscript text, figures, and supplementary files. For embryos, the sex was not determined as was not considered a relevant biological variable. In timed mating experiments, on average we observed 40-50% mating success. Control groups compared to WT1 KO animals (Wt1CreERT2/ CreERT2) included littermate Wt1CreERT2 hets or wild-types (Wt1+/+). Control groups compared to Sulf1 iKO animals (Sulf1fl/fl; Wt1CreERT2/+) included littermate Sulf1fl/fl; Wt1+/+ (Cre-). When lineage tracing was required Sulf1+/+Wt1CreERT2/+ from independent matings were used, such as in Figure S4. Single animal numbers are included in legends, and when pooled samples were used this was detailed in the methods and legends. Litter numbers where relevant were included in the legends.

Sample size

Sample size was not determined a priori, due to the nature of developmental studies, and the lack of prior, comparable studies to inform effect size and variability. The number of mice used in this study reflects the minimum number deemed sufficient to detect biologically relevant effects within the expected range of variability, based on previous experimental experience and commonly accepted standards in the field.

Sample sizes were predominantly determined by litter sizes, and the minimum litter number of 2 per group. At least 3 individual animal units were used to represent a specific group, and often a minimum of 4 individual units per group was used when statistical analysis was performed to compare between control and KO genotypes. Exact number of individual animals analysed per group is stated in figure legends, and when pooling was required, this number was stated in the figure legends and methods. Individual data points on plots also indicate individual biological replicates.

Inclusion Criteria

Embryos were included in the study if they were the correct stage and genotype.

Exclusion Criteria

Embryos excluded from analysis includes those that encompassed major developmental defects or resorption due to reasons unrelated to genotype, for example, coming from litters that demonstrated significant resorption, developmental delay and death attributed to tamoxifen toxicity. If an entire litter was identified as incorrect developmental stage, due to biological/technical issues e.g. incorrect plug timing. Embryonic hearts that suffered mechanical damage or came from embryos that were dead upon dissection. In downstream experimental processes, technical failures such as tissue explant non-adhesion or lifting, and insufficient growth resulted in exclusion of these samples.

Figure 3F-3G: explants excluded from analysis if very limited growth and too few cells compared to littermates of comparable genotype. <3 control and 1 Wt1 KO explants were excluded.

Individual animals per group are stated in the figure legends. When pooling required, this number is stated in the figure legends and methods. Individual data points on plots also indicate individual biological replicates.

Randomization

Randomization is not applicable, due to the nature of the studies. Litter sizes naturally vary in number, and genotype frequencies may deviate from expected Mendelian ratios. Exact numbers and genotypes are only confirmed after samples are harvested. Randomisation is not applicable in this case as groups are determined by genotypes (control and KO). All downstream analyses were performed on embryos based on dam tamoxifen treatment group, embryonic stage (age) and genotype.

Blinding

Investigators practiced blinding where possible, with animals assigned litter and embryo identification codes at harvest. Tissues were allocated to groups (control vs KO) prior to processing e.g. staining, to ensure sufficient samples investigated. Identification codes instead of genotypes or group names were used to label samples during experimental processes, data collection and analysis.

Experimental animals

Species, strain and developmental age stated in text, methods and figure legends. Weight of dams used to calculate tamoxifen volume, which varied according to litter size and dam age. Age of dams varied, typically 16-24 weeks, with maximum of 38 weeks of age. Age of experimental samples were fixed to embryonic stage and always stated. Healthy animals with

normal immune function were used. Genotypes are provided in text and methods sections. Previous procedures (to dams only) included e.g. earclipping for identification/genotyping.

Further details found in the ARRIVE guidelines 2.0: author checklist

Table S9 – Statistical approaches

Figure	Normalisation procedures	Normal distribution? (Shapiro-Wilks)	Normality of residuals?	Equal SDs	Sample size	Statistical Test	Pairwise comparisons	Post-hoc	Adjusted p-value reported	NOTES
**MAIN FIGURES**										
1G	N/A	Yes	Yes	Yes	≥6 (6-8)	one-way ANOVA	Preselected pairs	Sidak	Yes	
1H	Visual - no statistical test required									
2C	N/A	Yes	Yes	Yes	<6 (5)	Mann-Whitney U	N/A	N/A	N/A	
2H	N/A	Yes	Yes	Yes	<6 (3)	Multi Mann-Whitney	N/A	Holm-Sidak	Yes	
2J	N/A	Yes	Yes	Yes	<6 (3)	Multi Mann-Whitney	N/A	Holm-Sidak	Yes	
3F	N/A	Yes. Lognormality passed except Upk3b for 1 group. Data log-transformed to normalize distributions.	Yes	Yes	<6 (4-10) after separating A/V	Mann-Whitney U	N/A	N/A	N/A	Atrial/ventricular explants analysed separately, as independent samples, no cross-comparison. No multiple comparison correction required.
3G	N/A	Yes, except 1 group (bin 2, Wt1KO_V; Sulf1).	No	No	<6 (4-10) after separating A/V	Multi Mann-Whitney	Genotypes for each clustered bin	Holm-Sidak	Yes	Comparisons and P values summarised in Table S1.
3M	N/A	Yes	Yes	Yes	<6 (4-5)	Mann-Whitney U	N/A	N/A	N/A	
3N	N/A	Yes	Yes	No, SD larger in KO. >2x	<6 (4-5)	Mann-Whitney U	N/A	N/A	N/A	
4F'	N/A	No, 1 group failed per gene	No	No, SD larger in 1 group. >2x	≥6 (6)	Mann-Whitney U	N/A	N/A	N/A	
4I (Area)	N/A	Yes	Yes	Yes	<6 (5)	Kruskal-Wallis	Each against control	Dunn	Yes	
4I (CTCF)	N/A	Yes	Yes	Yes	<6 (5)	Kruskal-Wallis	Each against control	Dunn	Yes	

4K (Area)	N/A	Yes	Yes	No, SD larger in control group, due to outlier. >2x	<6 (5)	Kruskal-Wallis	Each against control	Dunn	Yes	
4K (CTCF)	N/A	Yes	Yes	No, SD larger in treatment groups. >2x	<6 (5)	Kruskal-Wallis	Each against control	Dunn	Yes	
5G	Transformed log(Y+0.5), to correct -ve values. Lognormal	Yes. Lognormality appropriate transformation for luminescence.	Yes	Yes	≥6 (6)	Lognormal ordinary one-way ANOVA	Preselected pairs	Sidak	Yes	
7C	N/A	Yes	No	Yes	≥6 (6-9)	two-way ANOVA	Preselected pairs	Sidak	Yes	
7F	N/A	Yes, except 2 groups. All passed Lognormality test.	No	No	><6 (4-9)	ART ANOVA	Preselected pairs (against VEH per KD condition)	Holm-Sidak	Yes. Only significant values in text to keep heatmap legible	Comparisons and P values summarised in Table S2.
7H	Logit(Y/100) transformation of percentages to meet ANOVA criteria. N/A for MES SUM	Yes	Most subsets	Yes	>6 (7-9)	two-way ANOVA	Preselected pairs	BKY (for Mes Sum) and BKY (for subsets)	Yes	Comparisons and Q values summarised in Table S3.
7K	N/A	Yes	No	Yes	<6 (3)	ART ANOVA (RQ)<br>Kruskal-Wallis (AUC)	Preselected pairs (RQ)<br>Each against control (AUC)	BKY (RQ)<br>Dunn (AUC)	Yes (RQ). Only significant values in text to keep line graph legible.<br>Yes (AUC)	Comparisons and Q values summarised in Table S6. Best fit (non-linear regression) and AUC analyses summarised in Table S8 and individual AUC values plotted in Figure 7K

**SUPPLEMENTAL FIGURES**										
S3G	N/A	Yes	Yes	Yes	<6 (3)	Mann-Whitney U	N/A	N/A	N/A	
S6D	N/A	Yes	Yes	Equal variance cannot be assumed for pooled samples of unequal pooling = unequal variance.	<6 (3-4)	Mann-Whitney U	N/A	N/A	N/A	
S7F	N/A	N/A	N/A	N/A	>6 (11-28)	Fisher's exact test	N/A	N/A	N/A	
S7G	N/A	Yes	N/A	No, >2x SD in 1 KO population. Equal variance cannot be assumed for pooled samples of unequal pooling = unequal variance.	<6 (3-4)	Multi Mann-Whitney	N/A	Holm-Sidak	Yes	
S7J	N/A	Yes	N/A	No, >2x SD in 1 KO population. Equal variance cannot be assumed for pooled samples of unequal pooling = unequal variance.	<6 (3-4)	Multi Mann-Whitney	N/A	Holm-Sidak	Yes	
S8E	N/A	Yes	Yes	Yes, explant level analysis - Use Welch for stringency	≥6 (6 explants)	Unpaired Welch t test	N/A	N/A	N/A	

S8G	N/A	Yes, except siWt1 group. All passed Lognormality test.	No	Yes, explant level analysis - Use Welch for stringency. Lognormality affects variance.	>6 (11-22 explants)	Multiple Unpaired Welch t test	N/A	Holm-Sidak	Yes	
S8H	Visual - no statistical test required									
S8J	N/A	No, most failed. siCo group failed Lognormality test.	No	No	>6 (13-19 explants)	Multi Mann-Whitney	N/A	Holm-Sidak	Yes	
S9I	N/A	Yes	Yes	N/A	<6 (4)	Friedman test	All groups	Dunn	Yes	
S9J	N/A	Yes	Yes	No, SD larger in 1 group. >2x	<6 (4)	Mann-Whitney U	N/A	N/A	N/A	
S10B	Transformed log(Y+0.5), to correct -ve values. Lognormal	Yes. Lognormality appropriate transformation for luminescence.	Yes	Yes	>6 (7)	Lognormal ordinary one-way ANOVA	Preselected pairs	Sidak	Yes	
S11C	N/A	Yes, except 2 groups (TGFB2+siCo, BMP10+siCo).	Yes	Yes	><6 (4-11)	ART ANOVA	Preselected pairs (against VEH per KD condition, and siCo per KD condition).	Holm-Sidak	Yes. Only significant values and p<0.25 annotated in figure to keep heatmap legible	Comparisons and P values summarised in Table S7.
S11D	N/A	Yes	No	Yes	>6 (7-9)	two-way ANOVA	Preselected pairs	Fisher's LSD	No (individual p)	Do not correct for multiple comparison as prior design to only test if KD+ GF combination enhances EMT, 1-direction

Note: For analyses of groups with 6 ≤ n <10 that do not show significant departures from normality, and parametric tests were used, we acknowledge that violations of the assumptions of the test may not have been detected due to low power and note it as a limitation of this study. Further, we provide Shapiro-Wilk test for normality results in Table S10.

Table S10 – Shapiro-Wilk test for normality results

Fig	Normal distribution	Shapiro-Wilks test results																				
**MAIN FIGURES**																						
1G	Yes	Group	E11.5 (Sulf1)	E13.5 (Sulf1)	E17.5 (Sulf1)	E11.5 (Sulf2)	E13.5 (Sulf2)	E17.5 (Sulf2)														
		P value	0.33	0.14	0.52	0.50	0.14	0.34														
2C	Yes	Group	epi	epdc																		
		P value	0.78	0.88																		
2H	Yes	Group	Tomato+ (Epi)	Tomato+ (Mes)	Tomato- (Mes)	Tomato+ (Mural)	Tomato- (Mural)															
		P value	0.50	0.73	0.26	0.76	0.58															
2J	Yes	Group	Tomato+ (Epi)	Tomato+ (Mes)	Tomato- (Mes)	Tomato+ (Mural)	Tomato- (Mural)															
		P value	0.92	0.46	0.33	0.91	0.75															
3F	Yes. Lognormality passed except Upk3b for 1 group. Data log-transformed to normalize distributions.		Sulf1				Sulf2				Upk3b											
		Group	Ctrl V	Wt1KO V	Ctrl A	Wt1KO A	Ctrl V	Wt1KO V	Ctrl A	Wt1KO A	Ctrl V	Wt1KO V	Ctrl A	Wt1KO A								
		P value	0.62	0.20	0.26	0.83	0.81	0.29	0.38	0.49	0.31	0.084	0.047	0.31								
3G	Yes, except 1 group (bin 2, Wt1KO_V).		Ctrl V (Bin)					Wt1KO V (Bin)					Ctrl A (Bin)					Wt1KO A (Bin)				
		Group	0	1	2	3	4	0	1	2	3	4	0	1	2	3	4	0	1	2	3	4
		P value	0.14	0.29	0.16	0.28	0.72	0.87	0.99	0.01	0.97	0.09	0.19	0.87	0.70	0.29	0.22	0.72	0.45	0.26	0.89	0.50
3M	Yes	Group	Control	Sulf1 iKO																		
		P value	0.98	0.055																		
3N	Yes	Group	Control	Sulf1 iKO																		
		P value	0.72	0.33																		
4F'	No, 1 group failed per gene	Group	siCo (Sulf1)	siWt1 (Sulf1)	siCo (Sulf2)	siWt1 (Sulf2)																
		P value	0.063	0.039	0.003	0.18																

<table>
<tr><td>4I</td><td>Yes</td><td>
<table>
<tr><td></td><td colspan="3">Area</td><td colspan="3">CTCF</td></tr>
<tr><td>Group</td><td>siControl</td><td>siWt1</td><td>siSulf1</td><td>siControl</td><td>siWt1</td><td>siSulf1</td></tr>
<tr><td>P value</td><td>0.34</td><td>0.71</td><td>0.88</td><td>0.091</td><td>0.37</td><td>0.93</td></tr>
</table>
</td></tr>
<tr><td>4K</td><td>Yes</td><td>
<table>
<tr><td></td><td colspan="3">Area</td><td colspan="3">CTCF</td></tr>
<tr><td>Group</td><td>siControl</td><td>siWt1</td><td>siSulf1</td><td>siControl</td><td>siWt1</td><td>siSulf1</td></tr>
<tr><td>P value</td><td>0.44</td><td>0.43</td><td>0.20</td><td>0.089</td><td>0.80</td><td>0.73</td></tr>
</table>
</td></tr>
<tr><td>5G</td><td>Yes. Lognormality appropriate transformation for luminescence.</td><td>
<table>
<tr><td></td><td colspan="2">siCo</td><td colspan="3">siWt1</td></tr>
<tr><td>Group</td><td>mCMVp_E1</td><td>mCMVp_E1mut5</td><td>VEH</td><td>mCMVp_E1</td><td>mCMVp_E1mut5</td></tr>
<tr><td>P value</td><td>0.88</td><td>0.89</td><td>0.25</td><td>0.86</td><td>0.14</td></tr>
</table>
</td></tr>
<tr><td>7C</td><td>Yes</td><td>
<table>
<tr><td></td><td colspan="3">FGF2</td><td colspan="3">PDGF</td><td colspan="3">TGFβ1</td><td colspan="3">TGFβ2</td></tr>
<tr><td>Group</td><td>siCo</td><td>siWt1</td><td>siSulf1</td><td>siCo</td><td>siWt1</td><td>siSulf1</td><td>siCo</td><td>siWt1</td><td>siSulf1</td><td>siCo</td><td>siWt1</td><td>siSulf1</td></tr>
<tr><td>P value</td><td>0.82</td><td>0.50</td><td>0.17</td><td>0.53</td><td>0.21</td><td>0.96</td><td>0.94</td><td>0.20</td><td>0.96</td><td>0.35</td><td>0.72</td><td>0.50</td></tr>
</table>
</td></tr>
<tr><td>7F</td><td>Yes, except 2 groups. All passed Lognormality test.</td><td>
<table>
<tr><td></td><td colspan="14">siCo</td></tr>
<tr><td>Group</td><td>VEH</td><td>MK</td><td>PTN</td><td>VEGFB</td><td>IGFBP4</td><td>IGF1</td><td>WNT5A</td><td>RSPO3</td><td>TGFB1</td><td>TGFB2</td><td>PDGFBB</td><td>BMP10</td><td>FGF2</td><td>BMP2</td></tr>
<tr><td>P value</td><td>0.056</td><td>0.98</td><td>0.46</td><td>0.95</td><td>0.16</td><td>0.89</td><td>0.60</td><td>0.93</td><td>0.81</td><td>0.52</td><td>0.077</td><td>0.41</td><td>0.90</td><td>0.50</td></tr>
<tr><td></td><td colspan="14">siWt1</td></tr>
<tr><td>Group</td><td>VEH</td><td>MK</td><td>PTN</td><td>VEGFB</td><td>IGFBP4</td><td>IGF1</td><td>WNT5A</td><td>RSPO3</td><td>TGFB1</td><td>TGFB2</td><td>PDGFBB</td><td>BMP10</td><td>FGF2</td><td>BMP2</td></tr>
<tr><td>P value</td><td>0.42</td><td>0.91</td><td>0.14</td><td>0.10</td><td>0.96</td><td>0.93</td><td>0.50</td><td>0.36</td><td>0.21</td><td>0.41</td><td>0.37</td><td>0.19</td><td>0.96</td><td>0.99</td></tr>
<tr><td></td><td colspan="14">siSulf1</td></tr>
<tr><td>Group</td><td>VEH</td><td>MK</td><td>PTN</td><td>VEGFB</td><td>IGFBP4</td><td>IGF1</td><td>WNT5A</td><td>RSPO3</td><td>TGFB1</td><td>TGFB2</td><td>PDGFBB</td><td>BMP10</td><td>FGF2</td><td>BMP2</td></tr>
<tr><td>P value</td><td>0.43</td><td>0.54</td><td>0.17</td><td>0.24</td><td>0.42</td><td>0.75</td><td>0.57</td><td>0.22</td><td>0.29</td><td>0.12</td><td>0.81</td><td>0.55</td><td>0.72</td><td>0.47</td></tr>
</table>
</td></tr>
</table>

<table>
<tr><td>7H</td><td>Yes</td><td>
<table>
<tr><td colspan="6">siControl</td></tr>
<tr><td>Group</td><td>VEH</td><td>TGFB1</td><td>TGFB1+FGF2</td><td>TGFB1+ PDGFBB</td><td>TGFB1 +IGF1</td></tr>
<tr><td>P value</td><td>0.054</td><td>0.47</td><td>0.87</td><td>0.19</td><td>0.26</td></tr>
<tr><td colspan="6">siWt1</td></tr>
<tr><td>Group</td><td>VEH</td><td>TGFB1</td><td>TGFB1+FGF2</td><td>TGFB1+ PDGFBB</td><td>TGFB1 +IGF1</td></tr>
<tr><td>P value</td><td>0.47</td><td>0.11</td><td>0.20</td><td>0.66</td><td>0.34</td></tr>
<tr><td colspan="6">siSulf1</td></tr>
<tr><td>Group</td><td>VEH</td><td>TGFB1</td><td>TGFB1+FGF2</td><td>TGFB1+ PDGFBB</td><td>TGFB1 +IGF1</td></tr>
<tr><td>P value</td><td>0.58</td><td>0.85</td><td>0.68</td><td>0.63</td><td>0.35</td></tr>
</table>
</td></tr>
<tr><td>7K</td><td>Yes</td><td>
<table>
<tr><td></td><td colspan="6">siControl</td><td colspan="6">siSulf1</td><td colspan="6">siWt1</td></tr>
<tr><td>Group</td><td>T15</td><td>T30</td><td>T60</td><td>T90</td><td>T120</td><td>T150</td><td>T15</td><td>T30</td><td>T60</td><td>T90</td><td>T120</td><td>T150</td><td>T15</td><td>T30</td><td>T60</td><td>T90</td><td>T120</td><td>T150</td></tr>
<tr><td>P value</td><td>0.92</td><td>0.31</td><td>0.12</td><td>0.14</td><td>0.12</td><td>0.90</td><td>0.50</td><td>0.37</td><td>0.75</td><td>0.19</td><td>0.84</td><td>0.98</td><td>0.25</td><td>0.25</td><td>0.11</td><td>0.06</td><td>0.16</td><td>0.14</td></tr>
</table>
</td></tr>
<tr><td colspan="3">SUPPLEMENTAL FIGURES</td></tr>
<tr><td>S3G</td><td>Yes</td><td>
<table>
<tr><td>Group</td><td>Atrial</td><td>Ventricular</td></tr>
<tr><td>P value</td><td>0.73</td><td>0.37</td></tr>
</table>
</td></tr>
<tr><td>S6D</td><td>Yes</td><td>
<table>
<tr><td>Group</td><td>WT</td><td>Het</td></tr>
<tr><td>P value</td><td>0.30</td><td>0.63</td></tr>
</table>
</td></tr>
<tr><td>S7G</td><td>Yes</td><td>
<table>
<tr><td>Group</td><td>Control (Epi Lin)</td><td>Sulf1 iKO (Epi Lin)</td><td>Control (Epi)</td><td>Sulf1 iKO (Epi)</td><td>Control (Mes)</td><td>Sulf1 iKO (Mes)</td><td>Control (Mural)</td><td>Sulf1 iKO (Mural)</td><td>Control (CEC)</td><td>Sulf1 iKO (CEC)</td><td>Control (Endo)</td><td>Sulf1 iKO (Endo)</td><td>Control (CM)</td><td>Sulf1 iKO (CM)</td></tr>
<tr><td>P value</td><td>0.39</td><td>0.46</td><td>0.46</td><td>0.20</td><td>0.29</td><td>0.60</td><td>0.21</td><td>0.75</td><td>0.92</td><td>0.15</td><td>0.58</td><td>0.76</td><td>0.26</td><td>0.62</td></tr>
</table>
</td></tr>
<tr><td>S7J</td><td>Yes</td><td>
<table>
<tr><td>Group</td><td>Control (Epi Lin)</td><td>Sulf1 iKO (Epi Lin)</td><td>Control (Wt1+Tcf21+)</td><td>Sulf1 iKO (Wt1+Tcf21+)</td><td>Control (Wt1+Tcf21lo/-)</td><td>Sulf1 iKO (Wt1+Tcf21lo/-)</td><td>Control (Wt1-Tcf21+)</td><td>Sulf1 iKO (Wt1-Tcf21+)</td><td>Control (Wt1-Tcf21-)</td><td>Sulf1 iKO (Wt1-Tcf21-)</td></tr>
<tr><td>P value</td><td>0.64</td><td>0.89</td><td>0.60</td><td>0.34</td><td>0.46</td><td>0.49</td><td>0.82</td><td>0.46</td><td>0.54</td><td>0.35</td></tr>
</table>
</td></tr>
<tr><td></td><td></td><td></td></tr>
</table>

<table>
<tr><td>S8E</td><td>Yes</td><td><table><tr><td>Group</td><td>siCo</td><td>siWt1</td></tr><tr><td>P value</td><td>0.31</td><td>0.52</td></tr></table></td></tr>
<tr><td>S8G</td><td>Yes, except siWt1 group. All passed Lognormality test.</td><td><table><tr><td></td><td colspan="4">Sulf1</td><td colspan="4">Sulf2</td><td colspan="4">Wt1</td></tr><tr><td>Group</td><td>siCo (prox)</td><td>siWt1 (prox)</td><td>siCo (dist)</td><td>siWt1 (dist)</td><td>siCo (prox)</td><td>siWt1 (prox)</td><td>siCo (dist)</td><td>siWt1 (dist)</td><td>siCo (prox)</td><td>siWt1 (prox)</td><td>siCo (dist)</td><td>siWt1 (dist)</td></tr><tr><td>P value</td><td>0.32</td><td>0.95</td><td>0.18</td><td>0.51</td><td>0.63</td><td>0.83</td><td>0.42</td><td>0.83</td><td>0.51</td><td>0.78</td><td>0.31</td><td>0.059</td></tr></table></td></tr>
<tr><td>S8J</td><td>No, most failed. siCo group failed Lognormality test.</td><td><table><tr><td>Group</td><td>siCo (prox)</td><td>siWt1 (prox)</td><td>siCo (dist)</td><td>siWt1 (dist)</td></tr><tr><td>P value</td><td>0.016</td><td>0.67</td><td>0.0013</td><td>0.55</td></tr></table></td></tr>
<tr><td>S9I</td><td>Yes</td><td><table><tr><td>Group</td><td>variant 1</td><td>variant 2</td><td>variant 3</td></tr><tr><td>P value</td><td>0.90</td><td>0.39</td><td>0.57</td></tr></table></td></tr>
<tr><td>S9J</td><td>Yes</td><td><table><tr><td></td><td colspan="2">Sulf1 variant 1</td><td colspan="2">Sulf1 variant 3</td></tr><tr><td>Group</td><td>siCo</td><td>siWt1</td><td>siCo</td><td>siWt1</td></tr><tr><td>P value</td><td>0.26</td><td>0.18</td><td>0.30</td><td>0.14</td></tr></table></td></tr>
<tr><td>S10B</td><td>Yes. Lognormality appropriate transformation for luminescence.</td><td><table><tr><td></td><td colspan="2">siControl</td><td colspan="3">siWt1</td></tr><tr><td>Group</td><td>mCMVp_E1</td><td>mCMVp_E1mut5</td><td>VEH</td><td>mCMVp_E1</td><td>mCMVp_E1mut5</td></tr><tr><td>P value</td><td>0.57</td><td>0.74</td><td>0.55</td><td>0.19</td><td>0.77</td></tr></table></td></tr>
<tr><td>S11C</td><td>Yes, except 2 groups (TGFβ2+siCo, BMP10+siCo)</td><td><table><tr><td>Group</td><td>VEH+siCo</td><td>VEH+siWt1</td><td>VEH+siSulf1</td><td>MK+siCo</td><td>MK+siWt1</td><td>MK+siSulf1</td></tr><tr><td>P value</td><td>0.21</td><td>0.47</td><td>0.32</td><td>0.75</td><td>0.74</td><td>0.55</td></tr><tr><td>Group</td><td>PTN+siCo</td><td>PTN+siWt1</td><td>PTN+siSulf1</td><td>VEGFB+siCo</td><td>VEGFB+siWt1</td><td>VEGFB+siSulf1</td></tr><tr><td>P value</td><td>0.26</td><td>0.42</td><td>0.59</td><td>0.19</td><td>0.23</td><td>0.88</td></tr><tr><td>Group</td><td>IGFBP4+siCo</td><td>IGFBP4+siWt1</td><td>IGFBP4+siSulf1</td><td>IGF1+siCo</td><td>IGF1+siWt1</td><td>IGF1+siSulf1</td></tr><tr><td>P value</td><td>0.73</td><td>0.69</td><td>0.24</td><td>0.11</td><td>0.87</td><td>0.14</td></tr></table></td></tr>
</table>

		Group	WNT5A+siCo	WNT5A+siWt1	WNT5A+siSulf1	RSPO3+siCo	RSPO3+siWt1	RSPO3+siSulf1						
		P value	0.76	0.19	0.40	0.58	0.47	0.52						
		Group	TGFβ1+siCo	TGFβ1+siWt1	TGFβ1+siSulf1	TGFβ2+siCo	TGFβ2+siWt1	TGFβ2+siSulf1						
		P value	0.32	0.91	0.26	0.0064	0.28	0.85						
		Group	PDGFBB+siCo	PDGFBB+siWt1	PDGFBB+siSulf1	BMP10+siCo	BMP10+siWt1	BMP10+siSulf1						
		P value	0.42	0.91	0.24	0.028	0.63	0.28						
		Group	FGF2+siCo	FGF2+siWt1	FGF2+siSulf1	BMP2+siCo	BMP2+siWt1	BMP2+siSulf1						
		P value	0.65	0.57	0.81	0.25	0.62	0.63						
			VEH			TGFβ1			TGFβ2			IGF1		
S11D	Yes	Group	siCo	siWt1	siSulf1	siCo	siWt1	siSulf1	siCo	siWt1	siSulf1	siCo	siWt1	siSulf1
		P value	0.056	0.42	0.43	0.81	0.21	0.29	0.52	0.41	0.12	0.89	0.93	0.75
