# Supplementary material for: Regulated HSPG Signaling Directs Epicardial Behavior to Support Cardiac Formation
Source: Circ Res. 2026 Aug 25;139(8):e327330. doi: 10.1161/CIRCRESAHA.125.327330 (PMC13608351; doi:10.1161/CIRCRESAHA.125.327330)

Set 1

Optimal exposure - unedited Image used for quantification

Superimposed with ladder for annotation

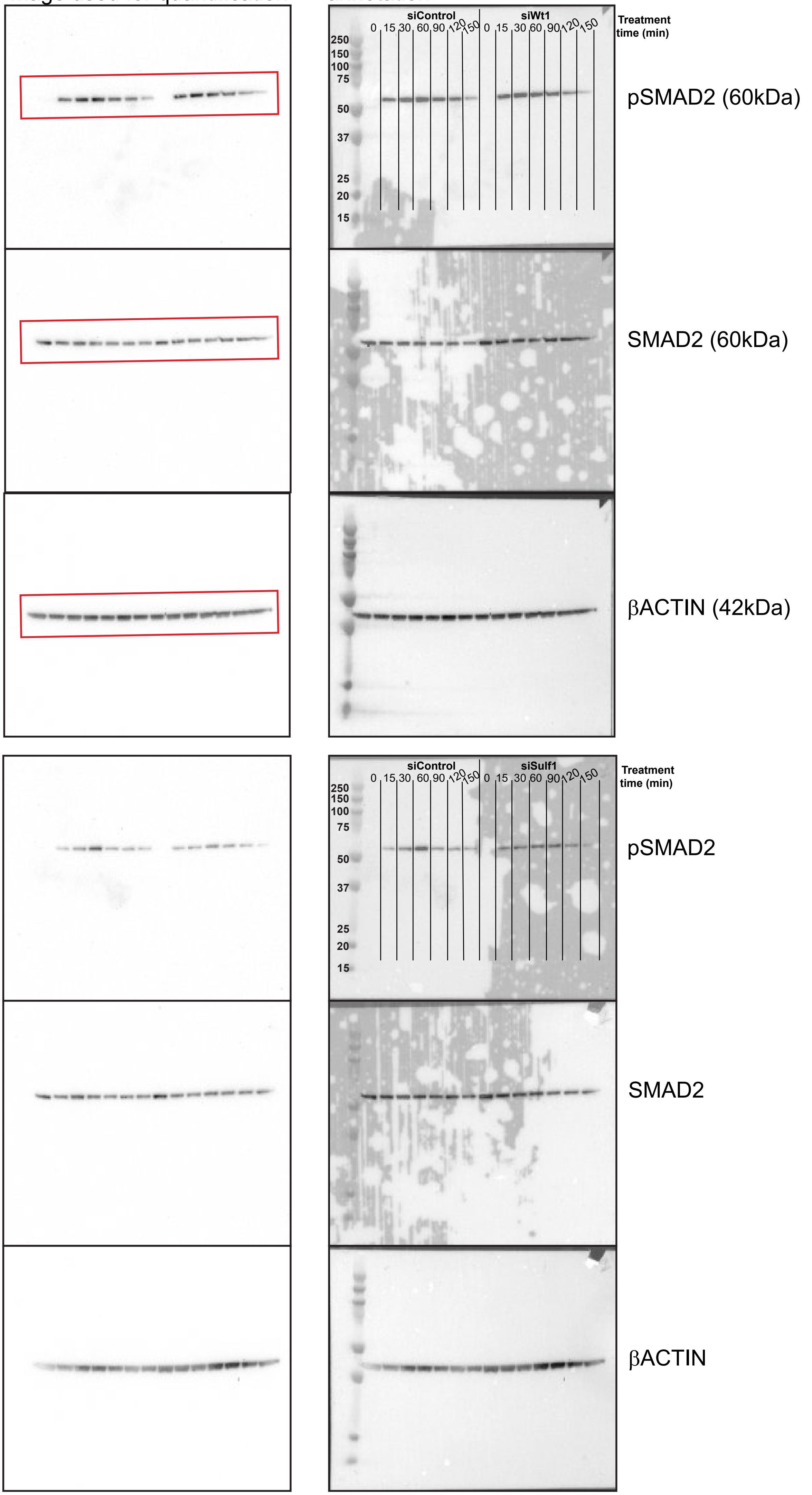

Set 2

Optimal exposure - unedited Image used for quantification

Superimposed with ladder for annotation

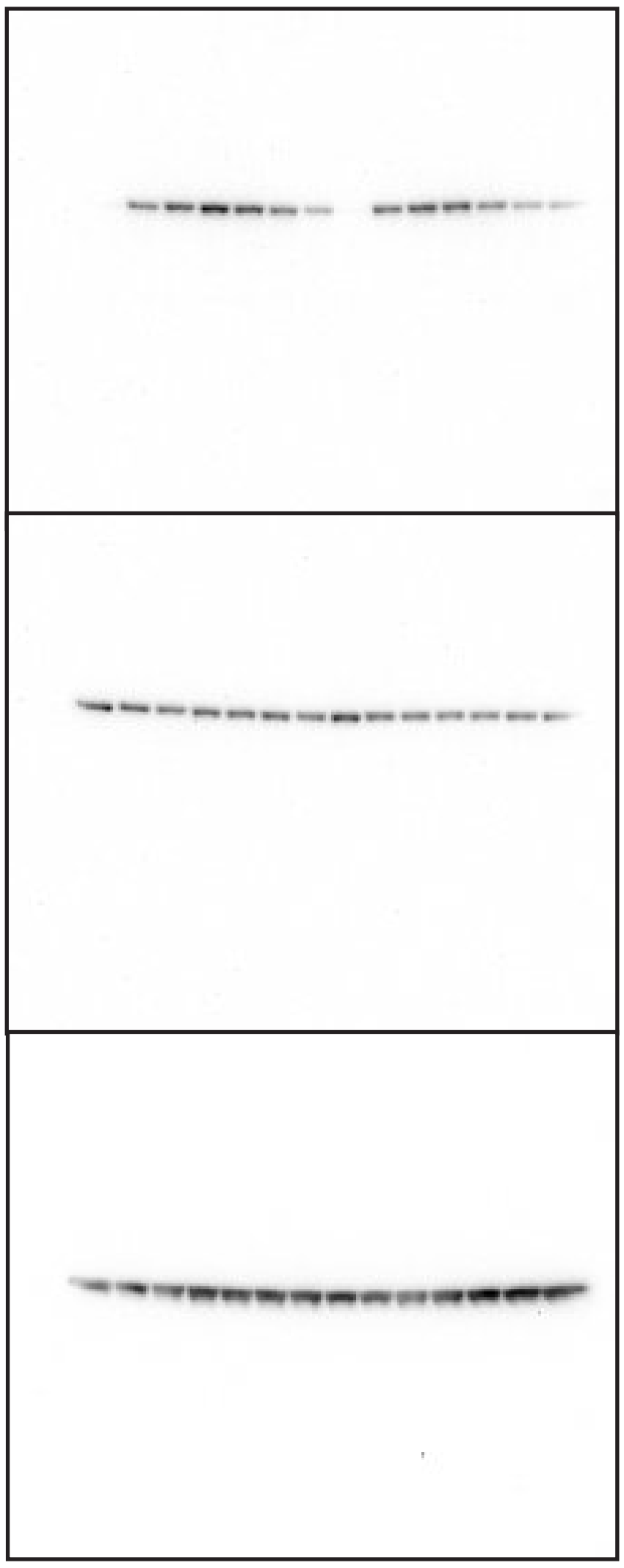

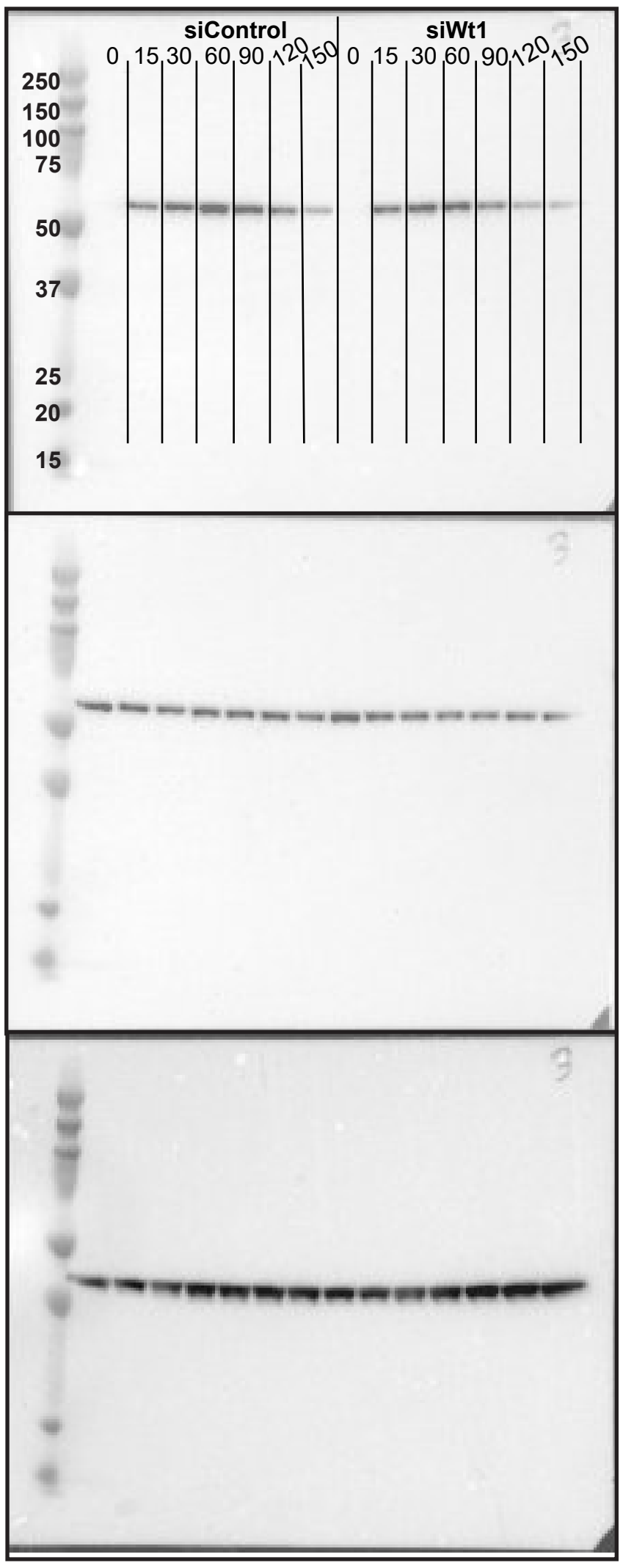

Treatment time (min)

pSMAD2

SMAD2

βACTIN

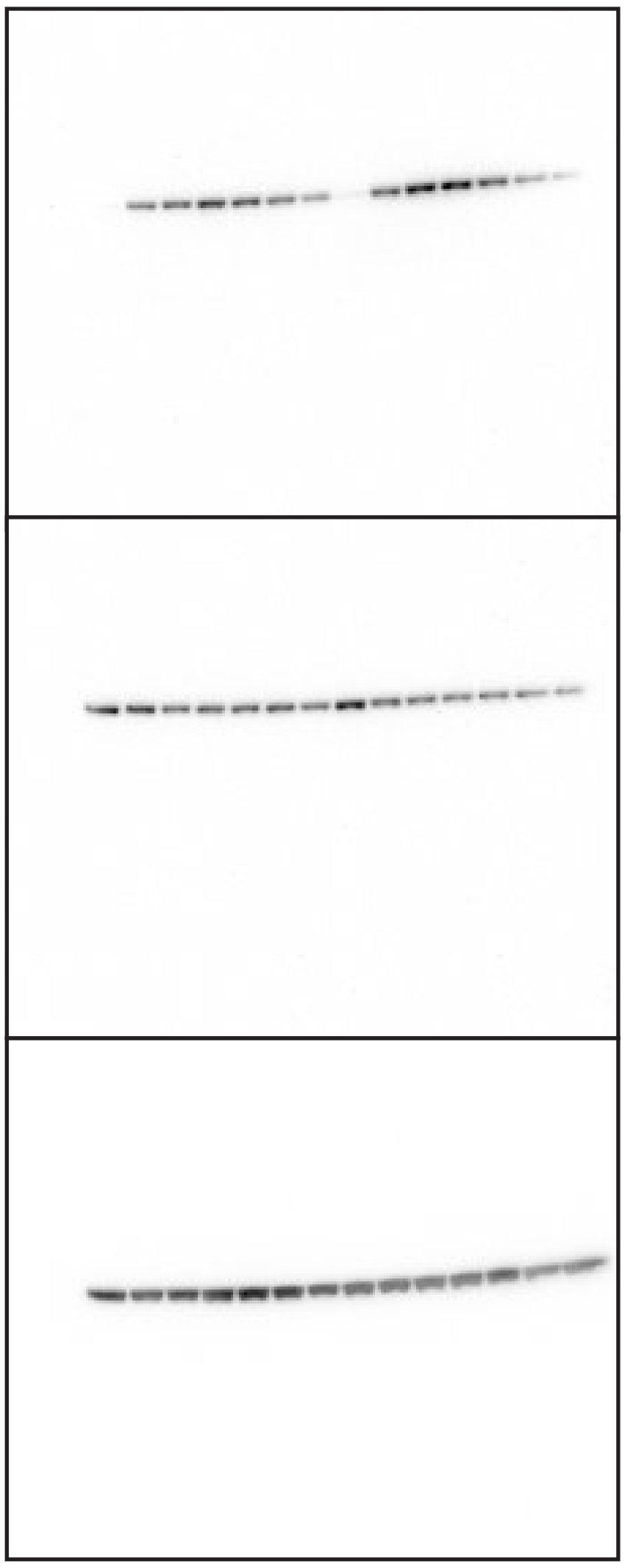

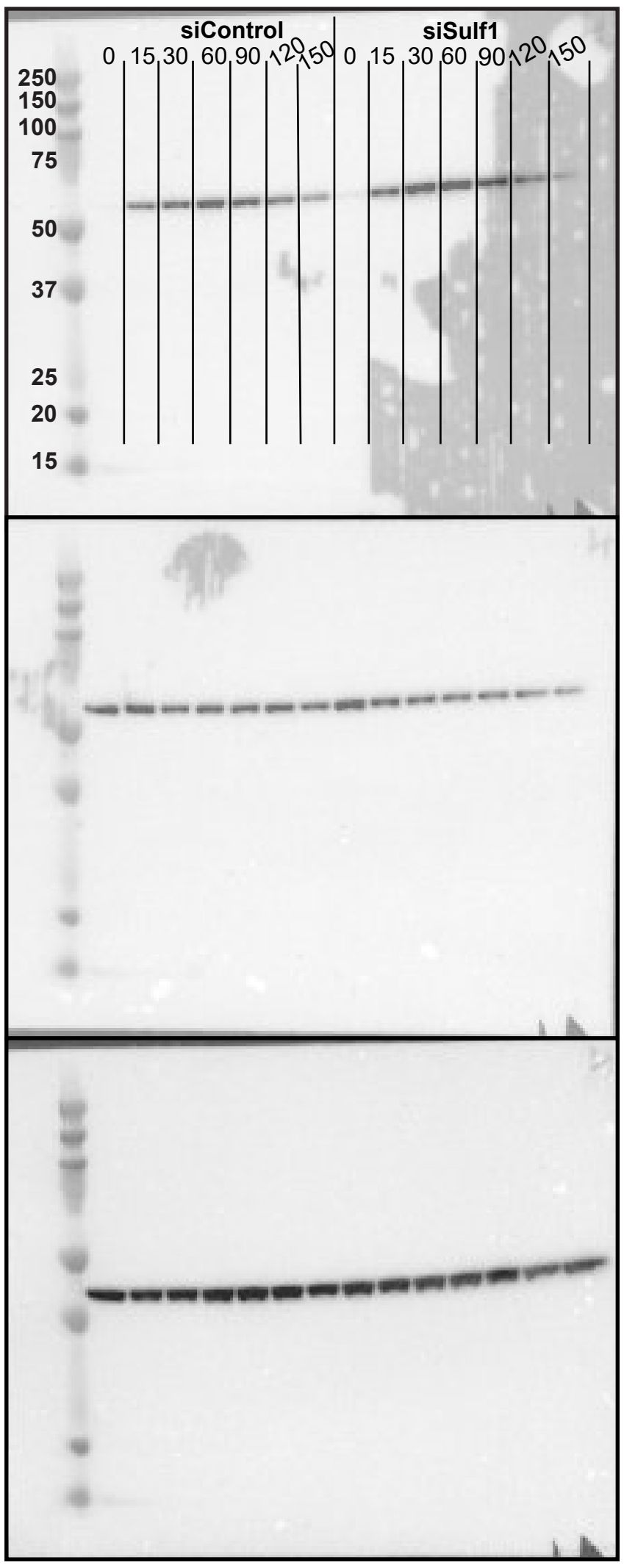

Treatment time (min)

pSMAD2

SMAD2

βACTIN

Set 3

Optimal exposure - unedited Image used for quantification

Superimposed with ladder for annotation

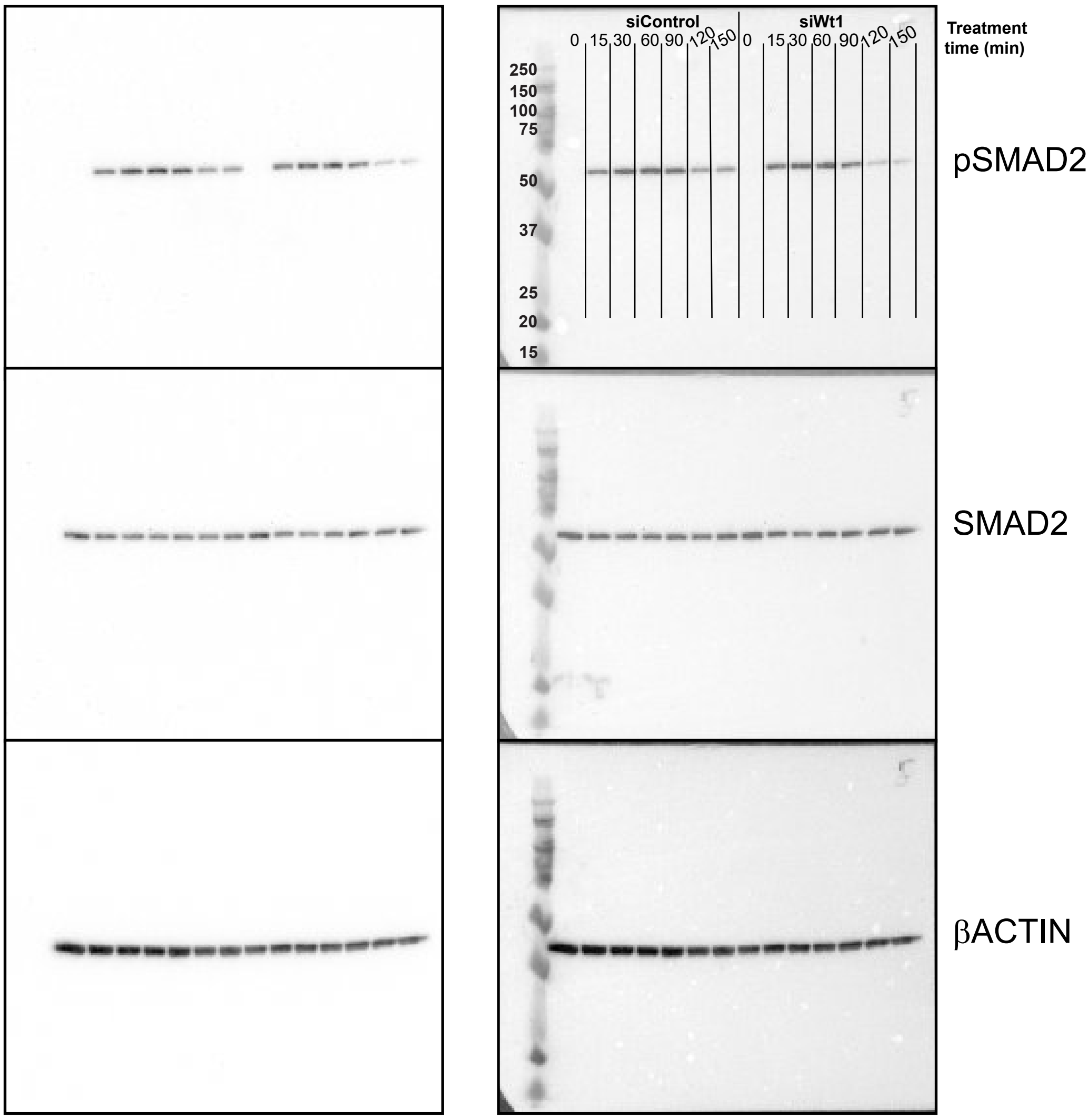

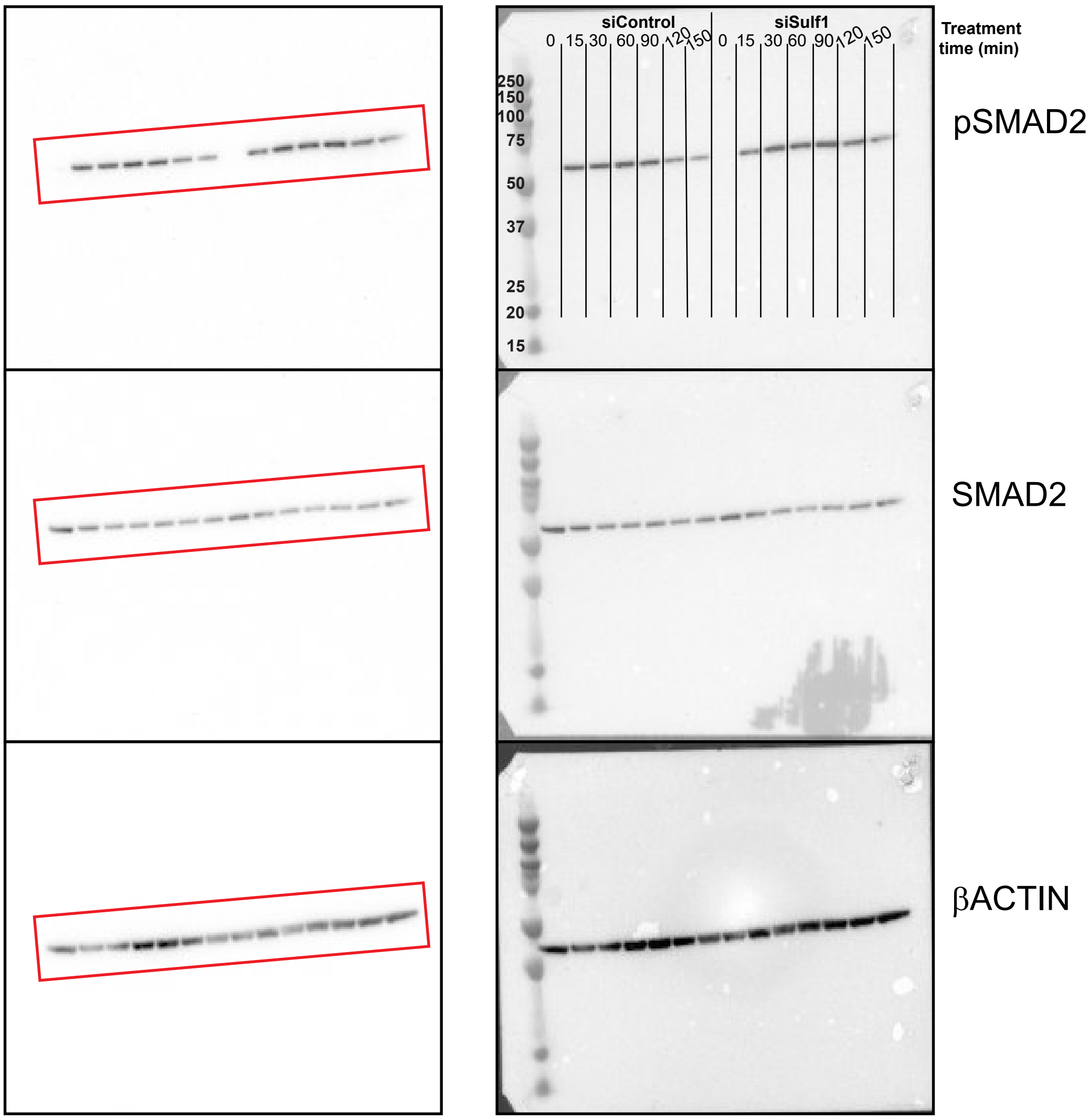

Set 4

Optimal exposure - unedited
Image used for quantification

Superimposed with ladder for
annotation

X = technical error
missing TGF stimulus
sample excluded from analysis

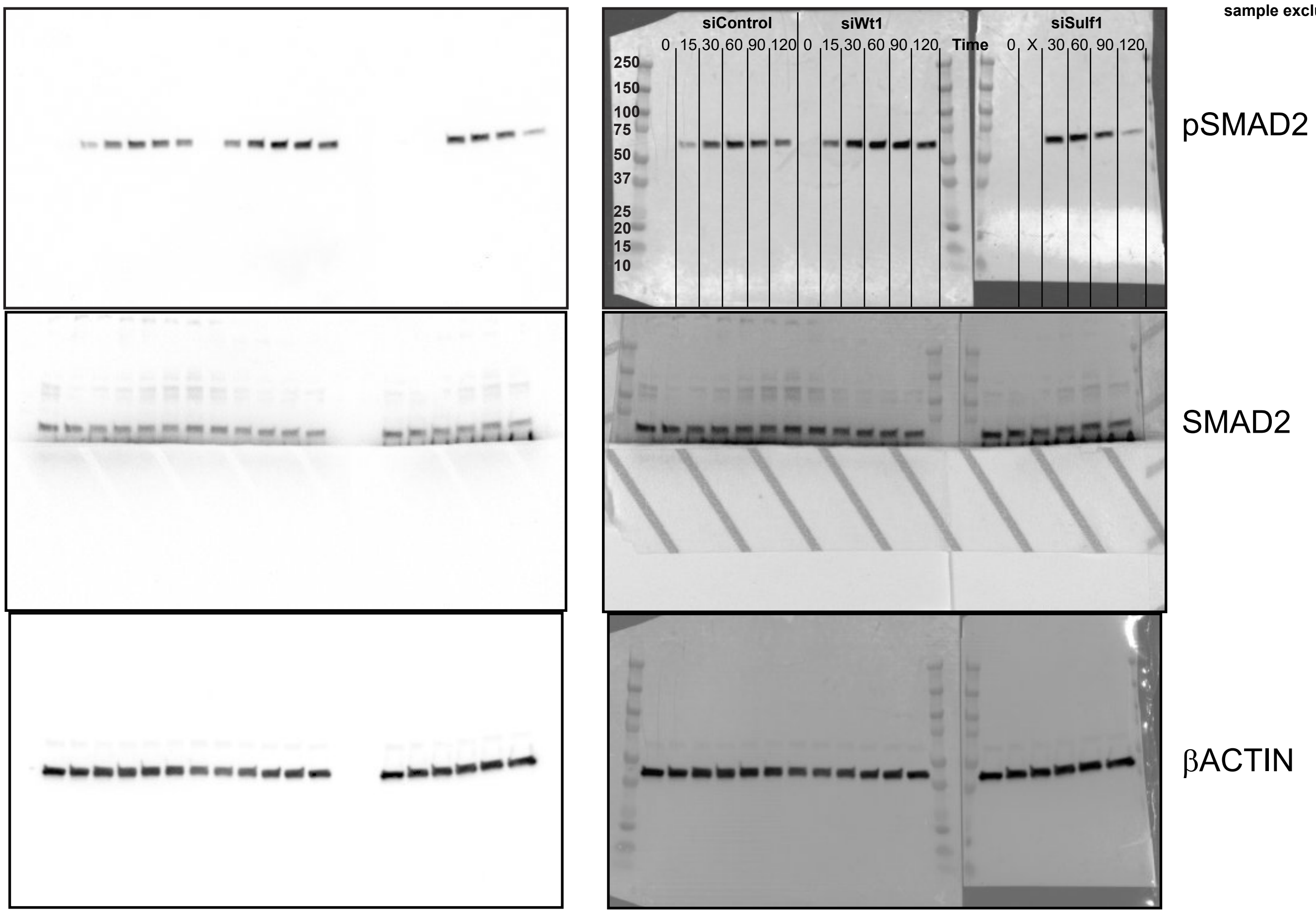

Supplement: Supplementary file 2 [file res-139-e327330-s003.pdf]
